# An Ion-Pair Induced Intermediate Complex Captured in Class D Carbapenemase Reveals Chloride Ion as a Janus Effector Modulating Activity

**DOI:** 10.1021/acscentsci.3c00609

**Published:** 2023-12-13

**Authors:** Qi Zhou, Pablo Catalán, Helen Bell, Patrick Baumann, Rebekah Cooke, Rhodri Evans, Jianhua Yang, Zhen Zhang, Davide Zappalà, Ye Zhang, George Michael Blackburn, Yuan He, Yi Jin

**Affiliations:** ¶ Key Laboratory of Synthetic and Natural Functional Molecule, College of Chemistry and Materials Science, Northwest University, Xi’an 710127, P. R. China; § Grupo Interdisciplinar de Sistemas Complejos, Departamento de Matemáticas, Universidad Carlos III de Madrid, 28911 Leganés, Spain; ‡ School of Chemistry, Cardiff University, Cardiff, CF10 3AT, United Kingdom; † Manchester Institute of Biotechnology, University of Manchester, 131 Princess Street, Manchester M1 7DN, United Kingdom; ¥ School of Biosciences, University of Sheffield, Firth Court, Western Bank, Sheffield S10 2TN, United Kingdom

## Abstract

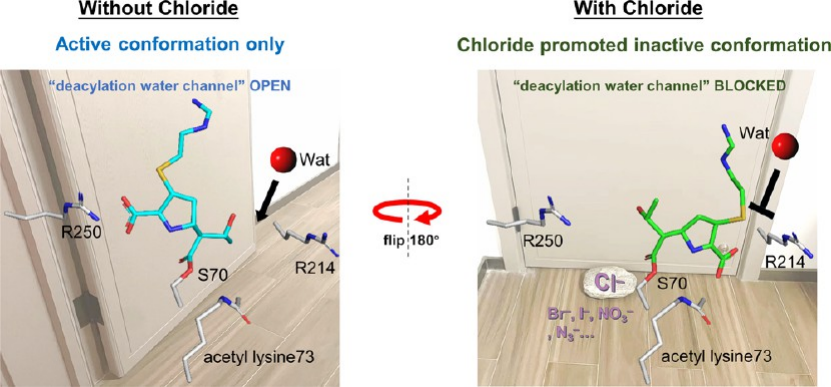

Antibiotic-resistant *Enterobacterales* that produce oxacillinase (OXA)-48-like Class D β-lactamases are often linked to increased clinical mortality. Though the catalytic mechanism of OXA-48 is known, the molecular origin of its biphasic kinetics has been elusive. We here identify selective chloride binding rather than decarbamylation of the carbamylated lysine as the source of biphasic kinetics, utilizing isothermal titration calorimetry (ITC) to monitor the complete reaction course with the OXA-48 variant having a chemically stable *N*-acetyl lysine. Further structural investigation enables us to capture an unprecedented inactive acyl intermediate wedged in place by a halide ion paired with a conserved active site arginine. Supported by mutagenesis and mathematical simulation, we identify chloride as a “Janus effector” that operates by allosteric activation of the burst phase and by inhibition of the steady state in kinetic assays of β-lactams. We show that chloride-induced biphasic kinetics directly affects antibiotic efficacy and facilitates the differentiation of clinical isolates encoding Class D from Class A and B carbapenemases. As chloride is present in laboratory and clinical procedures, our discovery greatly expands the roles of chloride in modulating enzyme catalysis and highlights its potential impact on the pharmacokinetics and efficacy of antibiotics during *in vivo* treatment.

## Abstract 2

Cl^−^ binding to conserved arginine of OXA-48-like carbapenemases promotes a misoriented intermediate, causing biphasic kinetics, impacting antibiotic efficacy, and enabling identification of Class D clinical isolates.

## Introduction

Antibiotic resistance is a major global threat to health.^1,2^ Carbapenems are clinically important β-lactam antibiotics with a broad spectrum of activity and high potency, often used as last-line agents for the treatment of serious infection.^3,4^ Three classes of carbapenemases have been identified: Ambler Class A (e.g., KPC), Class B (e.g., NDM), and Class D (oxacillinases, OXA type).^5,6^ Among these carbapenemases, OXA-48-like enzymes are widely disseminated and plasmid-encoded, and antibiotic resistance in OXA-48-producing *Enterobacterales* can lead to high clinical mortality rates.^7^ However, the lack of an effective and accurate clinical diagnosis of OXA-48-like carbapenemases poses a significant challenge to guiding precise clinical anti-infection therapy.^6,8^

**Figure 1:**
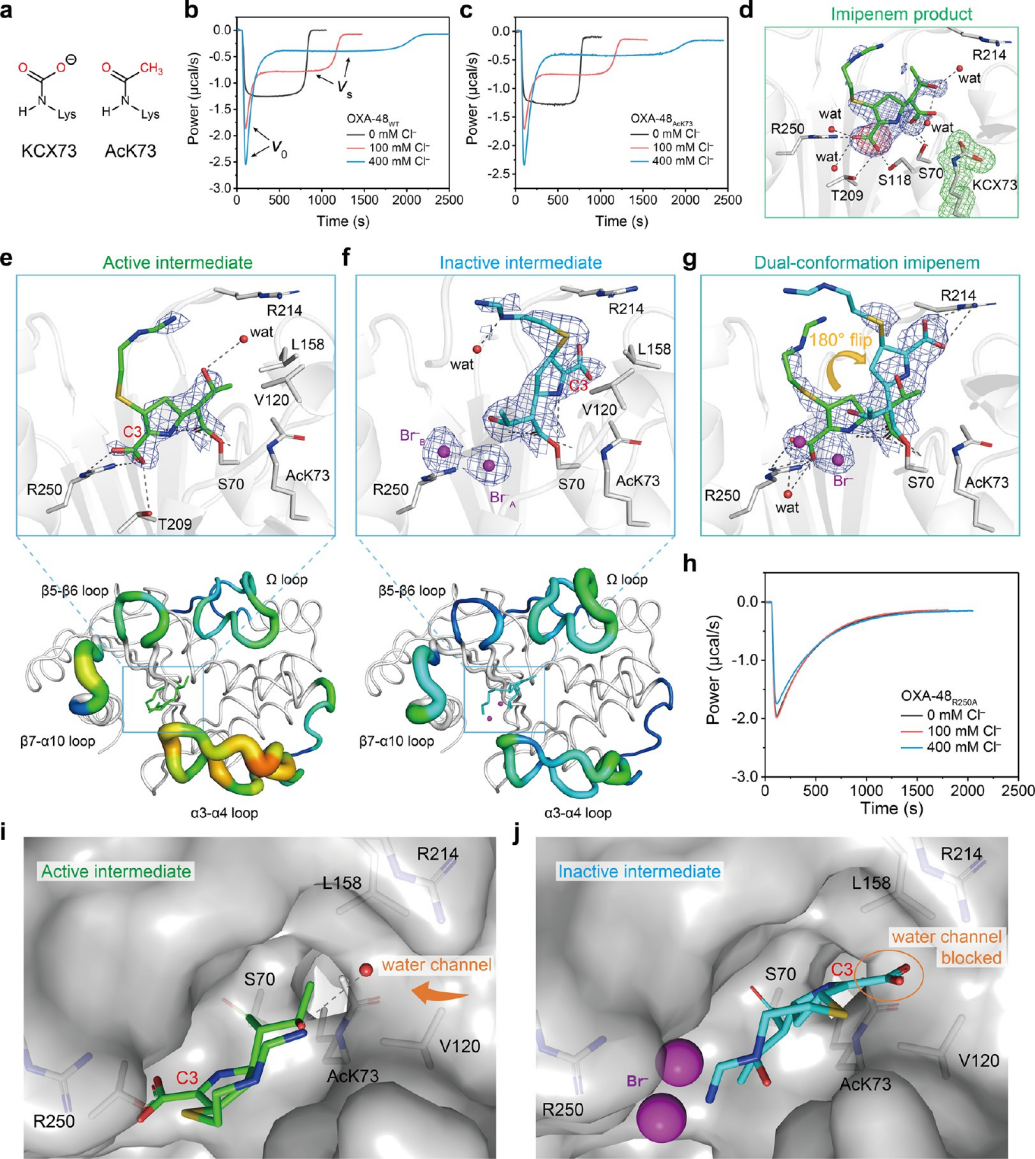
(**a**) Comparison of the structures of carbamylation (KCX73) and the acetylation (AcK73) modifications of K73. Effect of [Cl^–^] on the catalytic activity of (**b**) 100 nM OXA48_WT_ and (**c**) 8 μM OXA48_AcK73_ variant with 200 μM imipenem in a calorimetry assay, measured in 50 mM phosphate buffer, pH 7.5, and 1 mM NaHCO_3_. (**d**) Crystal structure of the OXA-48_WT_-imipenem-Br^–^ product complex obtained by soaking 32 mM imipenem with 500 mM NaBr in the apo OXA-48_WT_ crystal. This structure contains an anomalous signal for Br (red mesh, contoured at 3σ) overlaid with density for hydrolyzed imipenem, showing that the active intermediate conformation competes with the halide ion for R250 binding. The initial *F*_0_–*F*_c_ electron density maps of hydrolyzed imipenem (blue mesh) and KCX (green mesh) are contoured at 3σ (0.2796e^–^/Å^3^). (**e**, **f**) The crystal structures are captured in the (**e**) active intermediate and (**f**) inactive intermediate (a 180° acyl intermediate flip) states stabilized by bromide in OXA-48_AcK73_. The unbiased 2*F*_0_–*F*_c_ electron density map of imipenem is shown contoured at 1σ (blue mesh). Occupancies are 0.35 for Br_A_ and 0.4 for Br_B_. Cartoon putty representations of active intermediate and inactive intermediate monomers are drawn for the Ω loop, β5-β6 loop, β7-α10 loop and α3-α4 loop; *B*-factor values from lowest to highest are represented from blue to red. The size of the tube reflects the value of the *B* factor: the higher the *B* factor, the thicker the tube. Structures in other regions are in white and displayed in cartoon tube representation, where the size of the tube is independent of *B* factors. (**g**) Crystal structure of the OXA48_AcK73_-imipenem-Br^–^ acyl intermediate complex with dual-occupancy density from both active and inactive conformations in chain G. The initial unbiased 2*F*_0_–*F*_c_ electron density map of imipenem is shown (blue mesh) contoured at 1σ (0.2330e^–^/Å^3^). (**h**) Kinetic curves of 200 μM imipenem hydrolysis by the 2 μM OXA48_R250A_ variant with various [Cl^–^] values. (**i**, **j**) The water channel in the active intermediate (**i**) is blocked by the C3 carboxylate group of the flipped imipenem in the bromide-stabilized inactive intermediate (**j**).

We have previously developed a highly sensitive isothermal titration calorimetry (ITC) assay that enables efficient detection of carbapenemase-producing *Enterobacteriaceae* (CPE).^9^ ITC has a nonsubstitutable advantage in monitoring reaction rates in opaque cell cultures, detecting a wider range of substrates at various concentrations, providing precise control over reaction volume and temperature, and facilitating automation through multiple injections and rapid mixing. These attributes collectively enhance the reproducibility and accuracy for detecting subtle rate changes during β-lactam hydrolysis.^9^ We were able to show that, in Tris-HCl buffer at neutral pH, the imipenem hydrolysis by OXA-48 exhibited a burst phase followed by a slower steady phase minutes later. The partial inactivation of several OXA carbapenemases during turnover has been observed in the literature.^9−15^ It has been proposed that it is a result of the decarbamylation of the *N*-carbamylated catalytic lysine residue (KCX, Figure 1a), which acts as a general base and is critical for the acylation and deacylation steps of the reaction.^14,16,17^ Because of this, NaHCO_3_ has often been added routinely to the buffer to prevent the decarbamylation of KCX in biochemical experiments. However, our previous ITC experiments showed that the addition of 10 or 50 mM NaHCO_3_ (above the dissociation concentration of CO_2_^14,17^) led to a heightened rate acceleration during the burst phase, suggesting that the phenomenon extends beyond simple KCX saturation. Therefore, it became crucial to determine the molecular basis of the biphasic kinetics under physiological conditions and its relation to KCX decarbamylation to deliver a comprehensive understanding of the catalytic mechanism of OXA-48-like enzymes.

Studying the carbamylation state of lysine has always been challenging because of its labile nature.^18^ Notably, decarbamylation could be influenced by various factors, such as pH fluctuations during Raman spectroscopy, nuclear magnetic resonance (NMR) studies, or crystallization conditions^19−23^ (Table S1). Additionally, it has been suggested that the use of avibactam to mimic the acyl intermediate can also induce decarbamylation.^21,23,24^

In this study, we aim to shed light on the elusive nature of carbamylation on lysine in OXA-48 by incorporating the non-natural amino acid *N*-acetyl-lysine (AcK) into position-73 of the enzyme. Unexpectedly, our continuous ITC assay revealed chloride as the ultimate determinant of biphasic kinetics in OXA-48 reactions at physiological pH with a series of β-lactam substrates after we traced the complete reaction course under a variety of conditions. Using X-ray crystallography complemented by intrinsic fluorescence, we discovered an unprecedented “inactive intermediate” of the reaction in the OXA-48_AcK73_-imipenem complex structure, where the acyl-imipenem intermediate flipped 180° with a halide ion “wedged” between imipenem and key active site residue R250. Through mutagenesis and molecular docking, we established that R250 not only is important for substrate binding but also is the target for ion pair-induced enzyme inhibition by chloride. Our further mutagenesis study also identified that the binding of chloride to surface residues allosterically activates the enzyme. Based on the above evidence and mathematical simulation, we propose a new catalytic inhibition mechanism involving chloride ion as a “Janus effector” responsible for the biphasic kinetics. We demonstrated that the distinctive biphasic kinetics profile induced by chloride at neutral pH allows for the detection and discrimination of clinical CPEs encoding Class D OXA carbapenemases from Class A and B carbapenemases in a short turnover time. Additionally, we found that the growth of clinical isolates of *Klebsiella pneumonia* is affected by chloride concentration variation during antibiotic treatment.

Our findings have significant implications for designing new OXA inhibitors and preserving the effectiveness of current antibiotics. Chloride is an important electrolyte in life, with a concentration of between 15 and 120 mM,^25−27^ and is commonly used in buffers for kinetic assays, growth media for antibiotic susceptibility tests, and diluents for antimicrobial treatment. Thus, our work provides a molecular definition for guiding consistent kinetics and draws attention to the effect of chloride on the pharmacokinetics and efficacy of antibiotic drugs during *in vivo* treatment. Moreover, it will provide a more secure foundation for the growing application of molecular and quantum mechanical methods to the study of carbapenemases.

## Results and Discussion

### Discovery of Chloride as the Cause of Biphasic Kinetics

ITC assay allows us to continuously monitor the reaction course during substrate consumption by the direct measurement of thermal power that is proportional to the reaction rate.^28^ Previously, we have revealed a distinct biphasic calorimetric curve for OXA-48 hydrolysis of imipenem with Tris-HCl as the buffer,^9^ featuring an initial reaction rate burst followed by a prolonged steady rate. A more detailed investigation now shows that, in chloride-free buffers, only monophasic calorimetry curves with single-rate kinetics can be observed (Figure 1b). Increasing [Cl^–^] enhances the initial burst phase but increases inhibition of the second steady phase (Figure 1b, Figure S1). Thus, we determined IC_50_ for chloride and other halogen ions deduced from the steady inhibitory phase rate^10^*v*_s_ and found that, except for fluoride which shows no inhibition, the larger halide ions Cl^–^, Br^–^, and I^–^ all display stronger inhibition (Figures S2 and S3, Table S2). These data suggest that Cl^–^, Br^–^, and I^–^ modulate enzyme activity by a mechanism invalid for F^–^ and thus not simply by changing the ionic strength. We next tested the effect of other ions on OXA-48 catalysis. The cations Na^+^, K^+^, or NH_4_^+^ do not have any observed effect on the kinetics (Figure S4). Anions, including F^–^, SO_4_^2–^, and HCO_3_^–^ only boost the steady-state reaction rate and display monophasic reaction curves, whereas anions such as NO_3_^–^ and N_3_^–^ induce biphasic curves and likely exert a similar action on Cl^–^, Br^–^, and I^–^.

### Inhibitory Effect of Chloride Does Not Arise from Decarbamylation

To investigate the relationship between chloride-induced biphasic kinetics and lysine decarbamylation, we synthesized *N*-acetylated lysine (AcK, Figure 1a), a structurally similar but nonhydrolyzable mimic of the KCX, and incorporated it into the OXA-48_AcK73_ variant using the genetic code expansion method. High-resolution mass spectrometric (HR-MS) analysis showed 100% AcK incorporation (Figure S5). Michaelis–Menten kinetics for OXA-48_AcK73_ and OXA-48_WT_ measured by UV–vis spectroscopy (Figure S6) show a similar *K*_M_ (*k*_–1_/*k*_1_) for OXA-48_AcK73_ (13.89 μM) and OXA-48_WT_ (13.79 μM), meaning that AcK substitution does not interfere with substrate binding. However, the 40-fold decrease in *k*_cat_ for OXA-48_AcK73_ (0.13 s^–1^) compared to that for OXA-48_WT_ (5.13 s^–1^) shows the important contribution of KCX to catalysis. The calorimetric curves of OXA-48_AcK73_ in 50 mM phosphate buffer (pH 7.5 at 25 °C) consistently showed a typical monophasic calorimetric curve under Cl^–^-free conditions but a distinct biphasic calorimetric curve with chloride present (Figure 1c, Figure S7). Collectively, these observations demonstrate that the chloride-induced biphasic reaction curve is not due to KCX decarbamylation.

### Discovery of a Unique Inactive Acyl Intermediate with Halide Binding at R250

To reveal the effect of chloride on imipenem hydrolysis, we sought to obtain a complex structure of OXA-48 in the presence of halides and imipenem. We added 500 mM NaBr and 32 mM imipenem to the apo OXA-48_WT_ crystal and observed a clear Br^–^ anomalous signal overlapping with the density of imipenem, indicating that Br^–^ and imipenem compete for binding to R250 (Figure 1d, 1.53 Å resolution). Based on this observation, we carried out an additional structural investigation of OXA-48_AcK73_ as its significantly slow catalysis might deliver trapped key reaction intermediates. We soaked imipenem and bromide into OXA-48_AcK73_ crystals at neutral pH and solved the best structure to 2.15 Å resolution (Table S3). All eight chains showed clear electron density for the unbiased 2*F*_0_ – *F*_c_ maps of a covalent bond between imipenem and S70-OH (Figure 1e–g). Significantly, imipenem was bound in three different modes. In C, D, and E chains, imipenem is in a similar conformation to that observed in previously published OXA-48 acyl-enzyme intermediate structures (PDB 6P97,^29^6PTU,^22^ and 5QB4(30)) (Figure 1e). The imipenem carboxylate group forms strong salt bridges with the R250 side chain and an H-bond with T209-OH. The higher *B* factors in the β5-β6 loop, β7-α10 loop, and α3-α4 loop regions around the active site (Figure 1e) suggest an association with a higher catalytic activity of OXA-48.^31^ Since this conformation shows no bound bromide and has been observed in other acyl intermediate and product complex structures,^32^ we describe it as an active intermediate. No electron density for a deacylating water was observed in the near-attack conformation (NAC) probably because a hydrophobic methyl group of AcK sits in the place of a carboxylate O and disfavors binding of a nucleophilic water molecule. Given that the −OH moiety of the 6-α-hydroxyethyl group has been proposed to participate in directing water molecules into the active site through the “deacylating water channel”,^33^ the 6-α-hydroxyethyl group was intentionally fitted with its methyl group engaged in hydrophobic interactions with L158, while the −OH group points toward the protein surface, owing to the presence of observable water density within a 3 Å radius (Figure 1e, Figure S9a).

By contrast, in the A, B, and H chains, a bromide wedged between the imipenem intermediate and R250 results in a 180° acyl intermediate flip compared to the active intermediate, blocking the “water channel” of L158 and V120 in the deacylation step^22,23,29,33^ (Figure 1f,i,j). Furthermore, in both A and B chains the orientation of the imipenem carboxylate group leads to disorder of the Ω-loop with higher *B* factors due to spatial clashing with the C3 carboxylate group (Figure 1f), which is also considered to be detrimental to efficient deacylation^32^ and leads to a catalytic dead end. Thus, we describe this new bromide-R250 ion-paired intermediate as the inactivate intermediate. The bromide ion binding to the positive R250 side chain was found with partial occupancy. R250 has been proposed to play an important role in catalysis by binding to the carboxylate of the substrates.^34^ To further explore whether the binding of halides at R250 is a key driving force for the formation of the inactive acyl intermediate, we performed ITC analysis and showed that the OXA-48_R250A_ variant no longer responds to the presence of chloride with biphasic features but the nonconserved OXA-48_R214A_ variant still does (Figure 1h, Figure S8). These data indicate that the binding of chloride to R250 is essential for chloride-induced biphasic kinetics. In F and G chains, a dual-occupancy electron density of both active and inactivate intermediates was observed (Figure 1g), providing evidence that these two intermediate forms are interconvertible with distinct active-site dynamics. This was further confirmed in a structure obtained by soaking imipenem into Cl^–^-free OXA-48_AcK73_ crystals in which we saw both inactive and active acyl intermediates (Figure S9).

### Direct Observation of Chloride-Induced Conformational Changes

**Figure 2:**
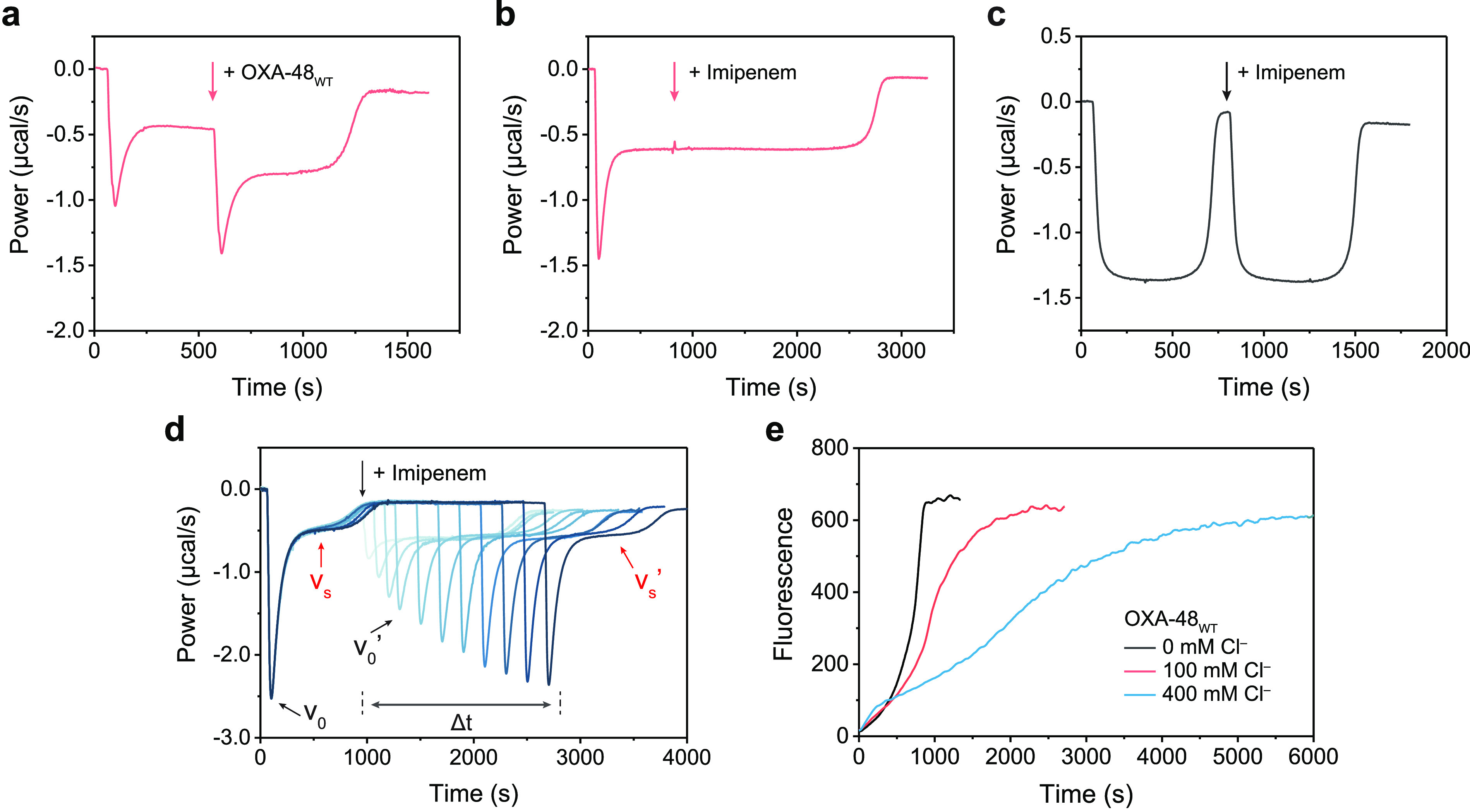
Effects of adding (**a**) fresh enzyme or (**b**) fresh substrate to the steady-state phase of the reaction on the biphasic kinetics with 100 mM Cl^–^. (**c**) Two successive additions of 200 μM fresh imipenem to 100 nM OXA-48_WT_ solution in Cl^–^-free buffer. (**d**) Overlay of ITC kinetic curves for titration of the time interval between two additions of 140 μM fresh imipenem to 100 nM OXA-48_WT_ solution with 400 mM Cl^–^. (**e**) Effect of [Cl^–^] on the intrinsic fluorescence curves of 200 nM OXA-48 hydrolysis of 400 μM imipenem. The fluorescence did not recover to the original intensity as the hydrolyzed imipenem quenches the intrinsic fluorescence in a concentration-dependent fashion (shown in Figure S10). All buffers used for ITC and fluorescence assays are 50 mM sodium phosphate pH 7.5 with 1 mM NaHCO_3_.

To elucidate whether the observed inactive conformation in crystallo is linked to catalytic steady-state inhibition, we added fresh OXA-48 enzyme or imipenem to a reaction that has proceeded to the steady phase and showed that the addition of fresh OXA-48 enzyme reinitiated the biphasic reaction (Figure 2a), whereas more imipenem did not initiate a biphasic process (Figure 2b). This shows that the equilibrium of the two intermediate conformations with different reactivities is established in the steady state. We next carried out double-injection ITC experiments to explore the shift in this equilibrium. When the reaction solution has no chloride, the calorimetric curves of the two injections are almost identical (Figure 2c), showing that the turnover capacity of the enzyme remains the same and there is no product inhibition. By contrast, with 100 and 400 mM Cl^–^ in the reaction solution, the addition of fresh imipenem results in time-dependent changes in the calorimetric curves. As the time interval is increased, the initial rate of the new reaction *v*_0_′ of the second reaction gradually increases until it fully recovers to the original *v*_0_ (Figure 2d, dark-blue line; Figure S10), which suggests that the concentration of free enzyme increases with time, leading to apparent enzyme reactivation. This further confirms that chloride-induced biphasic kinetics is related to a slow redistribution of the equilibrium between the two intermediate conformations with different reactivities. Combining the structural evidence with solution data, we propose that for the chloride-free reaction of OXA-48 the *active intermediate* predominates and delivers turnover. After supplementation of chloride, binding of chloride wedged between the imipenem intermediate and R250 results in progressive rate retardation from the accumulation of the *inactive intermediate* formed during catalysis until a new *active–inactive* equilibrium is established to deliver the slower steady-state kinetics observed.

To directly monitor chloride-induced conformational changes of OXA-48 in real time, we used intrinsic tryptophan fluorescence.^35,36^ The several tryptophan residues near the active site include W105, 157, 221, and 222, of which W105 on the α3-α4 loop of OXA-48 has a direct interaction with imipenem. When we excited OXA-48 solution (λ_ex_ 280 nm), it showed a stable fluorescence intensity at 340 nm (Figure S11). Upon addition of substrates at *t* = 0, the intrinsic OXA-48 fluorescence was immediately quenched (Figure 2e). Under Cl^–^-free conditions, the fluorescence quickly recovered as soon as the substrate was consumed at around 750 s (Figure 2e, black curve). In contrast, when chloride was present, we observed that the fluorescence recovery was initially faster but dramatically slowed in the period of 500 s to 100 min. This shows intrinsic fluorescence detects more subtle underlying dynamics from the two intermediate conformations and identifies an unexpectedly long lag time for OXA-48 to return to its original conformation, even after the substrate has been completely consumed (shown by ITC in Figure 2d).

### Positive Allosteric Effect Originates from Halide Binding at the Interface

**Figure 3:**
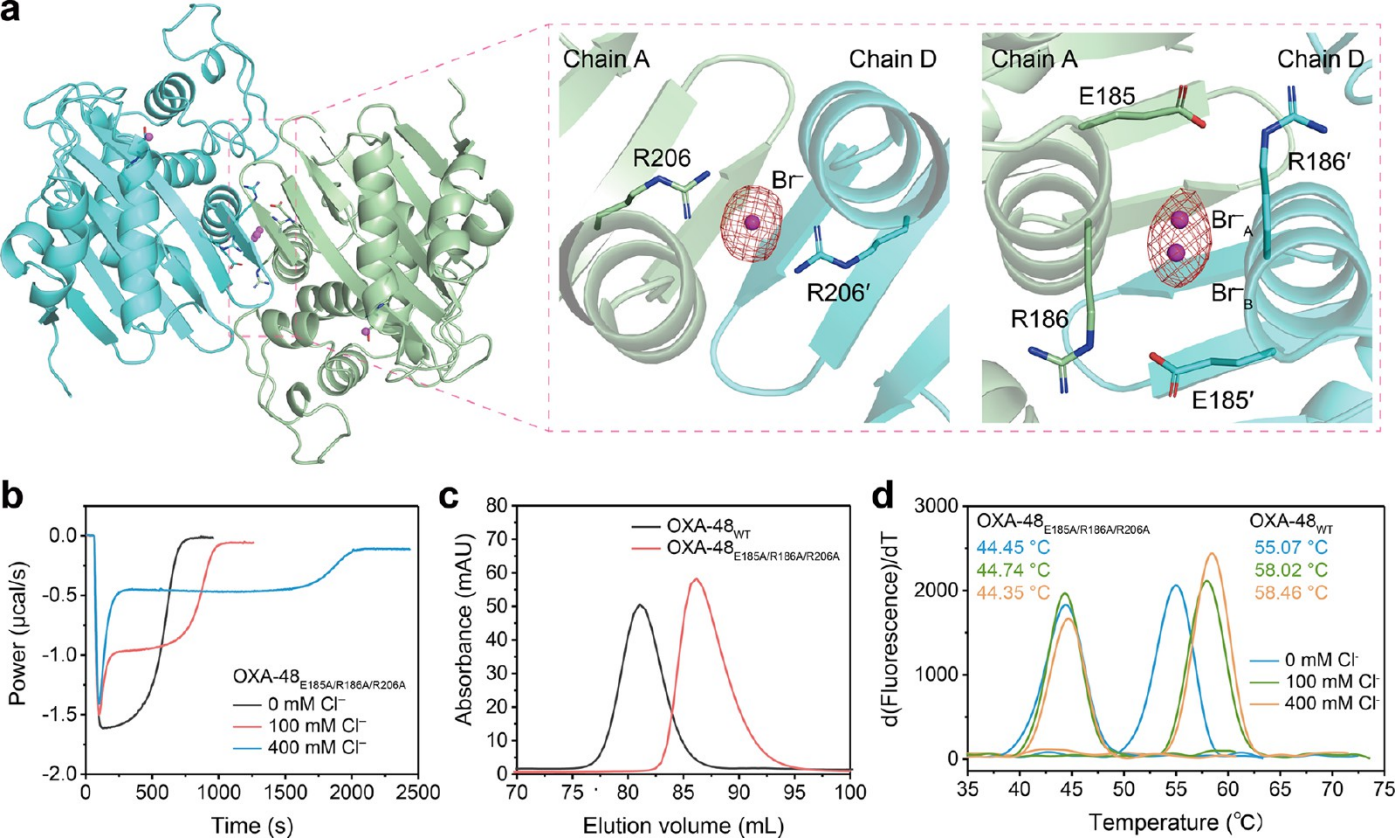
(**a**) Cartoon to show the dimeric assembly from the crystal structure of the OXA-48_WT_-Br^–^ complex; bromide ions bound on the dimer interface and in the active sites are shown in purple spheres. Binding sites for halide ions at the OXA-48_WT_ enzyme dimer interface (anomalous map of Br is contoured at 3σ, red mesh). (**b**) Kinetic curves of 200 μM imipenem hydrolysis by 100 nM interface variant OXA-48_E185A/R186A/R206A_ with various [Cl^–^] values in 50 mM sodium phosphate buffer, pH 7.5, and 1 mM NaHCO_3_. (**c**) Size exclusion chromatography of OXA-48_WT_ and the interface variant OXA-48_E185A/R186A/R206A_, run on a HiLoad 16/600 Superdex 200 pg column in 50 mM sodium phosphate buffer, pH 7.5. (**d**) Protein thermal shift melting curves for OXA-48_E185A/R186A/R206A_ and OXA-48_WT_ with variable concentrations of chloride ion.

Compared to the rate of hydrolysis from the monophasic curve in the chloride-free buffers, all of the biphasic curves induced by chloride (and Br^–^, NO_3_^–^, and N_3_^–^) have a fast burst phase before inhibition kicks in in the steady phase (Figure S4), demonstrating that these ions have a complex role. The molecular mechanism of the burst phase requires explanation. One possible mechanism could be via “chemical rescue” similar to “azidolysis” by better nucleophiles to form an activated acyl-halide or acyl-azide intermediate before faster deacylation by water occurs. However, the observation that anions such as NO_3_^–^ and highly solvated F^–^ exhibit limited nucleophilic reactivity yet are capable of inducing the burst phase eliminates covalent catalysis as a burst-phase mechanism (Figure S12). To further identify the molecular origin of the first phase acceleration by some anions including halides, we solved an OXA-48_WT_ apo structure at neutral pH (1.92 Å resolution, Table S3) and a structure of dimeric OXA-48_WT_ with bromide bound (1.81 Å resolution, Figure 3a, Table S3). The anomalous signal from bromide (Figure 3a, red mesh) clearly identified two bromides bound at the previously identified OXA-48 dimer interface,^37^ namely, between side chains of R206-R206′ and the side chains of E185-R186-E185′-R186′. The iodide-bound OXA-48_WT_ structure shows the same binding sites (Figure S13). To prove these binding sites could be potential allosteric sites responsible for the observed kinetics, we prepared and crystallized interface variant OXA-48_E185A/R186A/R206A_ before soaking Br^–^ into these crystals. The refined structure showed that halide binding at these sites was abolished (Figure S14). OXA-48_E185A/R186A/R206A_ also showed a significantly smaller burst phase *v*_0_ in biphasic kinetics than OXA_WT_ (Figure 3b, Figure S15), suggesting that chloride binding to these interfacial residues accounts for the positive allosteric effect. Size exclusion chromatography revealed that OXA-48_WT_ is a strongly stabilized dimer, while interface variant OXA-48_E185A/R186A/R206A_ exists as a monomer or as a weakly associated dimer in solution (Figure 3c). Protein thermal shift assays showed that *T*_m_ for OXA-48_WT_ is 55 °C in the pH 7.5 Cl^–^-free buffer, i.e., 10 °C higher than that of OXA-48_E185A/R186A/R206A_ (Figure 3d). The addition of chloride further increases the *T*_m_ of OXA-48_WT_ by ∼3.5 °C but does not do so for OXA-48_E185A/R186A/R206A_ (Table S4). Our present data suggest that chloride binding to E185, R186, and R206 at the enzyme dimer interface endows the enzyme with higher stability and also leads to a greater allosteric effect in the burst phase.

### Chloride Ions as a Janus Effector Induce Biphasic Kinetic Behavior

**Figure 4:**
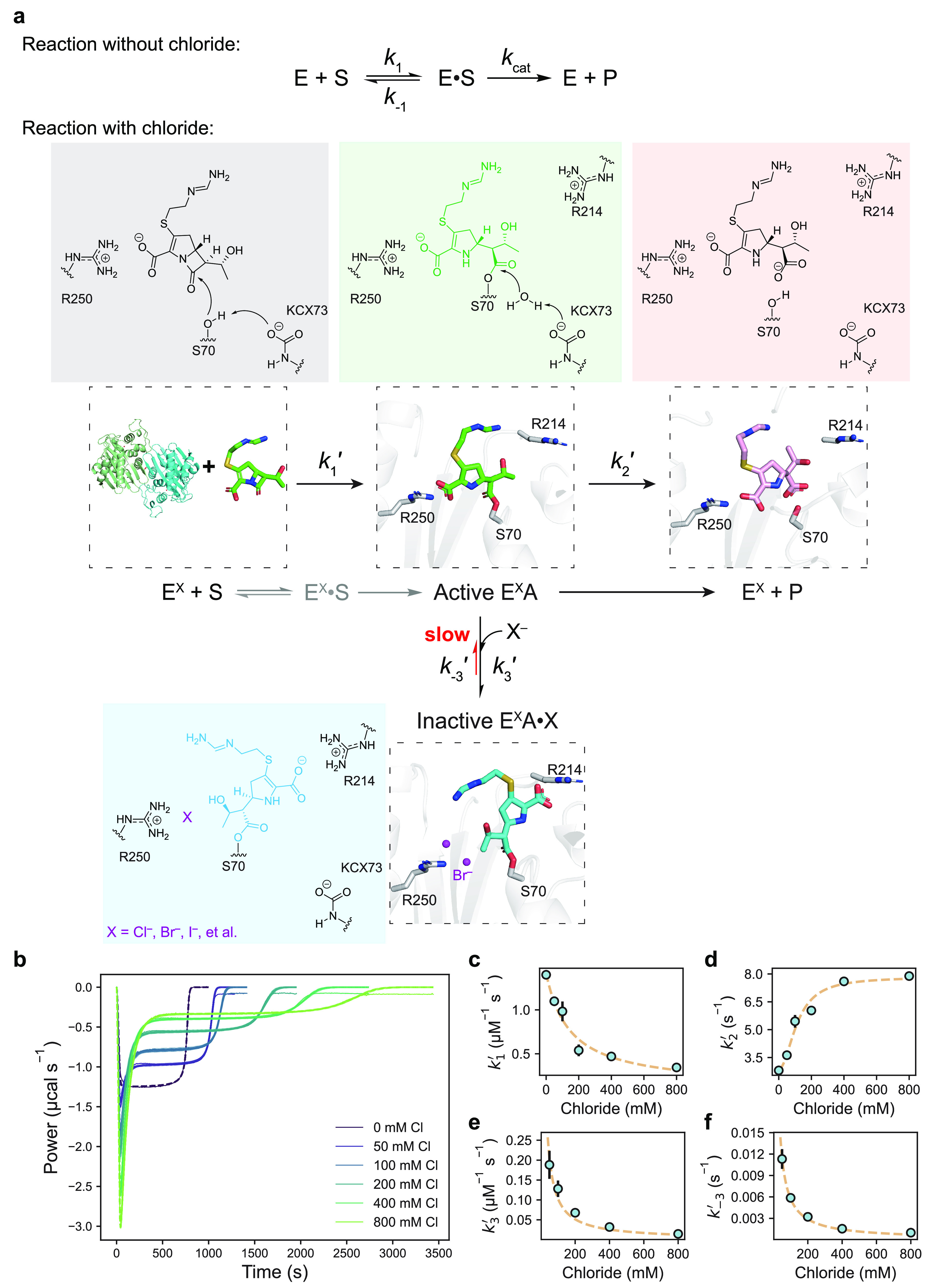
(**a**) Mechanism explaining biphasic hydrolysis with the catalytic inhibition by halides. E – enzyme, S – substrate; E^X^ – halides bound at the interface of the OXA48 dimer, S – substrate, P – product; active E^X^A – acyl-enzyme intermediate; inactive E^X^A·X – halides bound at R250 at an inactive conformation. (**b**) Kinetic modeling and simulation for biphasic hydrolysis. (**c**–**f**) Apparent rate constants *k*_1_′, *k*_2_′, *k*_3_′, and *k*_–3_′ and their dependence on [Cl^–^].

We now propose a new mechanism with Cl^–^ as a Janus effector to exert a “positive allosteric effect” but a “catalytic inhibitory effect”^38^ as an explanation of the observed biphasic kinetics (Figure 4a). Given that allostery and inhibitory effects are inseparable, it is not possible to fit our ITC data solely to a “slow-binding”^39^ or “covalent”^40^ inhibitor model, especially when we could not measure the concentrations of substrate-bound and acylated intermediates separately. To delineate the chloride-dependent steps, we condensed the reaction scheme involving chloride into four distinct stages: *k*_1_′ is the rate constant governing substrate conversion to the active acyl complex (E^Cl^A) with enzyme having chloride bound at the dimer interface (E^Cl^); *k*_2_′ is the rate constant for the deacylation of E^Cl^A to yield the product; while *k*_3_′ and *k*_–3_′ represent the association constant and dissociation constant for the formation of inactive acyl complex E^Cl^A·Cl via chloride binding at R250 of E^Cl^A. Mathematical analysis of ITC data from Figure 4b and Figure S10 showed that all four rate constants are affected by chloride (Figure 4c–f, Table 1, and Figures S16–S21). For OXA-48_WT_, *k*_1_′ decreases as [Cl^–^] increases (Figure 4c, Table 1), meaning that acylation slows down when there is more Cl^–^ around. However, the magnitude of *k*_2_′ for turnover increases with chloride concentration because of a more positive allosteric effect (Figure 4d). *k*_3_′ is 1 order of magnitude smaller than *k*_1_′, meaning that at the beginning of the burst of phase the inhibition is negligible. The equilibrium dissociation constant *k*_–3_′/*k*_3_′ is unchanged, meaning that this step must be at equilibrium in the steady state with a prolonged steady rate following the initial burst^38^ (Figure 4c,d). It also explains why higher [Cl^–^] has a larger catalytic inhibition effect. A comparison of the trend of changes for OXA-48_E185A/R186A/R206A_ to those for OXA-48_WT_ (Figure S22, Table 1) showed that chloride bound at the interface mainly affects *k*_1_′ and *k*_2_′ while chloride binding to R250 has larger influences on *k*_3_′ and *k*_–3_′. Based on the intrinsic fluorescence experiments that there is slow recovery of active enzyme conformation after all substrates had been consumed (Figure 2e), *k*_–3_′ is likely to be the rate-limiting step of the reaction when chloride is present.

**Table 1:** Apparent Rate Constants for OXA-48_WT_ and OXA-48_E185A/R186A/R206A_

	[Cl] (mM)	*k*_1_′ (μM^–1^ s^–1^)	*k*_2_′ (s^–1^)	*k*_3_′ (μM^–1^ s^–1^)	*k*_*–*__3_′ (s^–1^)
OXA-48_WT_	0	1.40	2.81	-	-
	100	0.98	5.31	0.121	0.0058
	400	0.47	7.62	0.032	0.0015
OXA-48_E185A/R186A/R206A_	0	0.31	4.48	-	-
	100	0.36	5.26	0.126	0.0126
	400	0.31	4.96	0.046	0.0050

### Validation of the Proposed Mechanism

To validate the proposed mechanism, a series of β-lactam substrates were chosen for kinetic studies with OXA-48, including cefamezin, oxacillin, penicillin, and cefotaxime. Our data show conclusively that unless chloride ions are included in the buffer there is no biphasic kinetic behavior of OXA-48 with these substrates, even for substrates with a considerably large substituent chain group^14,41^ (Figures S23–S25). This opposes the previous opinion that β-lactams with sterically encumbered side chains at their α-carbon, such as cloxacillin, oxacillin, and carbenicillin, can induce a conformational change in OXA-10,^14^ OXA-27,^12^ and OXA-2^10^ as well as in some other β-lactamases^41^ to cause biphasic kinetics. By molecular docking using the H-chain of the OXA-48_AcK73_-imipenem structure containing a bromide ion, oxacillin, penicillin G, and cefotaxime all display an inactive intermediate binding mode, with its α-carbon substituent group on the lactam forming more prominent hydrophobic interactions with residues I102, Y117, and S244 (Figures S23b,d and S24). This closely resembles that of imipenem in its inactive conformation, further obstructing the deacylating water channel. This is in marked contrast to the binding mode of oxacillin in our active acyl intermediate OXA-48_AcK73_-oxacillin structure in the absence of halide (Figure S23a). Our data suggest that chloride promotes the formation of an inactive acyl intermediate, which may be a recurring phenomenon. Therefore, our new model for catalytic inhibition by chloride is likely to be widely represented in the OXA-48 hydrolysis of a variety of substrates.

### Impact of Chloride on the Specific Detection of OXA-48 Producers and on the Efficacy of Antibiotics

**Figure 5:**
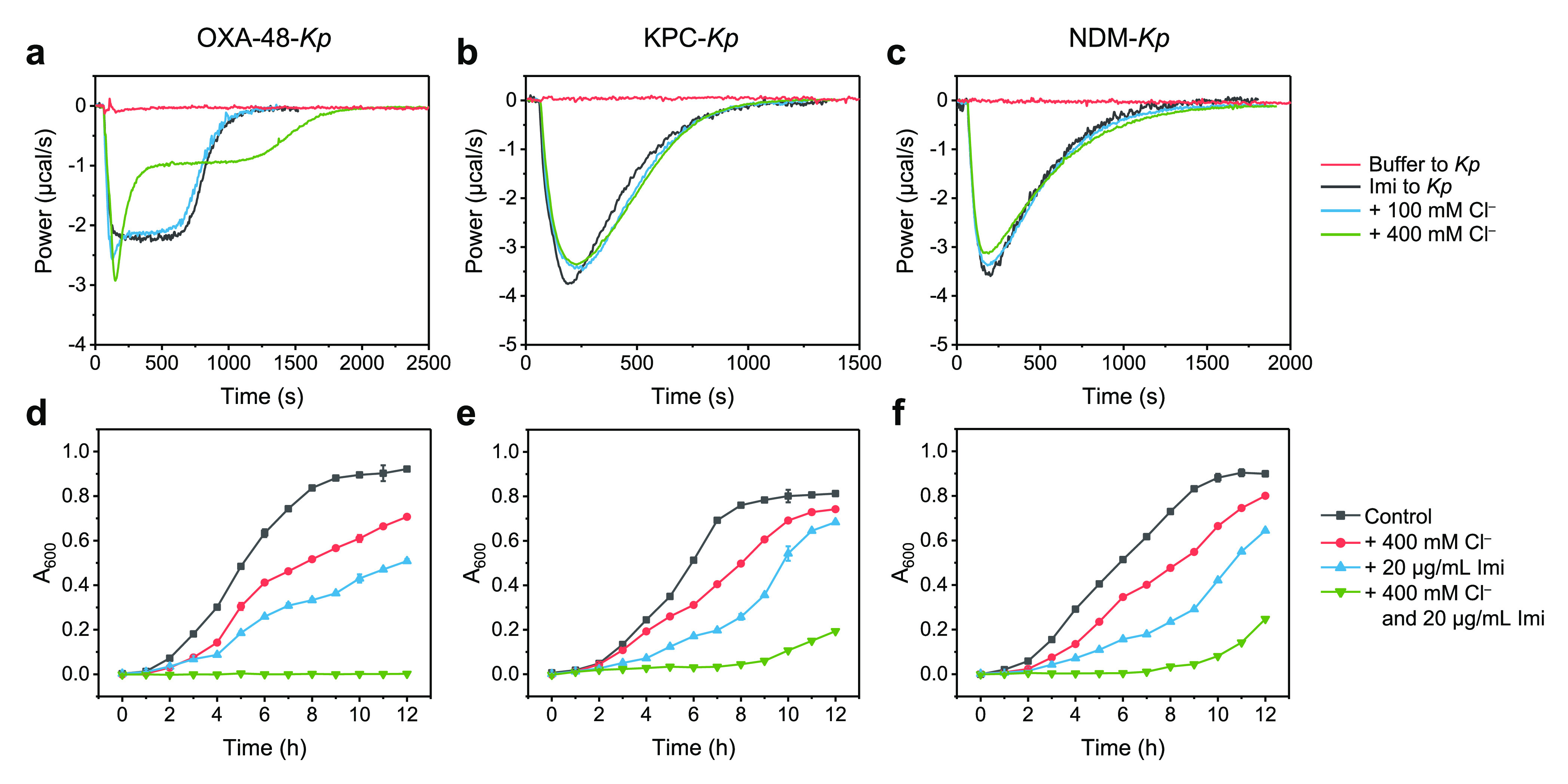
Thermogram curves of 400 μM (127 μg/mL) imipenem hydrolysis in the absence and presence of chloride by cell suspensions of (**a**) OXA-48-*Kp* (OD_600_ = 4.0), (**b**) KPC-*Kp* (OD_600_ = 1.5), and (**c**) NDM-*Kp* (OD_600_ = 0.1) in 50 mM sodium phosphate buffer, pH 7.5, and 0.1 mM ZnSO_4_. Growth curves of (**d**) Class D OXA-48-*Kp* bacteria, (**e**) Class A KPC-*Kp* bacteria, and (**f**) Class B NDM-*Kp* bacteria under variable conditions (400 mM NaCl and 20 μg/mL imipenem in TB media), all starting from OD_600_ 0.0006 (a starter culture with OD_600_ = 0.6 diluted by 1000-fold into the TB media).

We then carried out living-cell ITC studies^9^ to compare calorimetric curves of imipenem hydrolysis by OXA-48-encoding *K. pneumoniae* under chloride-present and chloride-free conditions. The calorimetric curves were recorded when imipenem was titrated directly into cell suspensions. The inclusion of 400 mM chloride in a phosphate buffer led to a biphasic curve of OXA-48-encoding *K. pneumoniae* and BL21 Star (DE3) *E. coli* (Figure 5a, Figure S26a) but showed no influence on the curves for both KPC-encoding and NDM-encoding *K. pneumoniae* and *E. coli* (Figure 5b,c, Figure S26b,c). This demonstrates that biphasic kinetics is uniquely caused by chloride in Class D OXA-48 and can be used for specifically discriminating the Class D OXA-48-producer from CPEs encoding Class A and B carbapenemases.

Since chloride can specifically modulate the OXA-48 hydrolysis of antibiotics, we then sought to test if the addition of chloride will affect the efficacy of antibiotics by monitoring the growth curves of three prevalent CPE *Klebsiella pneumoniae* strains producing OXA-48 (Class D), KPC (Class A), and NDM (Class B), respectively, under different conditions (Figure 5d–f). The synergistic effect of chloride and imipenem is most significant for *K. pneumoniae* encoding OXA-48, showing no growth within 12 h (Figure 5d). The results indicate that the coadministration of chloride and antibiotics has the potential to influence the efficacy of antibiotics in treating drug-resistant OXA-48-encoding *K. pneumoniae*, likely because of chloride acting as a Janus effector upon OXA-48 hydrolysis.

## Conclusions

Investigating the catalytic mechanism of carbapenemases is essential for developing new antibiotics and detection methods to combat antibiotic resistance. However, the molecular mechanism underlying the inactivation of clinically important OXA-48-like enzymes during catalysis remains unresolved. In this study, we have discovered that chloride is the real cause of biphasic kinetics observed by us and by others for OXA-48 catalysis with a series of β-lactam substrates. Our data and analysis thereof show that chloride ion acts as a Janus effector: it delivers allosteric activation by binding at the dimeric enzyme interface while independently causing the inhibition of steady-state catalytic activity by misorientation of a reactive intermediate by ion pairing with R250 in the active site. This identification of a significant role for the highly conserved R250 in chloride binding (Figure S27, Table S5) and in enzyme catalysis may provide a novel target site for designing new antimicrobial agents or formulations for treating bacterial infections. In addition, we demonstrated that chloride-induced biphasic kinetics is a typical feature of Class-D OXA-48 hydrolases, which can now be used to develop specific assays to discriminate bacteria harboring OXA-48-like carbapenemases from other CPEs.

While Cl^–^ binding has been previously implicated as a potential causal factor in decarbamylation, our findings indicate that Cl^–^ itself does not directly induce decarbamylation, as evidenced by our structural analyses (Figure 1d and Figure S28a,b, 7O5T for OXA-48_WT_-Br^–^, 7NRJ for OXA-48_WT_-I^–^, and 8QNZ for OXA-48_WT_-Br^–^-hydrolyzed imipenem product complexes). Instead, the previously observed Cl^–^ binding between K73 and W157 emerges as a consequence of decarbamylation events. Under acidic conditions, decarbamylation is presumed to occur,^14,16^ potentially leading to an entirely distinct mechanism where chloride may play a different role. Avibactam, which is commonly used as an intermediate mimic, may also trigger decarbamylation^20,23^ as demonstrated in our OXA-48_WT_-avibactam structure (PDB: 7O5N), wherein Cl^–^ binds between a decarbamylated K73 and W157 even under neutral pH crystallization conditions. The crystal structure of OXA-48_WT_-HCO_3_^–^ (Figure S28c,d) shows how HCO_3_^–^ binds to both R206 and R206′ at the dimer interface and with R250 in the active site. However, bicarbonate is unlikely to act as a molecular wedge because its size would cause it to clash with the reaction intermediate (Figure S29). Hence, our kinetic analysis shows that HCO_3_^–^ is only a weak albeit positive allosteric effector of OXA-48, possibly by binding at the enzyme surface (Figure S30). Indeed, a competitive binding assay using solution ^19^F NMR shows that an increase in [HCO_3_^–^] from 25 to 100 mM totally outcompeted surface-bound F^–^ at 500 mM for the same anion binding sites (Figure S31). In addition, the hydrolytic activities of both OXA-48_WT_ and OXA-48_AcK73_ are doubled when the bicarbonate anion concentration is increased from 0 to 400 mM (Figure S30), thus establishing that, apart from preventing decarbamylation,^14,42^ a high concentration of HCO_3_^–^ also contributes to rate enhancement via a positive allosteric effect. Along with HCO_3_^–^, we also show here that SO_4_^2–^ is exclusively a positive allosteric effector and has no inhibitory effect. By contrast, both NO_3_^–^ and N_3_^–^ induce biphasic kinetics similar to those of Cl^–^ and Br^–^ (Figure S4). These differences may well be a consequence of the variable energy of desolvation that ionic species have to overcome on binding to the protein, which may significantly affect their wedging ability.^43^

Given the ubiquitous nature of chloride ions, this study demonstrates the importance of careful attention to avoid inconsistent results in enzyme kinetics measurements, MIC assays,^44^ and antimicrobial treatment where NaCl solution is widely employed as the preferred salt or diluent for antibiotic work. It will also provide a broader experimental foundation for investigations of this class of carbapenemases by molecular dynamics, QM/MM, and free-energy calculations.^33,45^ Finally, our investigation significantly expands our understanding of the Janus effector role of especially halide anions in modulating enzyme catalysis^46^ while at the same time providing valuable experimental information in the fight against antibiotic-resistant bacteria.

## Data Availability

Coordinates have been deposited in Protein Data Bank with accession codes 7PEH, 7O5T, 7NRJ, 7O9N, and 8QNZ for the OXA-48_WT_ apo, OXA-48_WT_-Br^–^, OXA-48_WT_-I^–^, OXA-48_WT_-HCO_3_^–^, and OXA-48_WT_-Br^–^-product (dual occupancy) complexes, respectively; 7PEI for the OXA-48_E185A/R186A/R206A_-CI^–^ complex and 7PGO for the OXA-48_R250A_-Br^–^ complex; and 7Q14, 7PFN, and 7PSE for the OXA-48_AcK_-imipenem-Br^–^ complex, OXA-48_AcK_-imipenem complex, and OXA-48_AcK_-oxaciline complex, respectively.
