# Supplementary material for: An Ion-Pair Induced Intermediate Complex Captured in Class D Carbapenemase Reveals Chloride Ion as a Janus Effector Modulating Activity
Source: ACS Cent Sci. 2023 Dec 13;9(12):2339–49. doi: 10.1021/acscentsci.3c00609 (PMC10755735; doi:10.1021/acscentsci.3c00609)
Supplement: Supplementary file 1 — oc3c00609_si_001.pdf [file oc3c00609_si_001.pdf]

Supplementary information for

An Ion-Pair Induced Intermediate Complex Captured in Class D Carbapenemase Reveals Chloride Ion as a Janus Effector Modulating Activity

Qi Zhou[¶∇], Pablo Catalán[§∇], Helen Bell[‡∇], Patrick Baumann[‡†], Rebekah Cooke[‡], Rhodri Evans[‡†], Jianhua Yang[¶], Zhen Zhang[¶‡], Davide Zappalà[‡], Ye Zhang[¶], George Michael Blackburn[¥], Yuan He[¶*], Yi Jin[‡†*].

[¶] Key Laboratory of Synthetic and Natural Functional Molecule, College of Chemistry and Materials Science, Northwest University, Xi'an, 710127, P. R. China

[§] Grupo Interdisciplinar de Sistemas Complejos, Departamento de Matemáticas, Universidad Carlos III de Madrid, 28911 Leganés, Spain

[‡] School of Chemistry, Cardiff University, Cardiff, CF10 3AT, United Kingdom

[¥] School of Biosciences, University of Sheffield, Firth Court, Western Bank, Sheffield, S10 2TN, United Kingdom

[†] Manchester Institute of Biotechnology, University of Manchester, 131 Princess Street, Manchester, M1 7DN, United Kingdom

[∇] These authors contributed equally to this work.

* Corresponding authors: Yuan He and Yi Jin.

Email: yuanhe@nwu.edu.cn, yi.jin@manchester.ac.uk

Table of Tables:

Table S1 Survey of all OXA-48 structures in Protein Data Bank (PDB) (up to November 2022). The correlation of the pH, resolution, the carbamylation status of the Lys73, and any presence of halide ions....................4
Table S2 EC_{50} and IC_{50} Values of OXA-48 for all four halide ions. 6
Table S3 Data collection and refinement statistics 7
Table S4 T_m value of OXA-48_{WT} and OXA-$48_{E185A/R186A/R206A}$ with chloride. 10
Table S5 Sequence alignment for OXAs that have been reported to display biphasic kinetics..................11
Table S6 Oligonucleotides used for sequencing and mutagenesis. 39

Table of Figures:

Figure S1 Effect of enzyme, substrate or chloride concentrations on biphasic kinetics in ITC assays. 13
Figure S2 Effect of four halide ions at 100 mM on hydrolysis of imipenem by OXA-48. 14
Figure S3 IC_{50} plots of (**d**) chloride, (**e**) bromide and (**f**) iodide to reflect the inhibiting effect during imipenem hydrolysis by OXA-48_{WT}..14
Figure S4 The effect of ITC kinetics curves induced by various common cations and anions....................15
Figure S5 Mass spectrometry of OXA-48_{AcK73}. 16
Figure S6 Michaelis-Menten kinetics were measured in triplicate for OXA-48_{AcK73} variant (**b**) to compare with OXA-48_{WT} (**a**) by UV-vis method at 25 °C in the buffer of 100 mM NaPi, 50 mM $NaHCO_3$, pH 7.5, with strictly no chloride..16
Figure S7 UV-Vis for monitoring imipenem hydrolyzed by OXA-48 to show it is compatible with ITC, and comparison of the ITC heat profile to show the fast-mixing deadtime is negligible when the order of injection was changed. 18
Figure S8 ITC kinetic curves of 400 μM imipenem hydrolysis by 3 μM OXA-48_{R214A} variant with 0 mM, 100 mM or 400 mM chloride ion in the buffer of 50 mM NaPi, pH 7.5, and 1 mM $NaHCO_3$. 19
Figure S9 The crystal structure of chloride-free OXA-48AcK-imipenem acyl-intermediate complex. 20
Figure S10 Overlays of kinetics curves for titrating different time intervals between two imipenem injections into OXA-48 in order to show the time required for the initial activity recovery before the 2nd injection. 21
Figure S11 The intrinsic tryptophan fluorescence spectra of OXA-48_{WT} to show the intensity at λ_{em} = 340 nm is only affected by the concentration of hydrolyzed imipenem product, but not by other components in the assay. 22
Figure S12 Kinetic curves for imipenem hydrolysis by OXA-48_{WT} with 0 mM, 100 mM, and 400 mM F^-. F^- only affects the hydrolytic rate through the allosteric effect, using the surface variant OXA-$48_{E185A/R186A/R206A}$ as a control..23
Figure S13 Crystal structure of OXA-48_{WT} with iodide ions. 23
Figure S14 Crystal structures of OXA-$48_{E185A/R186A/R206A}$ (7PEI, **a,b**) and OXA-48_{R250A} (7PGO, **c**) soaked with Br^- to confirm the abolished bromide binding after mutating these residues to alanine...............................24
Figure S15 Comparing the OXA-48_{WT} and OXA-$48_{E185A/R186A/R206A}$ for the burst phase for the level of allosteric effect..25
Figure S16 Converting between measured and real heat signal. Solid lines represent experimental data, while dashed lines are simulations of the model for 0 – 800 mM NaCl...26
Figure S17 Numerically obtaining *c*, the conversion between moles of substrate and calories of heat generated. 26
Figure S18 Fifty independent numerical fits to the ITC curve (no chloride) consistently yield k_1'=1.40 μM^{-1} s^{-1} and k_2'=2.81 s^{-1}. 27
Figure S19 k_3' and k_{-3}' decrease with chloride. 27

Figure S20 Our mathematical model accurately fits the experimental ITC data including single-injection and double-injection assays. Solid lines represent experimental data, while dashed lines are simulations of the model....28
Figure S21 Introducing k_5 fails to capture the ITC data even for very low values from 0 to 0.5 s^{-1}, suggesting that there is no hydrolysis from the inactive intermediate leading to product....29
Figure S22 Simulated and experimental apparent reaction rates for the interface variant OXA-$48_{E185A/R186A/R206A}$. 29
Figure S23 Crystal structure of oxacillin bound in an active conformation, compared to docked structures of large substrates with bromide in inactive conformations in OXA-48_{AcK73}. 30
Figure S24 Ligplot diagrams show the hydrogen bonding and hydrophobic interactions between OXA-48_{AcK73} with the inactive acyl-intermediate conformations of (**a**) Oxacillin, (**b**) Imipenem. 31
Figure S25 The generality of halide-induced biphasic kinetics. 32
Figure S26 Thermogram curves of imipenem hydrolysis in the absence and presence of chloride by cell suspensions. 32
Figure S27 Sequence alignment of OXA-48 like carbapenemases to show R250 is strictly conserved. 34
Figure S28 Crystal structures of OXA-48_{WT} soaked with different anions. 35
Figure S29 Superimposition of the OXA-48_{AcK73}-imipenem-Br^- acyl-intermediate complex in the inactive conformation with the OXA-48_{WT}-HCO_3^- complex....36
Figure S30 HCO_3^- is a weak allosteric effector of OXA-48. 36
Figure S31 ^{19}F NMR spectra of a sample of 500 μM OXA-48_{WT} in the buffer of 500 mM NH_4F, 100 mM NaPi, pH 7.5, 10% D_2O titrated with 0-90 mM $NaHCO_3$ to show HCO_3^- ion outcompetes fluoride at lower concentrations for at least two binding sites. 37
Figure S32 ^1H NMR spectrum of N_α-(*tert*-butoxycarbonyl)-L-acetyllysine (**1**) in DMSO-d_6 (400 MHz). 47
Figure S33 ^{13}C NMR spectrum of N_α-(*tert*-butoxycarbonyl)-L-acetyllysine (**1**) in DMSO-d_6 (100 MHz). 47
Figure S34 ^1H NMR spectrum of L-acetyllysine (**2**) in D_2O (400 MHz). 48
Figure S35 ^{13}C NMR spectrum of L-acetyllysine (**2**) in D_2O (100 MHz). 48
Figure S36 High-resolution MS of L-acetyllysine (**2**). 49

Table S1 Survey of all OXA-48 structures in Protein Data Bank (PDB) (up to November 2022). The correlation of the pH, resolution, the carbamylation status of the Lys73, and any presence of halide ions.

Name		PDB	Resolution (Å)	pH	Lysine73 carbarmylated (KCX)	Any or position of halide ions
Apo		6P96	1.6	~4	no	K73, R174, R206
		3HBR	1.9	7.5	yes	none
		4S2P	1.7	7.5	yes	none
	^{19}F-labelled	6RJ7	1.73	8	yes	R206
	S70A	5HAP	1.89	8.5	yes	R206
	designed loop18	6HOO	2.38		yes	K94
	S70G	5HAQ	2.14		yes	none
	R189A	6GOA	2.55	9.5	yes	R206
		5OFT	3.20	9	yes	none
	Homocitrullinated	7LXG	2.20	5.5	Homocitrulline	R206
		6I5D	1.75	7	no	R206
Imipenem	K73A	7KH9	2.29	8.5	K73A	W157, R189, R206
		6P97	1.8	~4	no	K73, R174, R206
		6PK0	1.75	4.6	yes	R206
		5QB4	1.95	7.5	yes	R206
		6PTU	2.00	4.6	no	K73, R206
Avibactam		4S2J	2.54	6.5	no	none
	P68A	6Q5B	2.22	6.5	partially	Q91, R186, R206
		4S2K	2.1	7.5	no	none
		4WMC	2.3	7.5	no	none
		4S2N	2.0	8.5	partially	none
Other antibiotics	Meropenem	6P98	1.75	~4	no	K73, R174, R186, R206
	Ertapenem	6P99	2.25	~4	no	K73, R206
	Doripenem	6P9C	1.9	~4	no	K73, R174, R206,
	Cefotaxime	6PQI	2.05	4.6	no	K73, R206
	Faropenem	6PSG	2.13	4.6	no	R206
	Meropenem	6PT1	2.00	4.6	no	K73, R206
	Cefoxitin	6PT5	2.30	4.6	no	R206
	Ceftazidime	6Q5F	2.5	6.5-7.5	partially	Q91, W157, R186, R206
	K73A Meropenem	7KHQ	2.00		K73A	R174, R206, R214
	K73A Doripenem	6PXX	1.50	7.5-8.5	K73A	W157, R206
	L67F Ceftazidime	7ASS	1.91	9	yes	R206, R214, R250
	Ertapenem	6ZRJ	1.94	7.5	partially	R206
	Meropenem	6ZRP	1.74	7.5	partially	R206
Other ligands		7AW5	1.65	7.5	yes	R206
		7AUX	2.05	7.5	yes	R206
		6V1O	1.80	8.5	yes	R206
		7JHQ	2.00	8.5	yes	none
		6XQR	2.20	8.5	yes	R206
		7K5V	2.80 Å	8.5	yes	R206
		7L8O	2.70	8.5	yes	R186, Q193, R206, K208
		7R6Z	2.10	8.5	yes	S40, R206
		5DTS	1.94	7.5	yes	R206
		5DTK	1.60	7.5	yes	R206
		5DTT	2.10	7.5	yes	R206
		5DVA	2.50	7.5	yes	R206

		6UVK	2.20	8	yes	N110, R206
		7DML	1.94	7.5	yes	none
		6ZXI	1.85	7.5	no	R206
		5QA4	1.95	7.5	yes	R174, R206
		5QA5	1.95	7.5	yes	Y177, R206
		5QA6	1.95	7.5	yes	R206
		5QA7	1.82	7.5	yes	R206
		5QA8	2.50	7.5	yes	R206
		5QA9	1.90	7.5	yes	R206
		5QAA	1.95	7.5	yes	R206
		5QAB	2.15	7.5	yes	R206
		5QAC	2.00	7.5	yes	R206
		5QAD	1.75	7.5	yes	R206
		5QAE	2.10	7.5	yes	R206
		5QAF	2.15	7.5	yes	R206
		5QAG	2.30	7.5	yes	R206
		5QAH	1.95	7.5	yes	R206
		5QAI	1.90	7.5	yes	R206
		5QAJ	2.00	7.5	yes	R206
		5QAK	1.90	7.5	yes	R206
		5QAL	1.95	7.5	yes	R206
		5QAM	1.87	7.5	yes	R206
		5QAN	2.30	7.5	yes	R206
		5QAO	2.00	7.5	yes	R206
		5QAP	1.79	7.5	yes	R206
		5QAQ	2.40	7.5	yes	R206
		5QAR	2.10	7.5	yes	R206
		5QAS	1.90	7.5	yes	none
		5QAT	1.90	7.5	yes	R206
		5QAU	1.75	7.5	yes	R206
		5QAV	1.72	7.5	yes	R206
		5QAW	2.20	7.5	yes	R206
		5QAX	2.31	7.5	yes	R206
		5QAY	1.70	7.5	yes	R206
		5QAZ	2.20	7.5	yes	R206
		5QB0	1.95	7.5	yes	R206
		5QB1	1.80	7.5	yes	R206
		5QB2	1.75	7.5	yes	R206
		5QB3	2.00	7.5	yes	R206
		5FAQ	1.96	7.5	no	R206
		5FAS	1.74	7.5	no	R206
		5FAT	2.09	7.5	no	R206

Table S2 IC_{50} Values of OXA-48 for all four halide ions.

	**IC_{50} (mM)[a]**
**$F^–$**	ND
**$Cl^–$**	24 ± 2
**$Br^–$**	2.9 ± 0.3
**$I^–$**	2.25 ± 0.03

[a] Determined with the steady-state rate v_s.

Table S3 Data collection and refinement statistics

	OXA-48$_{WT}$ apo	OXA-48$_{WT}$-Br^-	OXA-48$_{WT}$-I^-	OXA-48$_{E185A/R186A/R206A}$-Br^-	OXA-48$_{R250A}$-Br^-
**PDB**	**7PEH**	**7O5T**	**7NRJ**	**7PEI**	**7PGO**
**Data collection**					
Wavelength (Å)	0.815342	0.918374	0.979150	0.979150	0.918395
Space group	P 21 21 21	P 21 21 21	P 1 21 1	P 62	P 21 21 21
Cell dimensions					
a, *b*, *c* (Å)	83.00, 106.98, 124.61	82.57, 107.26, 124.62	46.76, 126.86, 111.10	146.19, 146.19, 54.89	84.55, 108.20, 124.61
α, β, γ (°)	90.00, 90.00, 90.00	90.00, 90.00, 90.00	90.00, 98.35, 90.00	90.00, 90.00, 120.00	90.00, 90.00, 90.00
Resolution (Å)	53.84-1.92 (1.96-1.92)	81.30-1.81 (1.84-1.81)	19.78-1.67 (1.70-1.67)	19.69-1.90 (1.94-1.90)	58.75-1.85 (1.88-1.85)
R_{sym} or R_{merge} (within I+/I-)	0.313 (2.917)	0.193 (2.259)	0.088 (1.847)	0.125 (4.749)	0.381 (3.815)
R_{sym} or R_{merge} (all I+ and I-)	0.324 (3.030)	0.199 (2.347)	0.095 (1.885)	0.127 (4.858)	0.388 (3.890)
I / σI	5.8 (1.0)	7.4 (1.0)	11.0 (1.0)	16.1 (1.0)	6.6 (1.1)
Completeness (%)	99.9 (99.8)	99.9 (99.7)	99.9 (99.9)	99.9 (100.0)	100.0 (99.8)
Multiplicity	12.0 (11.4)	13.3 (12.6)	6.8 (6.9)	20.6 (20.5)	23.2 (23.7)
**Refinement**					
Resolution (Å)	53.90-1.92	81.43-1.81	19.74-1.67	19.70-1.90	58.82-1.85
No. reflections all / free	85147 / 4389	101119 / 5098	147791 / 7450	52978 / 2657	97955 / 5022
R_{work} / R_{free}	0.218 / 0.250	0.187 / 0.261	0.166 / 0.197	0.177 / 0.211	0.205 / 0.234
No. atoms					
Protein	7907	8009	7958	3944	7978
Ligand / ion	83 /	54 / 7	119 / 13	73 /	72 / 3
Water	455	709	923	258	902
B-factors					
Protein	25.46	28.86	24.98	40.39	21.76
Ligand / ion	28.77 /	27.45 / 39.68	31.43 / 34.46	42.44 /	22.48 / 28.92
Water	26.63	36.28	37.83	46.82	28.28
R.m.s. deviations					
Bond lengths (Å)	0.0075	0.0099	0.0098	0.0089	0.0088
Bond angles (°)	1.415	1.490	1.602	1.583	1.435

*Values in parentheses are for highest-resolution shell.

	OXA-48$_{AcK}$-imipenem-Br^-	OXA-48$_{AcK}$-imipenem	OXA-48$_{AcK}$-oxaciline	OXA-48$_{WT}$-imipenem-Br^-	OXA-48$_{WT}$-HCO_3^-
**PDB**	**7Q14**	**7PFN**	**7PSE**	**8QNZ**	**7O9N**
**Data collection**					
Wavelength (Å)	0.976269	0.976292	0.976270	0.918381	0.976254
Space group	P 1 21 1	P 2 21 21	P 2 21 21	P 21 21 21	P 21 21 21
Cell dimensions					
a, *b*, *c* (Å)	63.40, 161.93, 107.78	44.80, 105.00, 124.75	44.83, 105.48, 124.96	81.31, 105.97, 124.68	81.81, 106.40, 124.71
α, β, γ (°)	90.00, 90.51, 90.00	90.00, 90.00, 90.00	90.00, 90.00, 90.000	90.00, 90.00, 90.00	90.00, 90.00, 90.000
Resolution (Å)	80.97-2.15 (2.19-2.15)	62.45-1.80 (1.84-1.80)	53.76-2.32 (2.40-2.32)	80.74-1.53 (1.56-1.53)	81.81-1.97 (2.01-1.97)
R_{sym} or $R_{merge\ (within\ I+/I-)}$	0.165 (1.628)	0.172 (2.448)	0.262 (2.266)	0.132 (2.755)	0.340 (2.727)
R_{sym} or $R_{merge\ (all\ I+\ and\ I-)}$	0.181 (1.747)	0.179 (2.545)	0.276 (2.421)	0.139 (2.854)	0.351 (2.820)
I / σI	7.3 (1.1)	9.9 (1.1)	7.0 (1.0)	12.0 (1.1)	6.0 (1.0)
Completeness (%)	99.6 (99.1)	100.0 (100.0)	99.3 (95.3)	98.3 (96.9)	100.0 (100.0)
Multiplicity	6.7 (6.7)	11.4 (11.5)	10.9 (8.8)	13.6 (14.1)	13.2 (13.6)
**Refinement**					
Resolution (Å)	81.10-2.15	62.45-1.80	53.81-2.32	62.42-1.53	68.50-1.97
No. reflections all / free	117142 / 5895	55487 / 2745	26280 / 1266	159336 / 7990	77539 / 3748
R_{work} / R_{free}	0.210 / 0.250	0.178 / 0.214	0.235 / 0.289	0.147 / 0.199	0.195 / 0.286
No. atoms					
Protein	15708	3976	3934	8113	8005
Ligand / ion	372 / 4	94 /	100 / 1	124 / 7	68 /
Water	518	365	64	1020	616
B-factors					
Protein	45.90	26.51	50.37	21.46	27.15
Ligand / ion	42.48 / 37.13	32.94 /	41.91 / 27.56	28.77 / 36.17	24.28 /
Water	35.38	33.15	34.31	32.38	32.01
R.m.s. deviations					
Bond lengths (Å)	0.0081	0.0096	0.0075	0.0091	0.0090
Bond angles (°)	1.464	1.539	1.529	1.480	1.466

*Values in parentheses are for highest-resolution shell.

	OXA-48$_{WT}$-imipenem *product*	OXA-48$_{WT}$-imipenem *intermediate*
**PDB**	**7PEP**	**7PSF**
**Data collection**		
Wavelength (Å)	0.918381	0.918380
Space group	P 21 21 21	P 21 21 21
Cell dimensions		
a, *b*, *c* (Å)	83.95, 107.93, 124.42	83.42, 107.60, 124.59
α, β, γ (°)	90.00, 90.00, 90.00	90.00, 90.00, 90.00
Resolution (Å)	69.59-1.70 (1.73-1.70)	49.39-2.10 (2.15-2.10)
R_{sym} or $R_{merge\ (within\ I+/I-)}$	0.170 (2.436)	0.282 (3.195)
R_{sym} or $R_{merge\ (all\ I+\ and\ I-)}$	0.177 (2.535)	0.294 (3.331)
$I / \sigma I$	9.8 (1.0)	8.2 (1.0)
Completeness (%)	100.0 (99.7)	99.9 (99.9)
Multiplicity	13.3 (12.7)	13.6 (14.0)
**Refinement**		
Resolution (Å)	69.69-1.70	49.44-2.10
No. reflections all / free	124488 / 6286	66012 / 3258
R_{work} / R_{free}	0.199 / 0.231	0.241 / 0.278
No. atoms		
Protein	8033	7932
Ligand / ion	213 / 2	106 /
Water	881	231
B-factors		
Protein	24.58	39.36
Ligand / ion	33.47 /43.84	39.56
Water	32.61	32.64
R.m.s. deviations		
Bond lengths (Å)	0.0089	0.0089
Bond angles (°)	1.518	1.523

*Values in parentheses are for highest-resolution shell

Table S4 T_m value of OXA-48$_{WT}$ and OXA-48$_{E185A/R186A/R206A}$ with chloride.

	[Cl⁻] (mM)	Tm (°C)			Mean ± SD
OXA-48$_{WT}$	0	54.91	55.06	55.24	55.1 ± 0.2
	40	57.45	58.30	58.33	58.0 ± 0.4
	100	58.10	58.69	58.58	58.5 ± 0.3
OXA-48$_{E185A/R186A/R206A}$	0	44.86	44.16	44.33	44.5 ± 0.4
	40	44.53	44.26	44.27	44.4 ± 0.1
	100	45.15	44.45	44.62	44.7 ± 0.4

Table S5 Sequence alignment for OXAs that have been reported to display biphasic kinetics.

	Enzyme	Type	Kinetics (Substrate)		Inhibited by halide	Sequence (Align to OXA-48)			
						70-73	250	214	GenBank ID
1	OXA-48	CHDL	biphasic	imipenem<br>...	Y	STFK	R	R	>API82700.1 OXA-48 (plasmid) [*Klebsiella pneumoniae*]
2	OXA-22	Narrow spectrum	biphasic	cephalosporins<br>penicillins<br>...	Unknown	STFK	R	-	>QCO89828.1 OXA-2 (plasmid) [*Escherichia coli*]
3	OXA-103	Narrow spectrum	biphasic	ampicillin<br>carbenicillin<br>oxacillin<br>cloxacillin<br>cephaloridine	Y	STFK	R	-	>ACI28891.1 OXA-10 (plasmid) [*Pseudomonas aeruginosa*]
4	OXA-144	Expanded spectrum	biphasic	oxacillin<br>penicillin G<br>cephaloridine<br>carbenicillin	Y	STFK	R	-	>WP_064056056.1 OXA-10 family oxacillin-hydrolyzing class D extended-spectrum β-lactamase OXA-14 [*Pseudomonas aeruginosa*]
5	OXA-165	Expanded spectrum	biphasic	penicillin G<br>ampicillin<br>carbenicillin<br>oxacillin<br>cloxacillin<br>cephaloridine<br>cefotaxime	Unknown	STFK	R	-	>AAB97924.1 β-lactamase OXA-16, partial [*Pseudomonas aeruginosa*]
6	OXA-276	CHDL	biphasic	oxacillin	Y	STFK	R	-	>AAG35609.2 β-lactamase OXA-27 [*Acinetobacter baumannii*]
7	OXA-507	Narrow spectrum	biphasic	ampicillin<br>cefsulodin<br>piperacillin	Y	ST**Y**K	R	-	>WP_225025027.1 OXA-50 family oxacillin-hydrolyzing class D β-lactamase [*Pseudomonas aeruginosa*]

8	OXA-24	CHDL			Y	STFK	R	-	>ACV72170.1 OXA-24 class D β-lactamase (plasmid) [*Acinetobacter baumannii*]
9	OXA-25	CHDL			Y	STFK	R	-	>AAG35607.1 beta-lactamase OXA-25 [*Acinetobacter baumannii*]
10	OXA-26	CHDL			Y	STFK	R	-	>AAG35608.1 β-lactamase OXA-26 [*Acinetobacter baumannii*]
11	OXA-40	CHDL			N	STFK	R	-	>ADB28893.1 class D β-lactamase OXA-40 [*Acinetobacter baumannii*]
12	OXA-58	CHDL			Y	STFK	R	-	>AAW57529.1 OXA-58 [*Acinetobacter baumannii*]
13	OXA-163	Expanded spectrum			Y	STFK	R	-	>ADY06444.1 β-lactamase OXA-163 [*Enterobacter cloacae*]

CHDL - Carbapenem-hydrolyzing class D β-lactamase

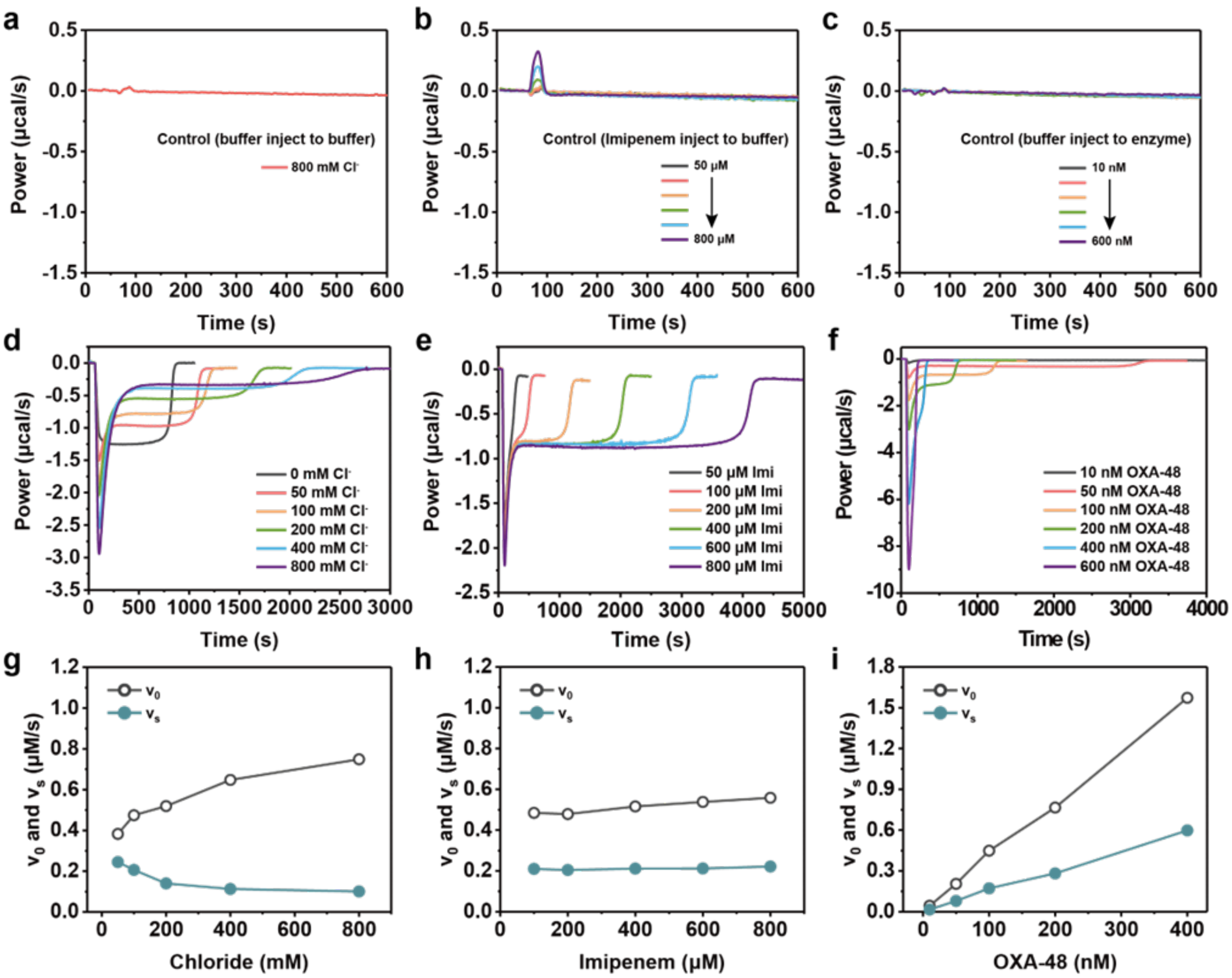

Figure S1 Effect of enzyme, substrate or chloride concentrations on biphasic kinetics in ITC assays. (**a**) Buffer (50 mM NaPi, 800 mM NaCl, 1 mM $NaHCO_3$, pH 7.5) was injected into the same buffer as control; (**b**) Different concentrations of imipenem in buffer 50 mM NaPi, pH 7.5, 100 mM NaCl, 1 mM $NaHCO_3$ was injected into the same buffer as control; (**c**) Buffer (50 mM NaPi, 100 mM NaCl, 1 mM $NaHCO_3$, pH 7.5) was injected into different concentrations of OXA-48 in the same buffer as control; (**d**) Effect of chloride concentrations on biphasic kinetics. 200 μM substrate was injected into 100 nM OXA-48_{WT} in the matched buffer (0-800 mM NaCl, 50 mM NaPi, pH 7.5, 1 mM $NaHCO_3$); (**e**) Effect of substrate concentrations on biphasic kinetics. 50-800 μM imipenem in the buffer of 50 mM NaPi, pH 7.5, 1 mM $NaHCO_3$, and 100 mM NaCl was injected into 100 nM enzyme in the matched buffer; (**f**) Effect of OXA-48_{WT} concentrations on biphasic kinetics. 10 - 600 nM OXA-48_{WT} in the buffer of 50 mM NaPi, pH 7.5, 1 mM $NaHCO_3$, and 100 mM NaCl was injected into 200 μM substrate in the matched buffer. Based on the ITC curves in (**d-f**), v_0 and v_s for (**g**) different [Cl^-], (**h**) [substrate] and (**i**) [OXA-48_{WT}] were plotted to show v_0/v_s is only [Cl^-]-dependent.

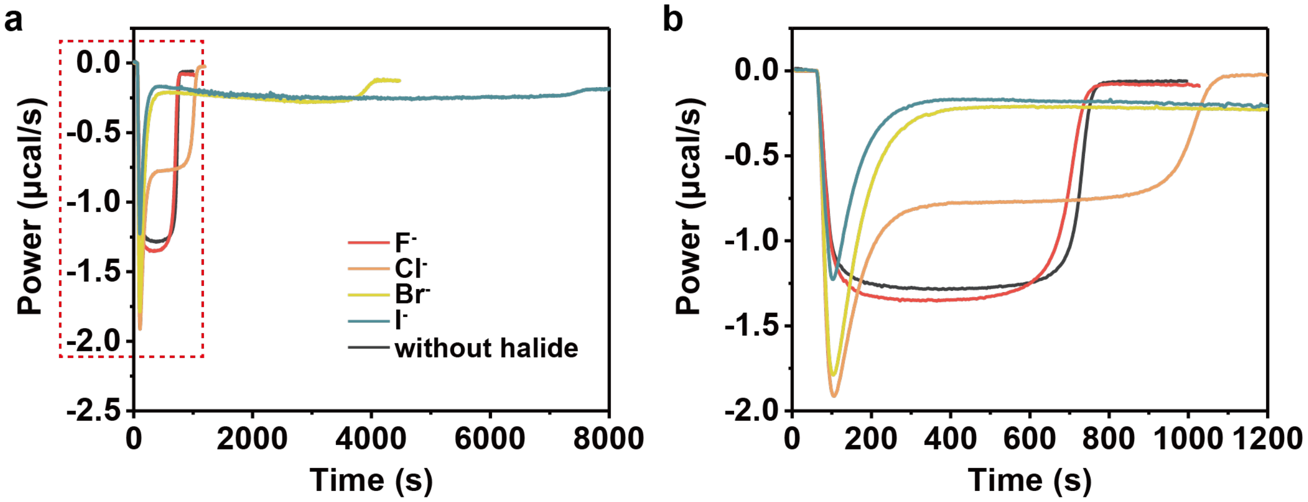

Figure S2 Effect of four halide ions at 100 mM on hydrolysis of imipenem by OXA-48.
(**a**) 10 μL imipenem solution was injected into 210 μL of OXA-48_{WT} (final concentration: 200 μM imipenem, 100 nM OXA-48_{WT}), both prepared in buffer of 50 mM NaPi, pH 7.5, 1 mM $NaHCO_3$, with 100 mM corresponding halide ions). (**b**) is a closeup of (a) from 0 to 1200 s.

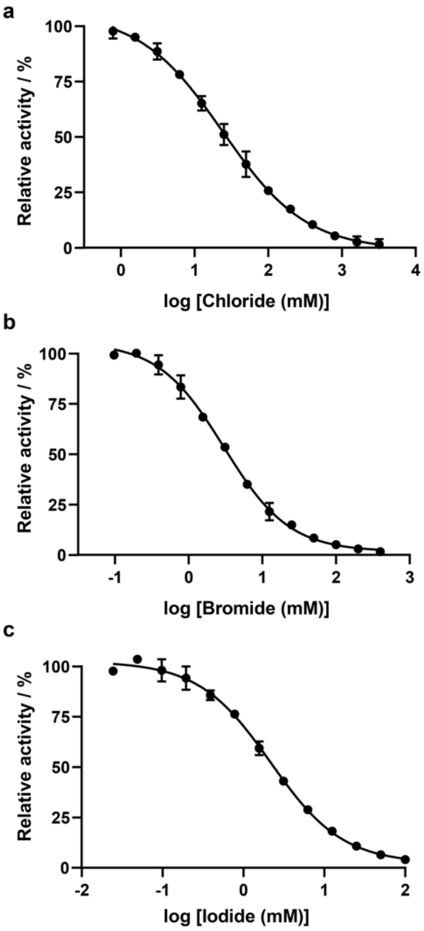

Figure S3 IC_{50} plots of (**a**) chloride, (**b**) bromide and (**c**) iodide to reflect the inhibiting effect during imipenem hydrolysis by OXA-48_{WT}.

All 1 mL reactions were set up using 200 μM imipenem as the substrate in the buffer of 50 mM NaPi, pH 7.5, with various concentrations of halide ions by adding NaI, NaBr, NaCl, or NaF. Reactions were initiated by adding OXA-48$_{WT}$ to a final concentration of 100 nM and the absorbance of imipenem at 300 nm were recorded continuously. All curves are fitted with nonlinear regression analysis.

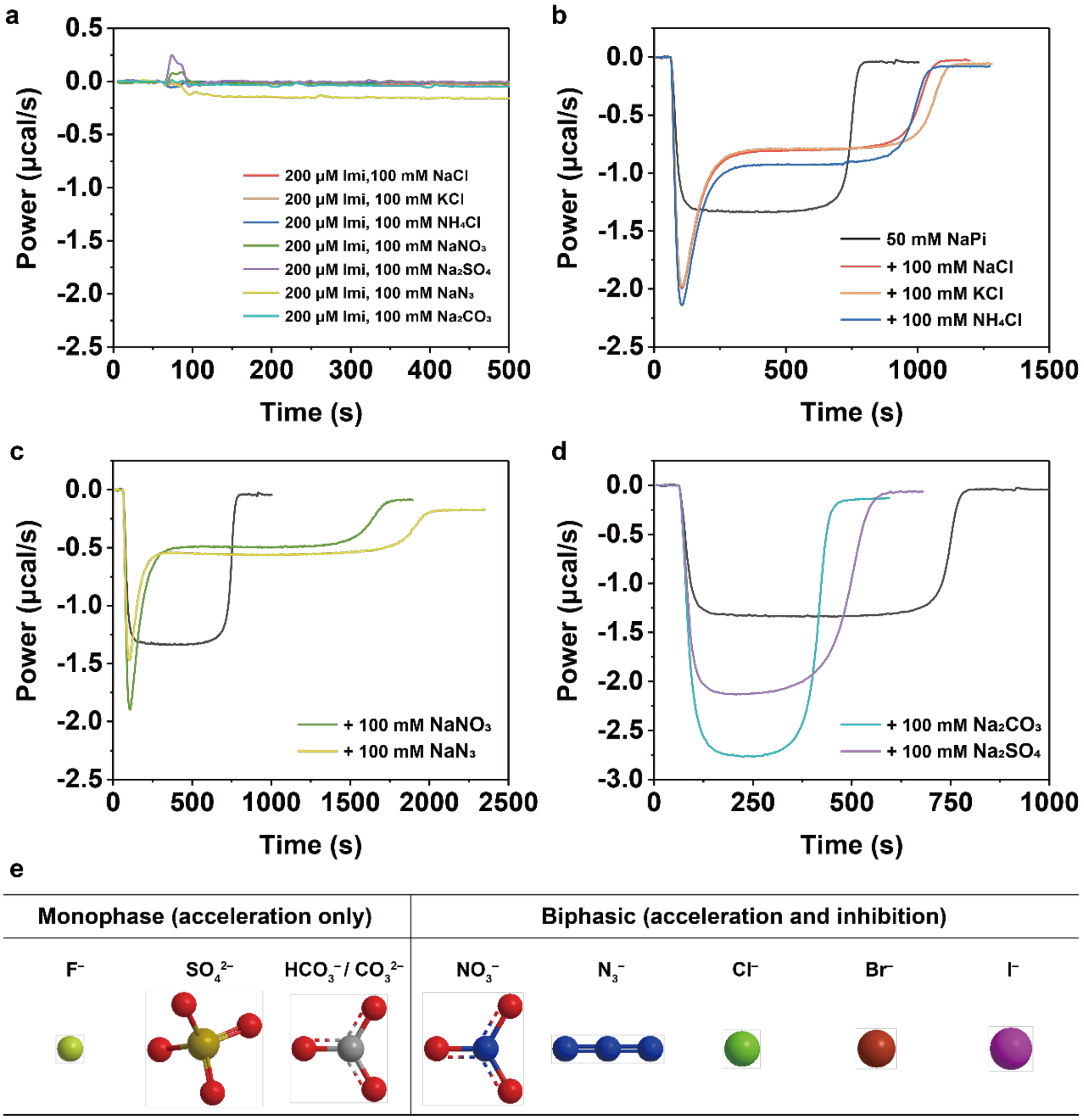

Figure S4 The effect of ITC kinetics curves induced by various common cations and anions.

(**a**) Imipenem solution in the buffer with various salts was injected into the sample cell filled with matched buffer. The control ITC heat profiles show there is only negligible heat detected when there is no OXA-48$_{WT}$. (**b**) Effect of different cations on the kinetic curves. 200 μM imipenem was hydrolyzed by 100 nM OXA-48$_{WT}$ with the supplement of 100 mM chloride salts of sodium, potassium, and ammonium. The black curve is the control without any chloride salt. (**c, d**) Effect of various anions on the kinetic rates. 200 μM imipenem was hydrolyzed by 100 nM OXA-48$_{WT}$ with the supplement of 100 mM of various sodium salts. All buffers used for ITC assays are 50 mM NaPi pH 7.5 with 1 mM $NaHCO_3$.

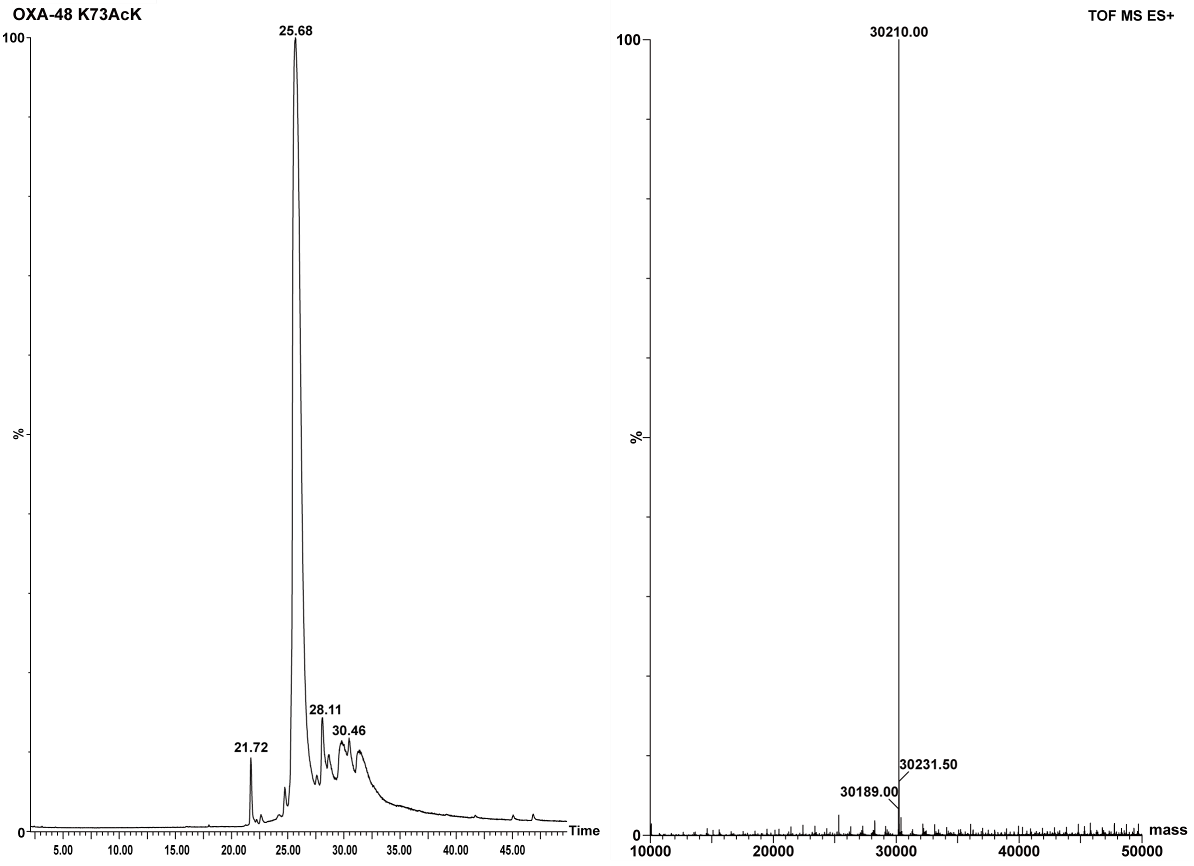

Figure S5 Mass spectrometry of OXA-48_{AcK73}.

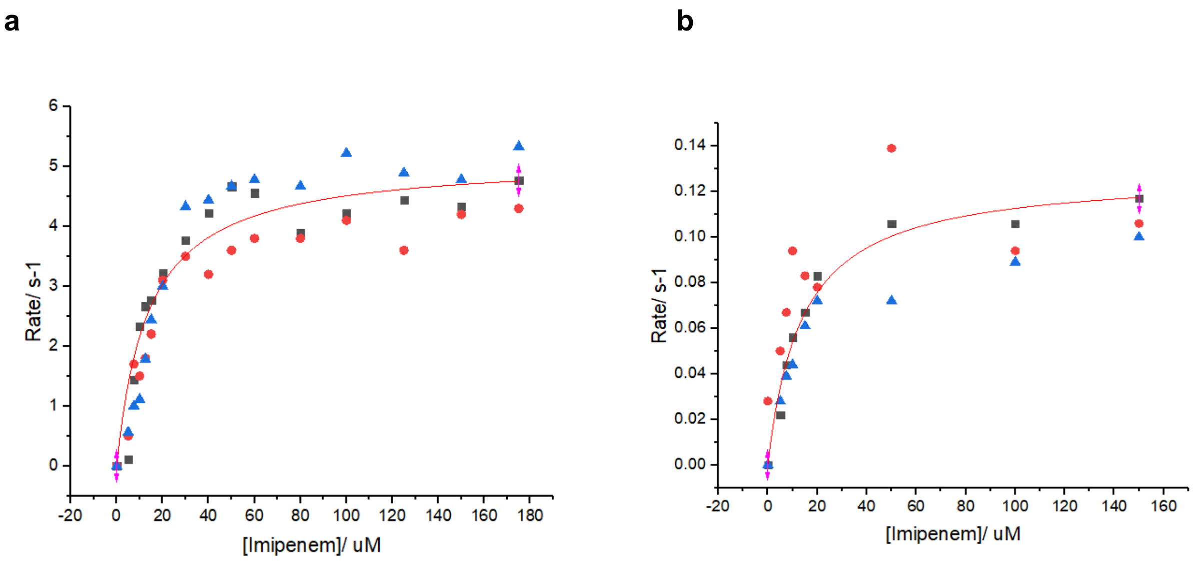

Figure S6 Michaelis-Menten kinetics were measured in triplicate for OXA-48_{AcK73} variant (**b**) to compare with OXA-48_{WT} (**a**) by UV-vis method at 25 °C in the buffer of 100 mM NaPi, 50 mM $NaHCO_3$, pH 7.5, with strictly no chloride.

For OXA-48_{WT}, K_M = 13.79 μM, k_{cat} = 5.13 s^{-1}, and k_{cat}/K_M = 3.72 x 10^5 $M^{-1}s^{-1}$. For OXA-48_{AcK73}, K_M = 13.89 μM and k_{cat} = 0.13 s^{-1}, and k_{cat}/K_M = 9.36 x 10^3 $M^{-1}s^{-1}$.

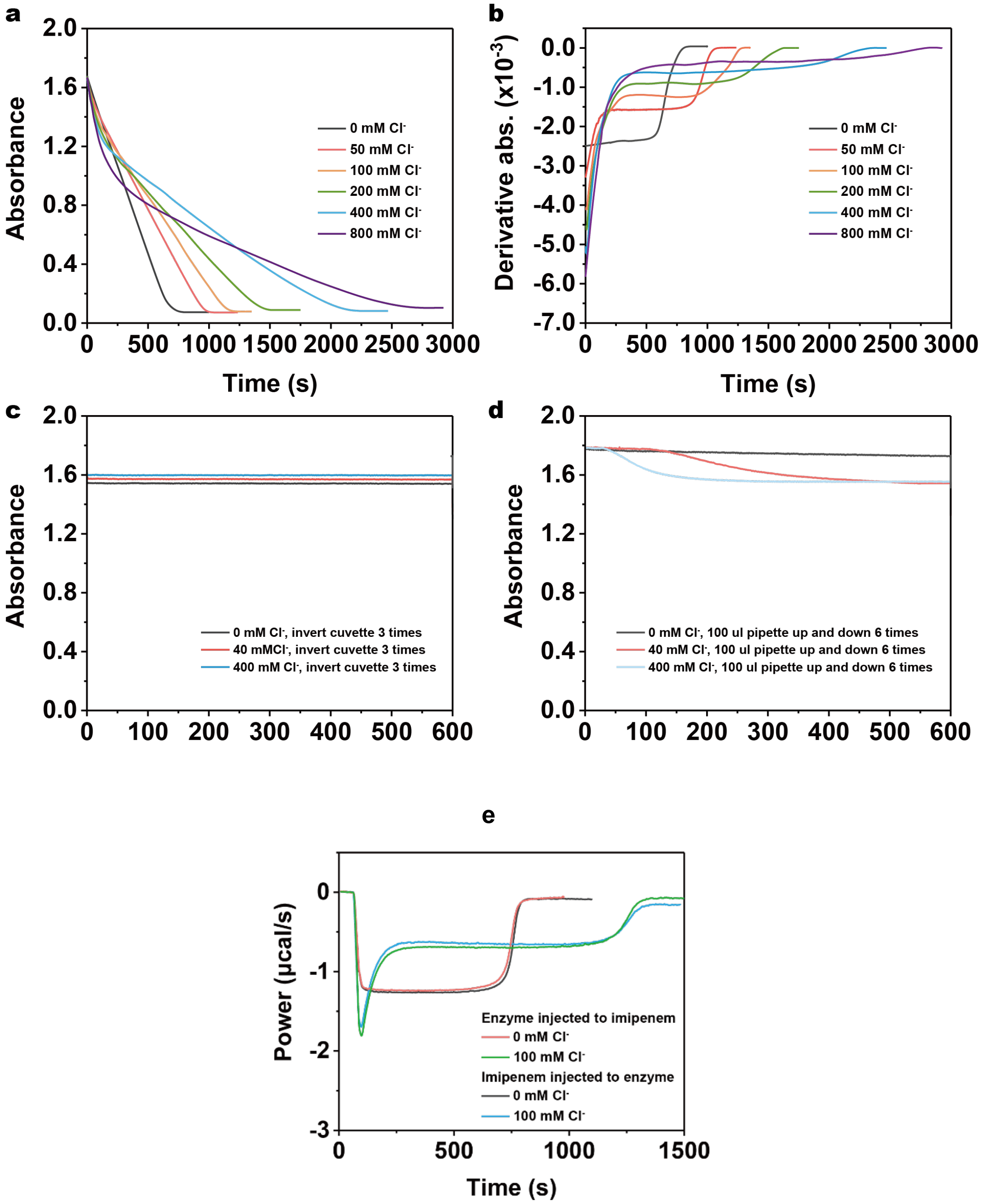
a
Absorbance
Time (s)
0 mM Cl-
50 mM Cl-
100 mM Cl-
200 mM Cl-
400 mM Cl-
800 mM Cl-
b
Derivative abs. (x10-3)
Time (s)
0 mM Cl-
50 mM Cl-
100 mM Cl-
200 mM Cl-
400 mM Cl-
800 mM Cl-
c
Absorbance
0 mM Cl-, invert cuvette 3 times
40 mMCl-, invert cuvette 3 times
400 mM Cl-, invert cuvette 3 times
d
Absorbance
0 mM Cl-, 100 ul pipette up and down 6 times
40 mM Cl-, 100 ul pipette up and down 6 times
400 mM Cl-, 100 ul pipette up and down 6 times
e
Power (µcal/s)
Time (s)
Enzyme injected to imipenem
0 mM Cl-
100 mM Cl-
Imipenem injected to enzyme
0 mM Cl-
100 mM Cl-

f

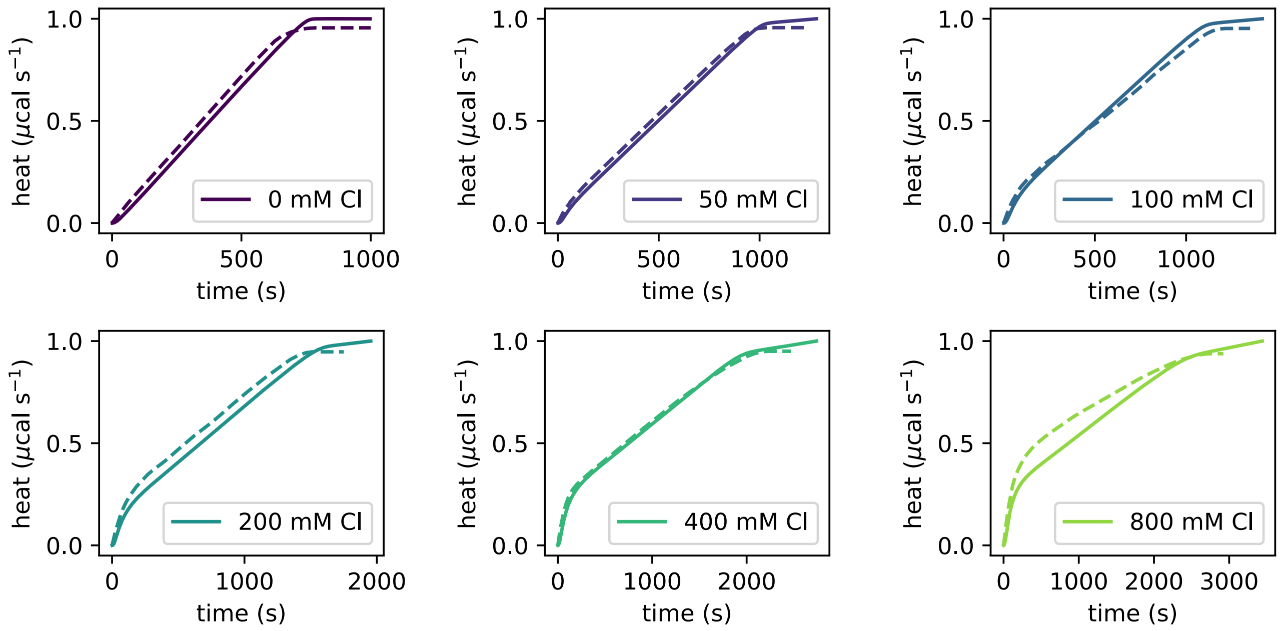

Figure S7 UV-vis for monitoring imipenem hydrolyzed by OXA-48 to show it is compatible with ITC, and comparison of the ITC heat profile to show the fast-mixing deadtime is negligible when the order of injection was changed.

(**a**) UV-vis spectra of OXA-48 hydrolyzing imipenem in the buffer with a series of chloride ion concentrations. Final concentration: 200 μM imipenem, 100 nM OXA-48 in 50 mM NaPi, pH 7.5, 1 mM $NaHCO_3$ with 0-800 mM chloride. (**b**) The first derivative d*A*/d*t* of the absorbance in (a) shows the rate of change of absorbance, which is compatible with the rate of change of heat measured by ITC. (**c**, **d**) Comparison of mixing methods of “inverting cuvette” with “pipetting” for the UV-vis assay in the presence of imipenem and chloride, to show the UV-vis absorbance spectra is highly sensitive to different mixing techniques. The absorbance of imipenem was monitored at 300 nm. Final concentration: 200 μM imipenem in 50 mM NaPi, pH 7.5 buffer supplemented with different chloride concentrations. All assays were carried out in a total volume of 1 mL with 100 nM OXA-48, 200 μM imipenem and 0-800 mM chloride. The decrease of imipenem absorbance was monitored continuously at 300 nm. Data were plotted using Origin 2017 Software. (**e**) Comparison of the order of injection in ITC assays is not affecting reproducibility. 200 μM imipenem solution and 100 nM OXA-48_{WT} solution are fast mixed when one is injected into the other in ITC in the matched buffer of 50 mM NaPi, pH 7.5, 1 mM $NaHCO_3$ buffer with 0 or 100 mM chloride. (**f**) Overlay of the integration of ITC in Figure S1(d) compared with the normalized absorbance in (a) shows the integrated heat change measured by ITC is compatible with the change of absorbance, both are measured using the following final concentrations in the reaction: 200 μM imipenem, 100 nM OXA-48 in 50 mM NaPi, pH 7.5, 1 mM $NaHCO_3$ with 0-800 mM chloride. Solid lines are ITC data, and dashed lines are UV-vis data.

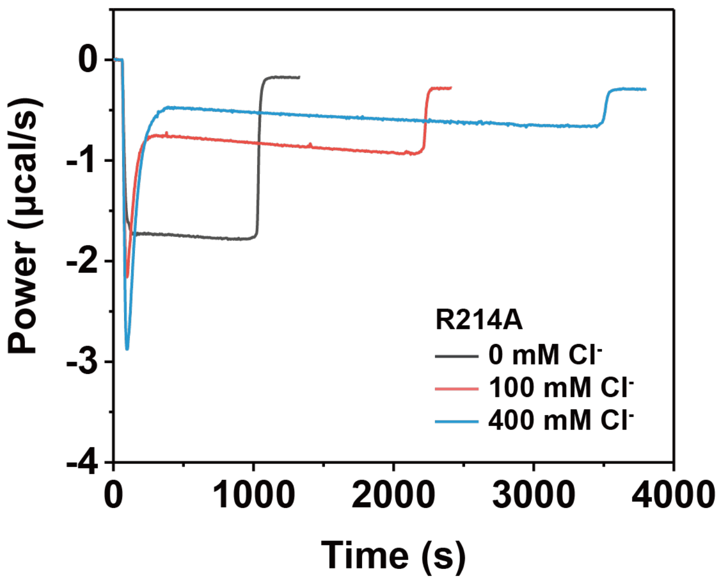

Figure S8 ITC kinetic curves of 400 μM imipenem hydrolysis by 3 μM OXA-48$_{R214A}$ variant with 0 mM, 100 mM or 400 mM chloride ion in the buffer of 50 mM NaPi, pH 7.5, and 1 mM $NaHCO_3$.

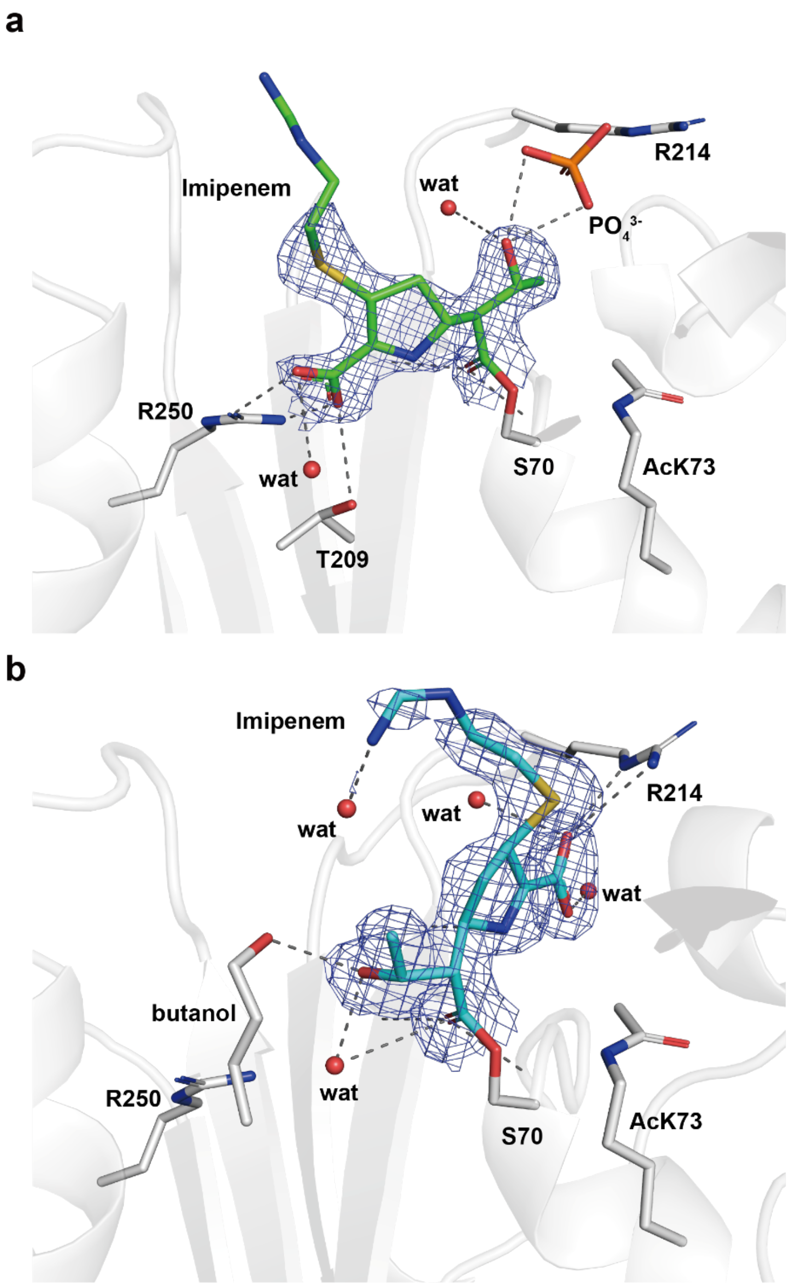

Figure S9 The crystal structure of chloride-free OXA-48AcK-imipenem acyl-intermediate complex. (**a**) The active conformation and (**b**) the inactive conformation. Unbiased $2F_o-F_c$ electron density map of imipenem is shown as blue mesh, contoured at 1σ (0.3116 $e^-/Å^3$).

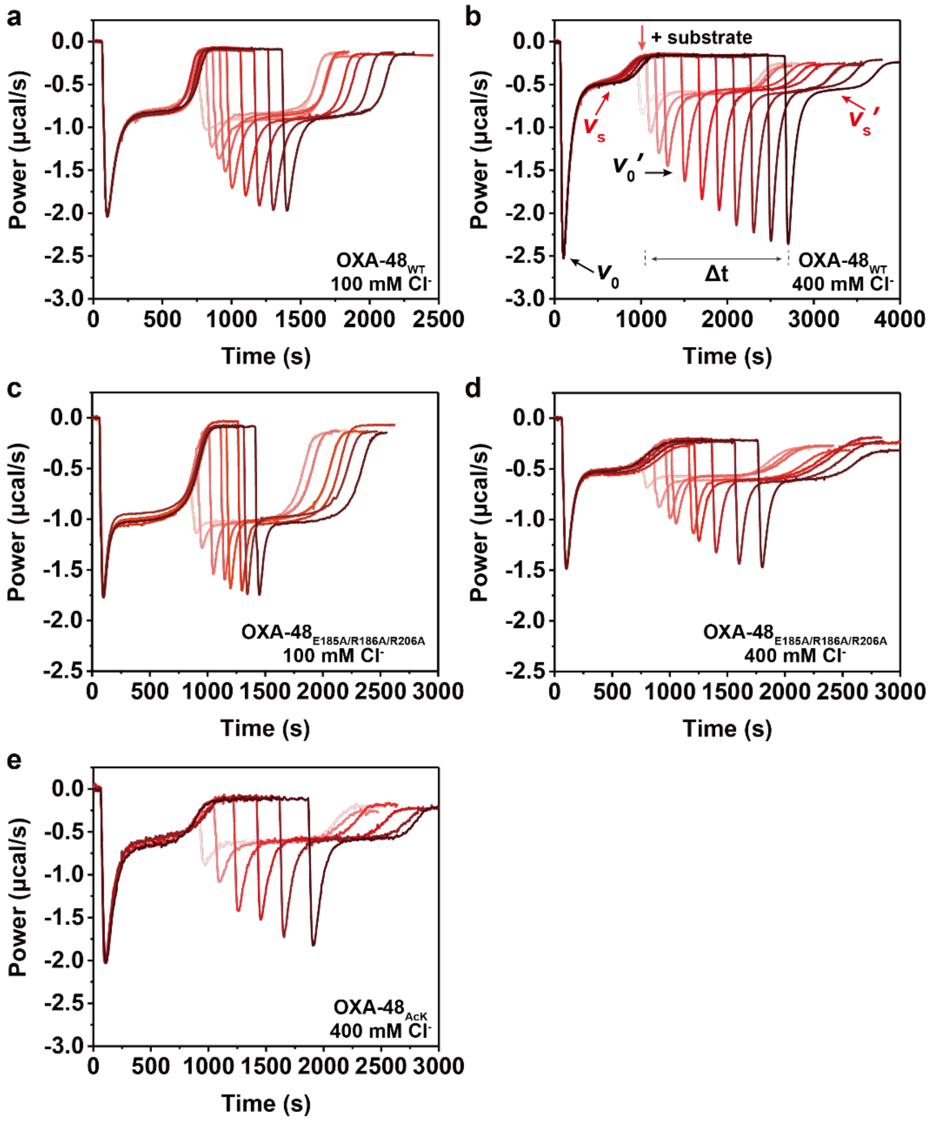

Figure S10 Overlays of kinetics curves for titrating different time intervals between two imipenem injections into OXA-48 in order to show the time required for the initial activity recovery before the 2nd injection.

(**a**) 180 μM imipenem x2 was hydrolyzed by 100 nM OXA-48$_{WT}$ in the buffer of 50 mM NaPi, pH 7.5, 1 mM $NaHCO_3$, supplemented with 100 mM Cl^-. (**b**) 140 μM imipenem x2 was hydrolyzed by 100 nM OXA-48$_{WT}$ in the buffer of 50 mM NaPi, pH 7.5, 1 mM $NaHCO_3$, supplemented with 400 mM Cl^-. (**c**) 200 μM imipenem x2 was hydrolyzed by 100 nM interface variant OXA-48$_{E185A/R186A/R206A}$ in the buffer of 50 mM NaPi, pH 7.5, 1 mM $NaHCO_3$, supplemented with 100 mM Cl^-. (**d**) 80 μM imipenem x2 was hydrolyzed by 100 nM interface variant OXA-48$_{E185A/R186A/R206A}$ in the buffer of 50 mM NaPi, pH 7.5, 1 mM $NaHCO_3$, supplemented with 400 mM Cl^-. (**e**) 150 μM imipenem x2 was hydrolyzed by 6.5 μM OXA-48$_{AcK}$ in the buffer of 50 mM NaPi, pH 7.5, containing 1 mM $NaHCO_3$, supplemented with 400 mM Cl^-. All concentrations are the final concentrations used in the reaction.

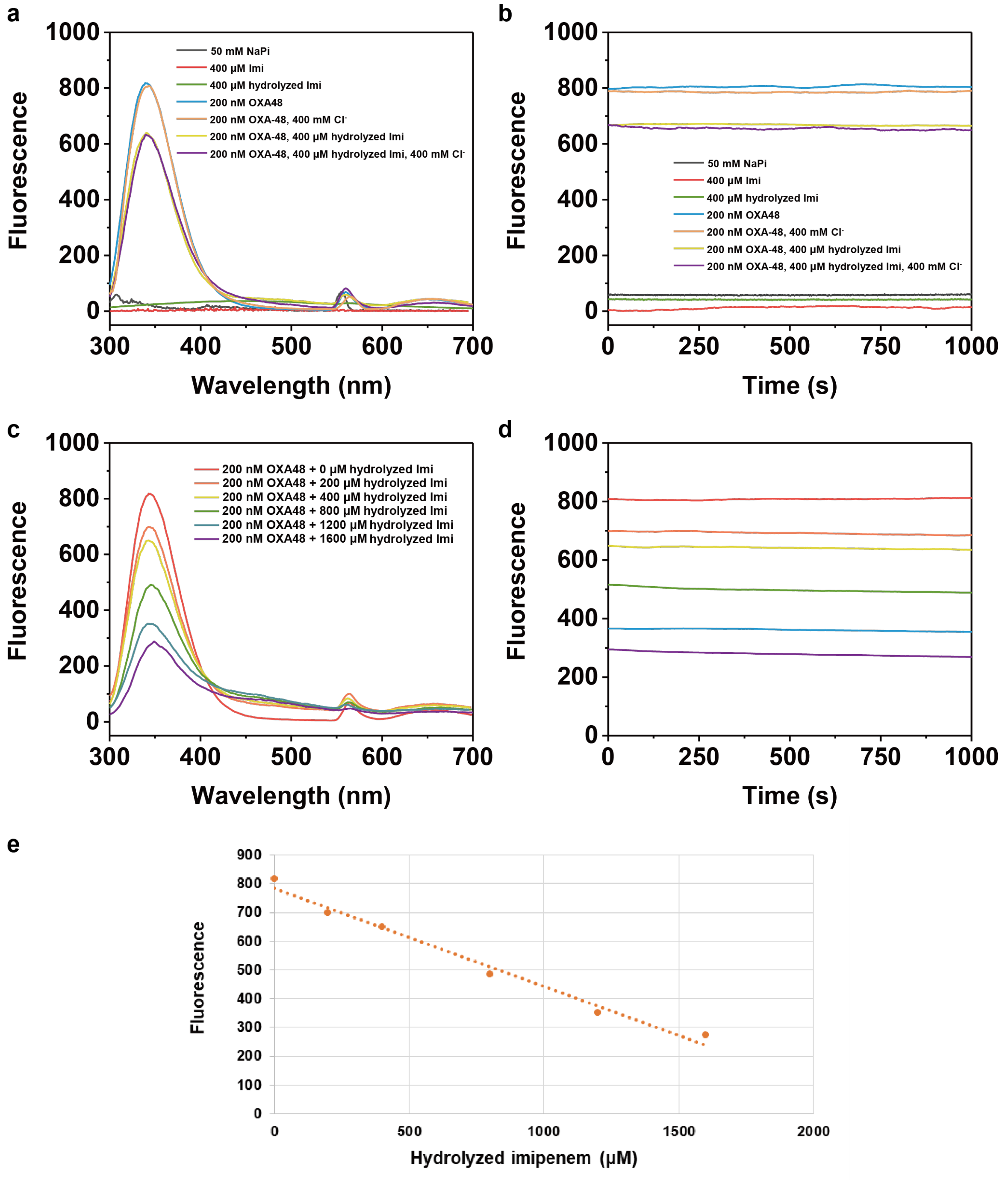

Figure S11 The intrinsic tryptophan fluorescence spectra of OXA-48$_{WT}$ to show the intensity at λ_{em} = 340 nm is only affected by the concentration of hydrolyzed imipenem product, but not by other components in the assay.

(**a, b**) The intrinsic tryptophan fluorescence spectra (λ_{ex} = 280 nm, λ_{em} = 300-700 nm) of 200 nM OXA-48$_{WT}$ with the addition of 400 μM hydrolyzed imipenem product and other components in the buffer to demonstrate the fluorescence of the OXA-48 is not affected by other components in the assay but only the product of imipenem. Fluorescence is stable within the time frame of the activity assay. (**c, d**) The intrinsic fluorescence spectra of 200 nM OXA-48$_{WT}$ mixed with 0-1600 μM hydrolyzed imipenem at λ_{ex} = 280 nm, λ_{em} = 300-700 nm, showing the product quantitatively reduces the intrinsic fluorescence intensity. (**e**) Linear fitting plot of fluorescence from 200 nM OXA-48$_{WT}$ protein versus 0-

1600 μM hydrolyzed imipenem. All the reactions were performed in 2 mL buffer of 50 mM NaPi, pH 7.5, 1 mM $NaHCO_3$, with 0 or 400 mM chloride at 25 °C.

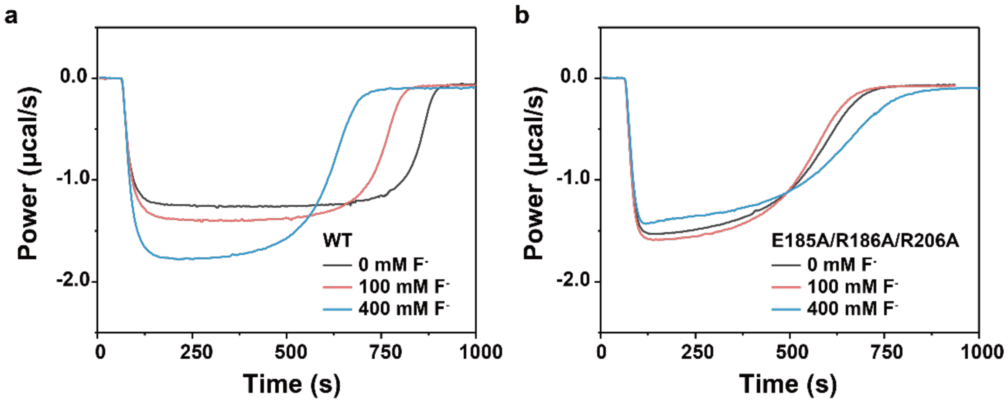

Figure S12 Kinetic curves for imipenem hydrolysis by OXA-48_{WT} with 0 mM, 100 mM, and 400 mM F^-. F^- only affects the hydrolytic rate through the allosteric effect, using the surface variant OXA-$48_{E185A/R186A/R206A}$ as a control.

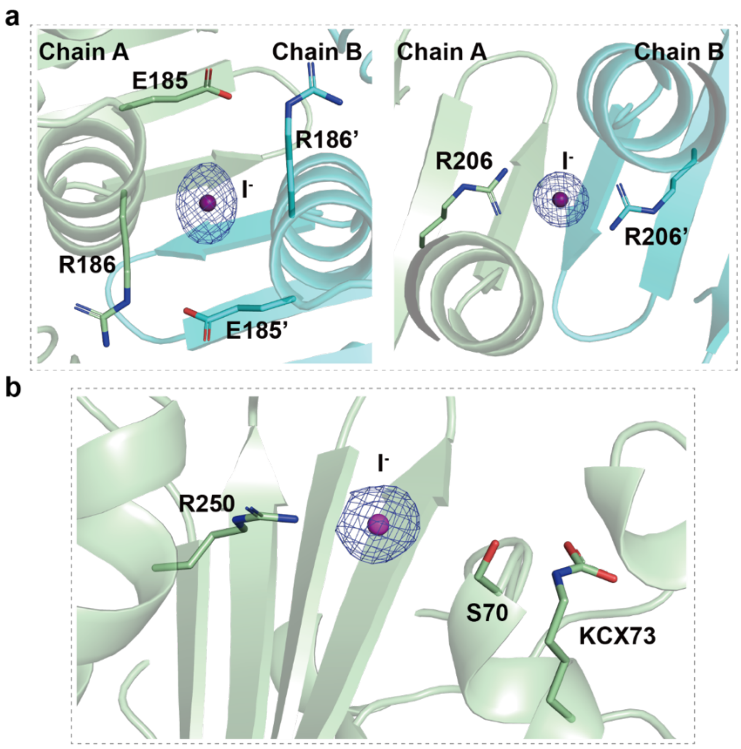

Figure S13 Crystal structure of OXA-48_{WT} with iodide ions.

(**a**) Binding sites for iodide ions at the OXA-48_{WT} dimer interface. (**b**) Binding sites for iodide ions in the OXA-48_{WT} active site. Unbiased $2F_o$−F_c electron density map of iodide is shown as blue mesh, contoured at 1σ (0.3053 e^-/A^3).

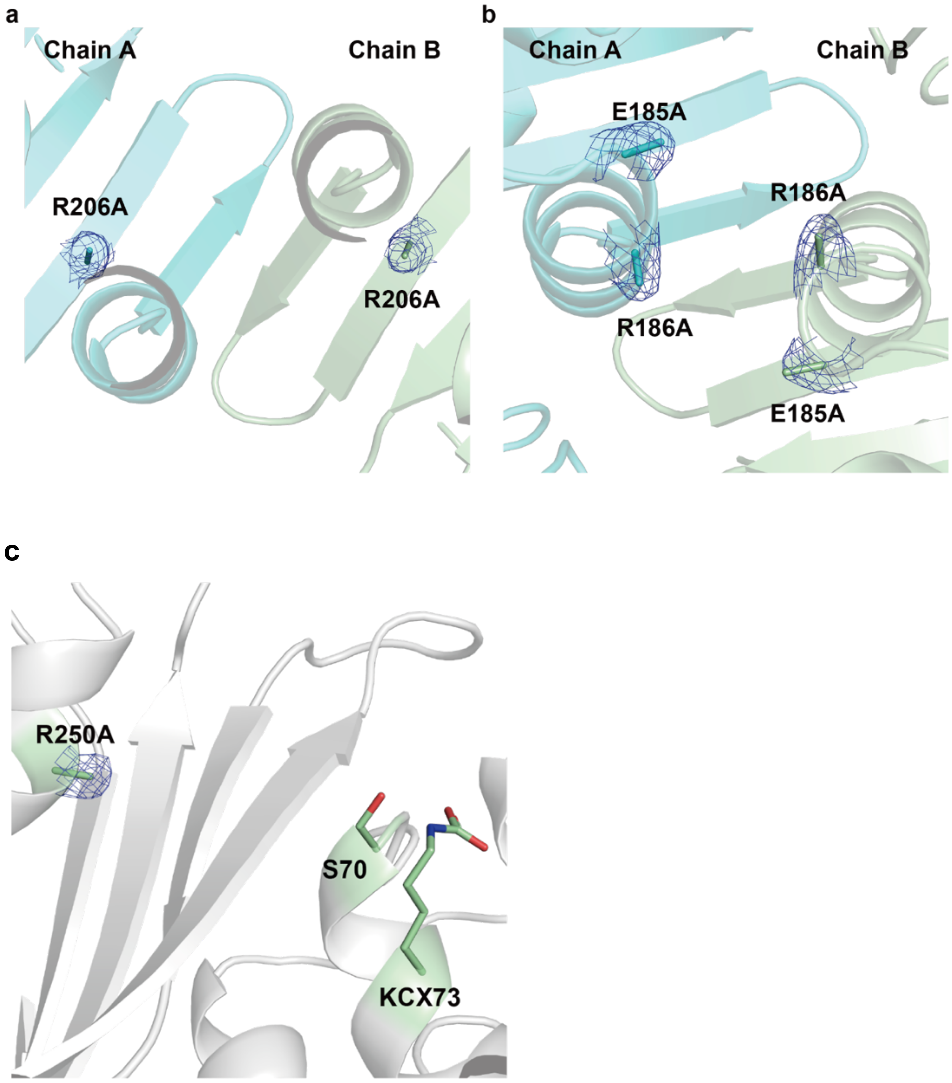

Figure S14 Crystal structures of OXA-48$_{E185A/R186A/R206A}$ (7PEI, **a,b**) and OXA-48$_{R250A}$ (7PGO, **c**) soaked with Br^- to confirm the abolished bromide binding after mutating these residues to alanine. (**a, b**) Unbiased $2F_o{-}F_c$ electron density map of the mutated residues E185A/R186A/R206A is shown as blue mesh, contoured at 1σ (0.2216 e^-/Å^3). (**c**) Unbiased $2F_o{-}F_c$ electron density map of the mutated residue R250A is shown as blue mesh, contoured at 1σ (0.3555 e^-/Å^3).

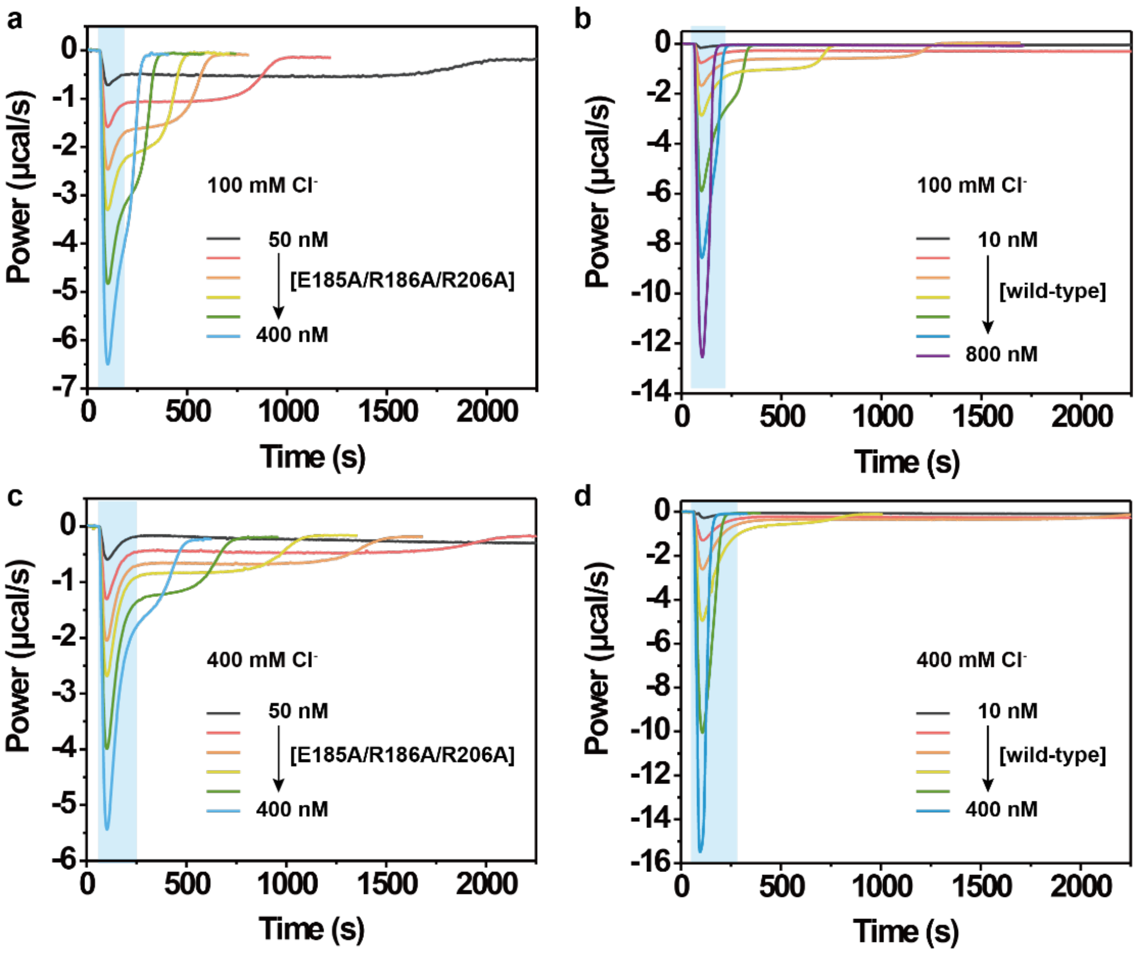

Figure S15 Comparing the OXA-48_{WT} and OXA-$48_{E185A/R186A/R206A}$ for the burst phase for the level of allosteric effect.

Imipenem was hydrolyzed by various concentrations of interface variant OXA-$48_{E185A/R186A/R206A}$ and OXA-48_{WT} in the presence of (**a, b**) 100 mM NaCl and (**c, d**) 400 mM NaCl. Experiments were performed by injecting 10 µL imipenem solution (final concentration 200 µM) into a sample cell filled with 208.7 µL enzyme (final concentration 50-400 nM for OXA-$48_{E185A/R186A/R206A}$, 10-800 nM for OXA-48_{WT}), both in the buffer of 50 mM NaPi, pH 7.5, 1 mM $NaHCO_3$, and 100 mM or 400 mM NaCl.

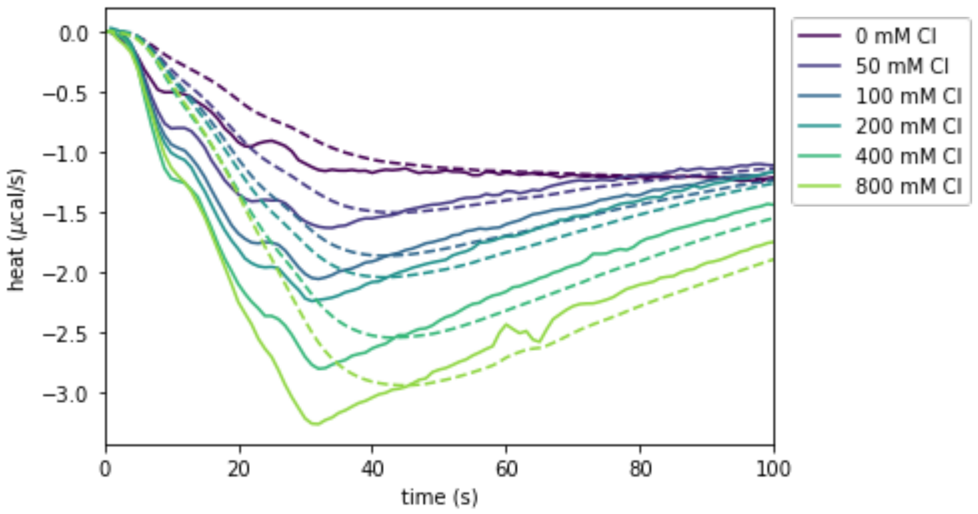

Figure S16 Converting between measured and real heat signal. Solid lines represent experimental data, while dashed lines are simulations of the model for 0 – 800 mM NaCl.

The measured signal in the experiment (dashed lines) and the real signal occurring at a given moment (solid lines) can be interconverted using Eq. 2. The plot shows a close-up view of the first seconds of the experiment, where the burst phase occurs. Note that the measured signal is much smoother than the real signal.

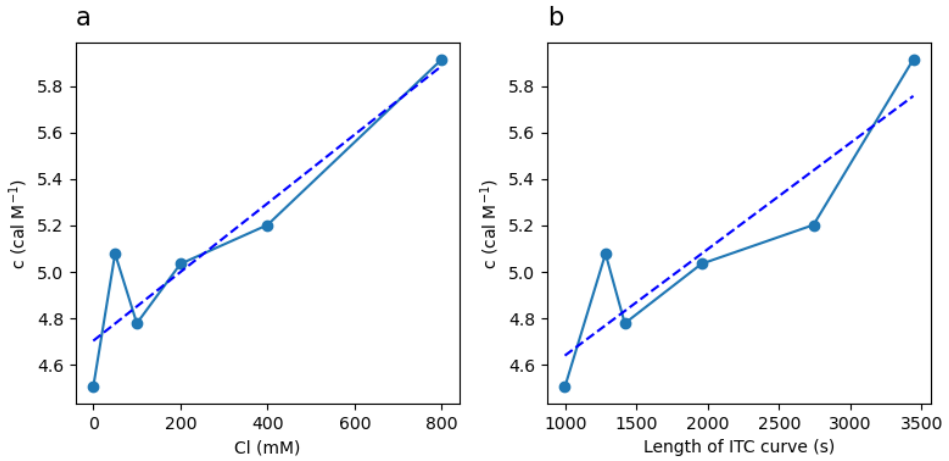

Figure S17 Numerically obtaining *c*, the conversion between moles of substrate and calories of heat generated.

(**a**) This value was obtained for different chloride concentrations, obtaining a linear relationship between chloride concentration and *c*, as follows: c=4.7+0.0015[Cl^-], with an R^2 = 0.871. (**b**) The value of *c* was also linearly related to the length of the ITC curve (L), which increases with chloride: c=4.18+0.0005L, with an R^2 = 0.826.

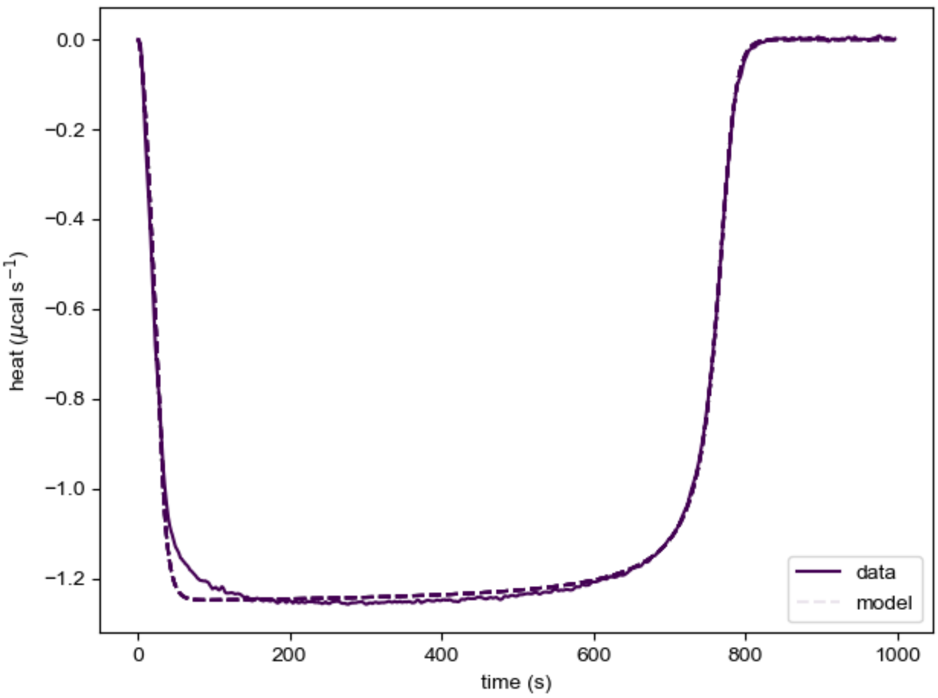

Figure S18 Fifty independent numerical fits to the ITC curve (no chloride) consistently yield k_1'=1.40 μM^{-1} s^{-1} and k_2'=2.81 s^{-1}.

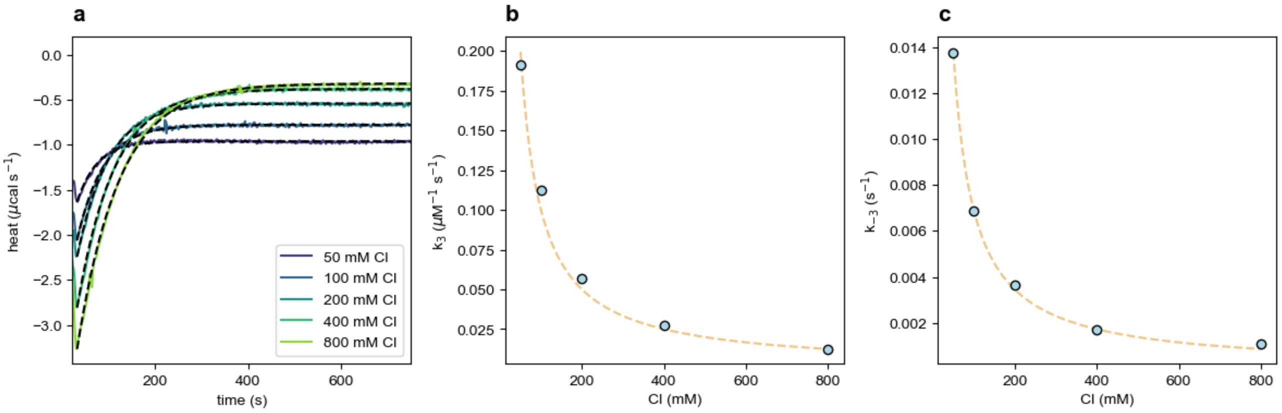

Figure S19 k_3' and k_{-3}' decrease with chloride.

(**a**) The equation

$$C(t) = E_T \frac{k'_{-3}}{k'_3 \mathrm{Cl}^- + k'_{-3}} + E_T \frac{k'_3}{k'_3 \mathrm{Cl}^- + k'_{-3}} e^{-(k'_3 \mathrm{Cl}^- + k'_{-3})t}.$$

is a good fit to our dataset between the end of the initial burst and the stationary phase. (**b**) Fitting these data points to a line, we obtain almost constant values for the slope k_3'[Cl$^-$] (values ranging between 0.0075 and 0.01 s^{-1}), which results in an inverse relationship between k_3' and chloride (solid circles) which is well captured by the inverse curve 9.97/[Cl$^-$] (dashed line), with R^2 = 0.987. (**c**) Using the previous formula to obtain k_{-3}' (solid circles), we observe an inverse relationship with chloride that can be captured with the equation 0.69/[Cl$^-$] (dashed line), with R^2 = 0.999. This relationship implies that k_3'/k_{-3}' ≈ 14.45 μM^{-1}.

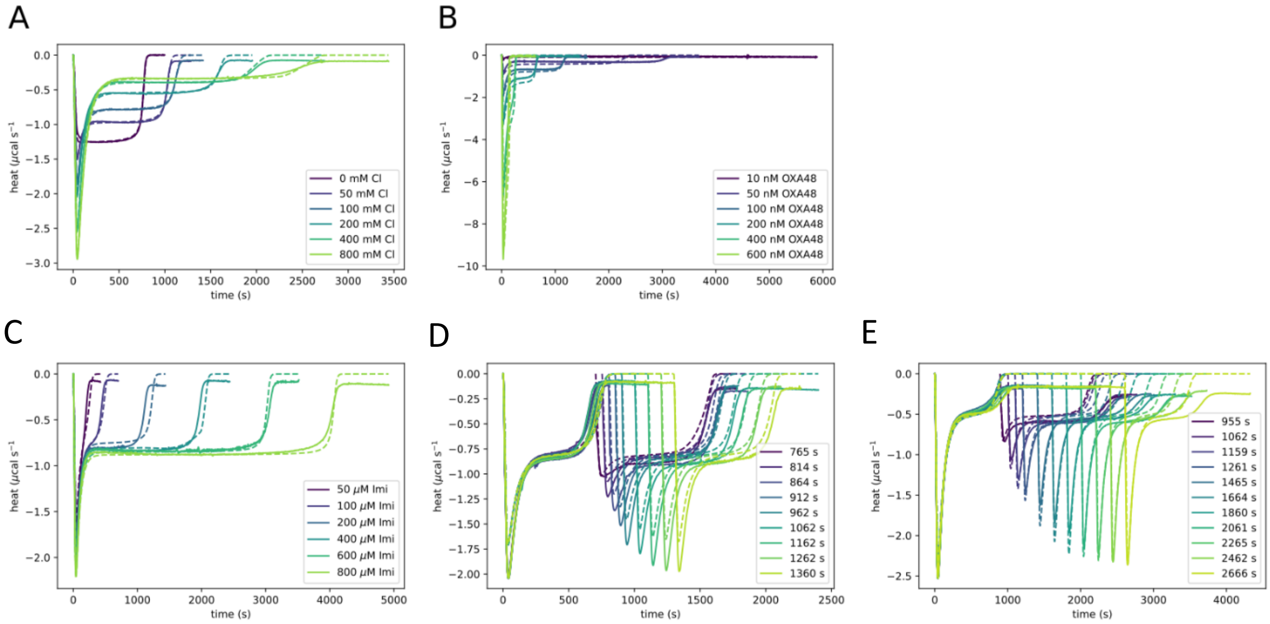

Figure S20 Our mathematical model accurately fits the experimental ITC data including single-injection and double-injection assays. Solid lines represent experimental data, while dashed lines are simulations of the model.

ITC curves were obtained with (**A**) different chloride concentrations, with initial enzyme at 100 nM and initial substrate at 200 μM, (**B**) different enzyme concentrations, with initial enzyme at 100 nM and chloride at 100 mM, (**C**) different substrate concentrations, with initial enzyme at 100 nM and chloride at 100 mM. Finally, the last two panels show the reactivation experiments, where new substrate is injected at different time points. In both cases there is enzyme at 100 nM, with (**D**) chloride at 100 mM and substrate at 180 μM and (**E**) chloride at 400 mM and substrate at 140 μM. In the double-titration experiments, we introduced a progressive delay for the activation of k_3 which helps to capture the peaks. For (D) we used a delay of $37(t_f - 705)/(t_f - 600)$, where t_f is the reinjection time, while for (E) we used $37(t_f - 900)/(t_f - 700)$.

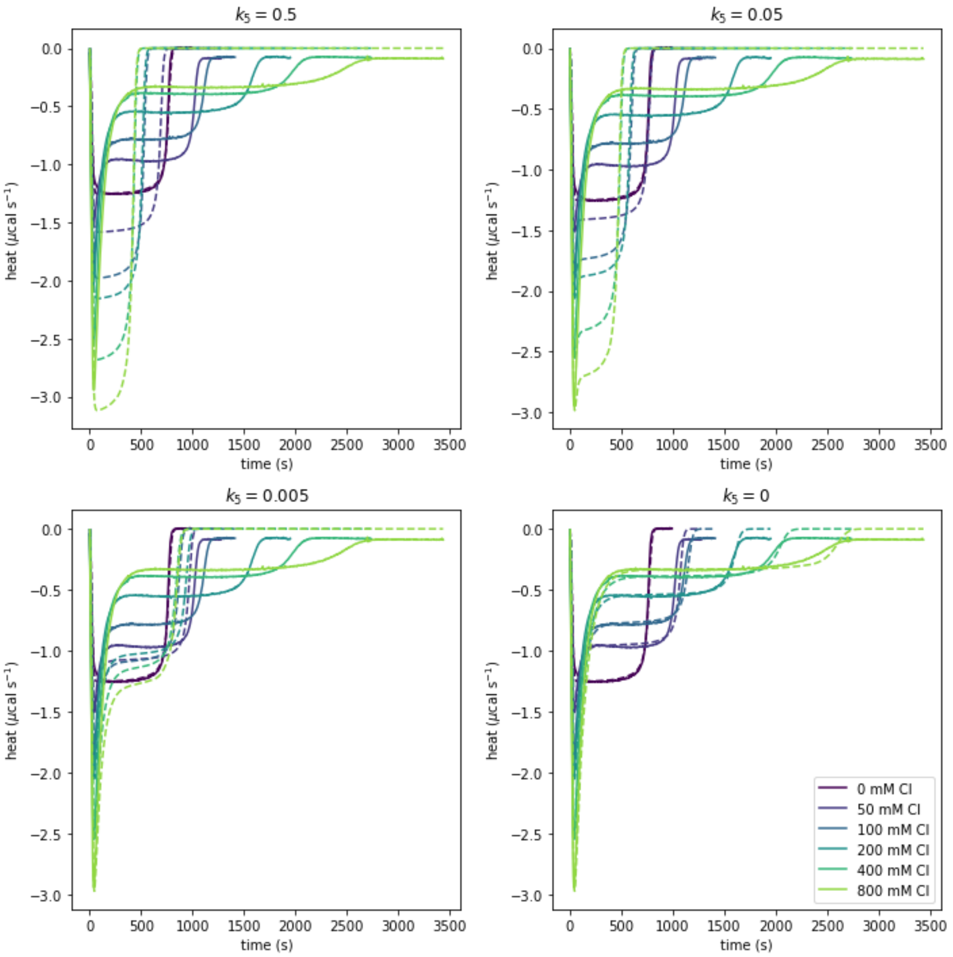

Figure S21 Introducing k_5 fails to capture the ITC data even for very low values from 0 to 0.5 s^{-1}, suggesting that there is no hydrolysis from the inactive intermediate leading to product.

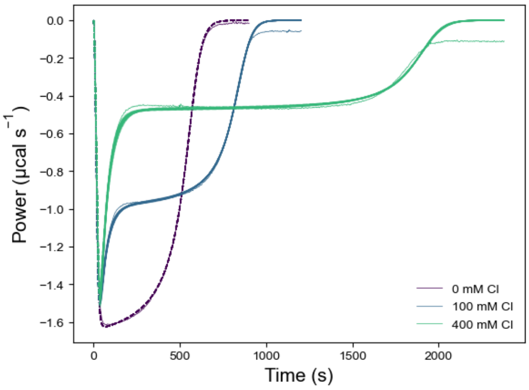

Figure S22 Simulated and experimental apparent reaction rates for the interface variant OXA-$48_{E185A/R186A/R206A}$.

Fifty different parameter sets obtained from numerical optimization are plotted per chloride treatment, all with good fits to the data.

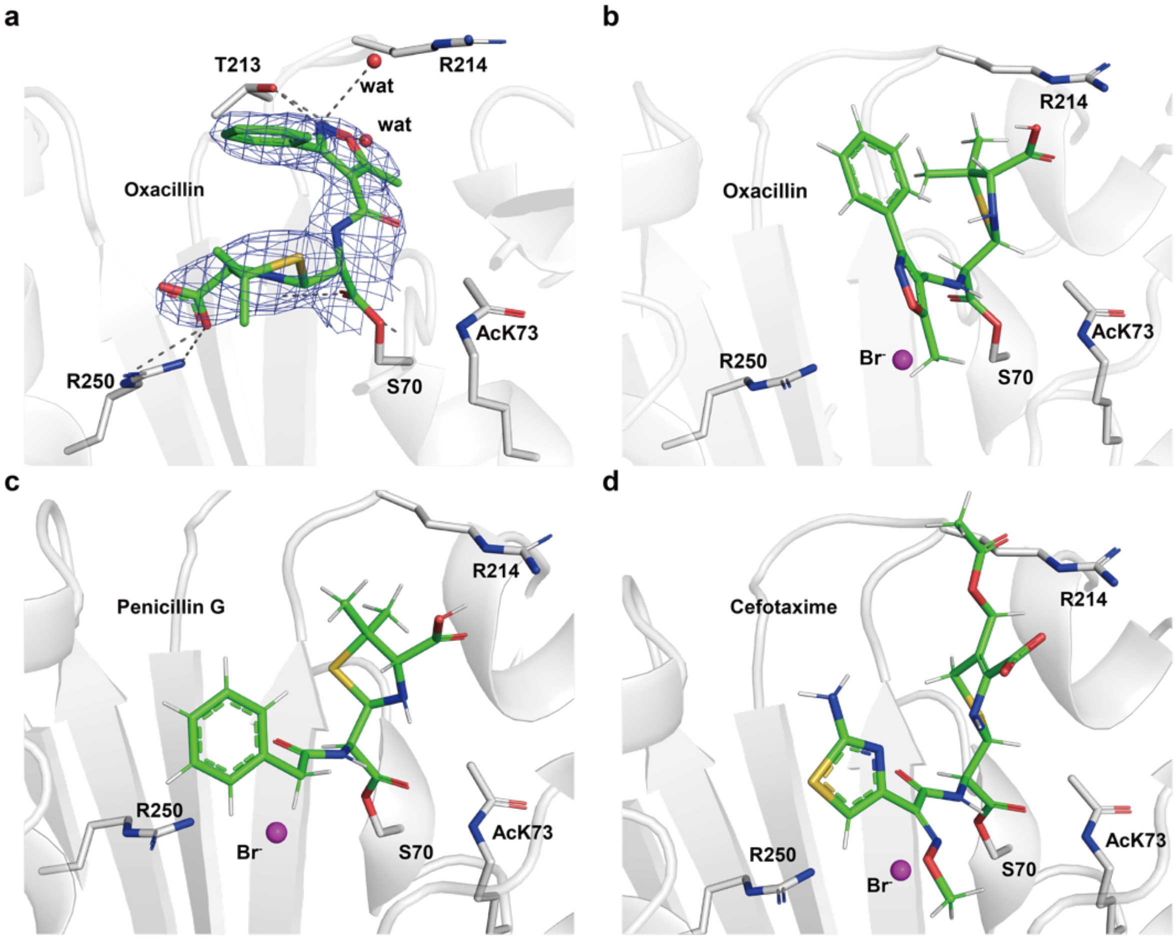

Figure S23 Crystal structure of oxacillin bound in an active conformation, compared to docked structures of large substrates with bromide in inactive conformations in OXA-48$_{AcK73}$.

(**a**) The crystal structures of OXA-48$_{AcK73}$-oxacillin acyl-intermediate complex captured in a crystallization condition free of chloride, (**b**) the docked OXA-48$_{AcK73}$-oxacillin acyl-intermediate with bromide in an inactive conformation, (**c**) the docked OXA-48$_{AcK73}$-penicillin G acyl-intermediate with bromide, and (**d**) the docked OXA-48$_{AcK73}$-cefotaxime acyl-intermediate with bromide.

The initial unbiased $2F_o-F_c$ electron density map of oxacillin in (**a**) is shown as blue mesh, contoured at 1σ (0.2087 $e^-/Å^3$). The structure of (**b**) oxacillin, (**c**) penicillin G, and (**d**) cefotaxime acyl-intermediate (in green) with bromide (in purple) binding at OXA-48$_{AcK}$ active site (in grey) was generated by molecular docking using MOE[1]. The docking was generated from the structure with OXA-48$_{AcK}$-imipenem-Br^- complex H chain. Protein hydrogens are hidden for clarity.

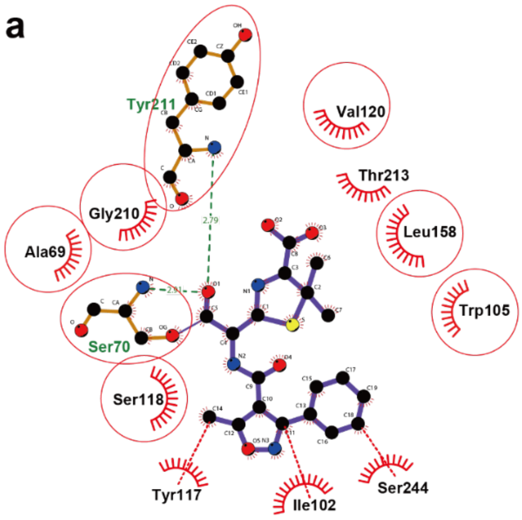

OXA-48$_{AcK73}$/Oxacillin OXA-48$_{AcK73}$/Imipenem

Figure S24 Ligplot diagrams show the hydrogen bonding and hydrophobic interactions between OXA-48$_{AcK73}$ with the inactive acyl-intermediate conformations of (**a**) Oxacillin, (**b**) Imipenem.

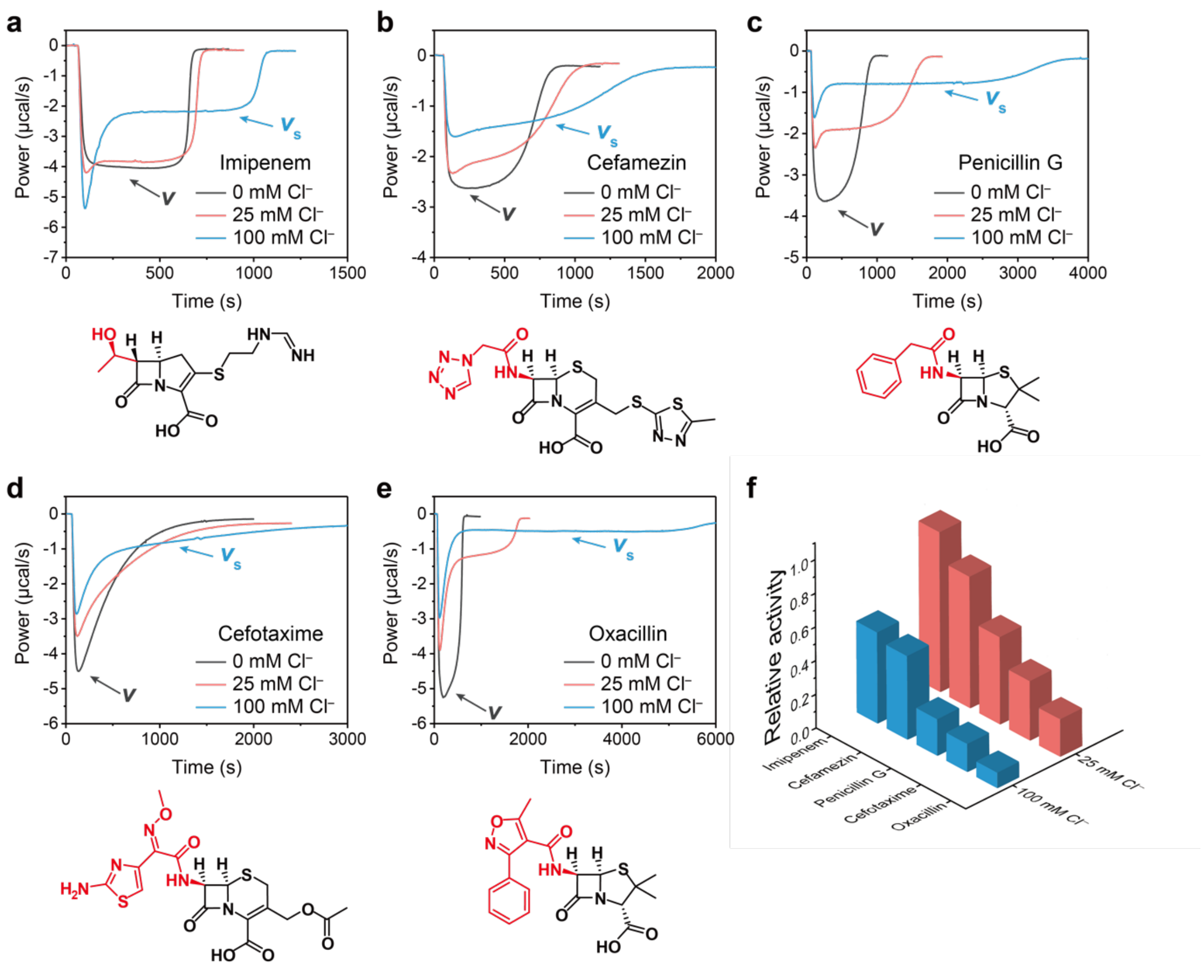

Figure S25 The generality of halide-induced biphasic kinetics.
(**a-e**) Kinetic curves for a series of substrates hydrolysis by OXA-48_{WT} with 0, 25 mM or 100 mM Cl^-. The side chain groups of different antibiotic substrates are shown in red. (**f**) The relative steady-state activities v_s/v of OXA-48_{WT} hydrolyzing different antibiotics in the presence of 25 mM or 100 mM Cl^-.

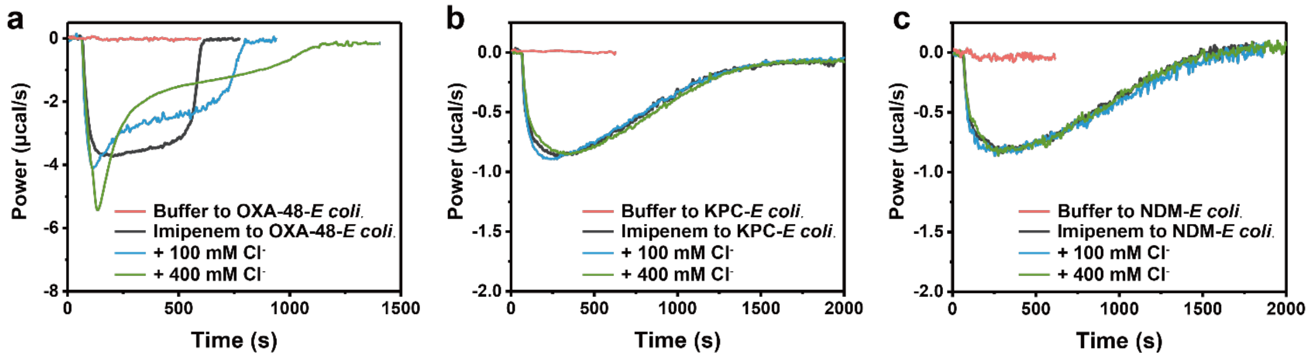

Figure S26 Thermogram curves of imipenem hydrolysis in the absence and presence of chloride by cell suspensions.
(**a**) OXA-48-BL21 star (DE3) *E. coli*, (**b**) KPC-BL21 star (DE3) *E. coli* and (**c**) NDM-BL21 star (DE3) *E. coli*, in 50 mM NaP_i buffer, pH 7.5, and 0.1 mM $ZnSO_4$.

CLUSTAL O(1.2.4) multiple sequence alignment

```
OXA-48       ----MRVLALSAV-FLVASIIGM---------PAVAKEWQENKSWNAHFTEHKSQGVVVL      46
OXA-2        -----MAIRIFAILFSIFSLATF---------AHAQEGTLERSDWRKFFSEFQAKGTIVV      46
OXA-10       -------MKTFAA-YVIIACLSS---------TALAGSITENTSWNKEFSAEAVNGVFVL      43
OXA-14       -------MKTFAA-YVIIACLSS---------TALAGSITENTSWNKEFSAEAVNGVFVL      43
OXA-16       -----------------IACLSS---------TALAGSITENTSWNKEFSAEAVNGVFVL      34
OXA-27       MNKYFTCYVVA--SLFLSGCTVQHNL---INETPSQIVQGHNQVIHQYFDEKNTSGVLVI      55
OXA-50       ----MRPLLFSA-LLLLSGH-------------TQASEWNDSQAVDKLFGAAGVKGTFVL      42
OXA-24       MKKFILPIFSISILVSLSACSSIKTK---SEDNFHISSQQHEKAIKSYFDEAQTQGVIII      57
OXA-25       MKKFILPIFSISILVSLSACSSIKTK---SEDNFHISSQQHEKAIKSYFDEAQTQGVIII      57
OXA-26       MKKFILPIFSISILVSLSACSSIKTK---SEDNFHISSQQHEKAIKSYFDEAQTQGVIII      57
OXA-40       MKKFILPIFSISILVSLSACSSIKTK---SEDNFHISSQQHEKAIKSYFDEAQTQGVIII      57
OXA-58       -MKLLKILSLVCLSISIGACAEHSMSRAKTSTIPQVNNSIIDQNVQALFNEISADAVFVT      59
OXA-163      ----MRVLALSAV-FLVASIIGM---------PAVAKEWQENKSWNAHFTEHKSQGVVVL      46
                               .                             *     ....:

OXA-48       WNENKQQ--GFTNNLKRANQAFLPASTFKIPNSLIALDLGVVKDEHQVFKWDGQTRDIAT      104
OXA-2        ADERQADRAMLVFDPVRSKKRYSPASTFKIPHTLFALDAGAVRDEFQIFRWDGVNRGFAG      106
OXA-10       CKSSSKS--CATNDLARASKEYLPASTFKIPNAIIGLETGVIKNEHQVFKWDGKPRAMKQ      101
OXA-14       CKSSSKS--CATNDLARASKEYLPASTFKIPNAIIGLETGVIKNEHQVFKWDGKPRAMKQ      101
OXA-16       CKSSSKS--CATNDLARASKEYLPASTFKIPNAIIGLETGVIKNEHQVFKWDGKPRAMKQ      92
OXA-27       QTDKKIN--LYGNALSRANTEYVPASTFKMLNALIGLENQKA-DINEIFKWKGEKRSFTA      112
OXA-50       YDVQRQR--YVGHDRERAETRFVPASTYKVANSLIGLSTGAVRSADEVLSYGGKPQRFKA      100
OXA-24       KEGKNLS--TYGNALARANKEYVPASTFKMLNALIGLENHKA-TTNEIFKWDGKKRTYPM      114
OXA-25       KEGKNLS--TYGNALARANKEYVPASTFKMLNALIGLENHKA-TTNEIFKWDGKKRTYPM      114
OXA-26       KEGKNLS--TYGNALARANKEYVPASTFKMLNALIGLENHKA-TTNEIFKWDGKKRTYPM      114
OXA-40       KEGKNLS--TYGNALARANKEYVPASTFKMLNALIGLENHKA-TTNEIFKWDGKKRTYPM      114
OXA-58       YDGQNIK--KYGTHLDRAKTAYIPASTFKIANALIGLENHKA-TSTEIFKWDGKPRFFKA      116
OXA-163      WNENKQQ--GFTNNLKRANQAFLPASTFKIPNSLIALDLGVVKDEHQVFKWDGQTRDIAT      104
                             *:.  : ****:*: ::::.*.        ::: : *  :

OXA-48       WNRDHNLITAMKYSVVPVYQEFARQIGEARMSKMLHAFDYGNEDISGNVDSFWLDGGIRI      164
OXA-2        HNQDQDLRSAMRNSTVWVYELFAKEIGDDKARRYLKKIDYGNADPSTSNGDYWIEGSLAI      166
OXA-10       WERDLTLRGAIQVSAVPVFQQIAREVGEVRMQKYLKKFSYGNQNISGGIDKFWLEGQLRI      161
OXA-14       WERDLTLRGAIQVSAVPVFQQIAREVGEVRMQKYLKKFSYGNQNISGGIDKFWLEDQLRI      161
OXA-16       WERDLTLRGAIQVSAVPVFQQITREVGEVRMQKYLKKFSYGNQNISGGIDKFWLEDQLRI      152
OXA-27       WEKDMTLGEAMKLSAVPVYQELARRIGLDLMQKEVKRIGFGNAEIGQQVDNFWLVGPLKV      172
OXA-50       WEHDMSLRDAIKASNVPVYQELARRIGLERMRANVSRLGYGNAEIGQVVDNFWLVGPLKI      160
OXA-24       WEKDMTLGEAMALSAVPVYQELARRTGLELMQKEVKRVNFGNTNIGTQVDNFWLVGPLKI      174
OXA-25       WEKDMTLGEAMALSAVPVYQELARRTGLELMQKEVKRVNFGNTNIGTQVDNFWLVGPLKI      174
OXA-26       WEKDMTLGEAMALSAVPVYQELARRTGLELMQKEVKRVNFGNTNIGTQVDNFWLVGPLKI      174
OXA-40       WEKDMTLGEAMALSAVPVYQELARRTGLELMQKEVKRVNFGNTNIGTQVDNFWLVGPLKI      174
OXA-58       WDKDFTLGEAMQASTVPVYQELARRIGPSLMQSELQRIGYGNMQIGTEVDQFWLKGPLTI      176
OXA-163      WNRDHNLITAMKYSVVPVYQEFARQIGEARMSKMLHAFDYGNEDISGNVDSFWLDGGIRI      164
              ::*  *  *:  * * *:: :::. *       :  ..:** : .   ..:*: . : :
```

```
OXA-48        SATEQISFLRKLYHNKLHVSERSQRIVKQAMLTEANGDYIIRAKTGYS---TRIEPKIGW      221
OXA-2         SAQEQIAFLRKLYRNELPFRVEHQRLVKDLMIVEAGRNWILRAKTGWEG-------RMGW      219
OXA-10        SAVNQVEFLESLYLNKLSASKENQLIVKEALVTEAAPEYLVHSKTGFSGVGTESNPGVAW      221
OXA-14        SAVNQVEFLESLYLNKLSASKENQLIVKEALVTEAAPEYLVHSKTGFSGVGTESNPGVAW      221
OXA-16        SAVNQVEFLESLYLNKLSASKENQLIVKEALVTEAAPEYLVHSKTGFSGVGTESNPGVAW      212
OXA-27        TPIQEVEFVSQLAHTQLPFSEKVQANVKNMLLLEESNGYKIFGKTGWA---MDIKPQVGW      229
OXA-50        SAMEQTRFLLRLAQGELPFPAPVQSTVRAMTLLESSPGWELHGKTGWC---FDCTPELGW      217
OXA-24        TPVQEVNFADDLAHNRLPFKLETQEEVKKMLLIKEVNGSKIYAKSGWG---MGVTPQVGW      231
OXA-25        TPVQEVNFADDLAHNRLPFKLETQEEVEKMLLIKEVNGSKIYAKSGWG---MGVTPQVGW      231
OXA-26        TPVQEVNFADDLAHNRLPFKLETQEEVKKMLLIKEVNGSKIYAKSGWG---MGVTPQVGW      231
OXA-40        TPVQEVNFADDLAHNRLPFKLETQEEVKKMLLIKEVNGSKIYAKSGWG---MGVTPQVGW      231
OXA-58        TPIQEVKFVYDLAQGQLPFKPEVQQQVKEMLYVERRGENRLYAKSGWG---MAVDPQVGW      233
OXA-163       SATEQISFLRKLYHNKLHVSERSQRIVKQAMLTEANGDYIIRAKTGYD---T----KIGW      217
              :  ::  *   *   .*      *  *.     :      : .*:*:          :.*

OXA-48        WVGWVELDD-NVWFFAMNMDMPTSDG-LGLRQAITKEVLKQEKIIP-----------  265
OXA-2         WVGWVEWPT-GSVFFALNIDTPNRMDDLFKREAIVRAILRSIEALPPNPAVNSDAAR  275
OXA-10        WVGWVEKET-EVYFFAFNMDIDNESK-LPLRKSIPTKIMESEGIIGG----------  266
OXA-14        WVGWVEKET-EVYFFAFNMDIDNESK-LPLRKSIPTKIMESEGIIGG----------  266
OXA-16        WVGWVEKET-EVYFFAFNMDIDNESK-LPLRKSIPTKIMESEGIIGG----------  257
OXA-27        LTGWVEQPDGKIVAFALKMEMRSEMP-ASIRNELLMKSLKQLNII------------  273
OXA-50        WVGWVKRN-ERLYGFALNIDMPGGEADIGKRVELGKASLKALGILP-----------  262
OXA-24        LTGWVEQANGKKIPFSLNLEMKEGMS-GSIRNEITYKSLENLGII------------  275
OXA-25        LTGWVEQANGKKIPFSLNLEMKEGMS-GSIRNEITYKLLENLGII------------  275
OXA-26        LTGWVEQANGKKIPFSLNLEMKEGMT-GSIRNEITYKSLENLGII------------  275
OXA-40        LTGWVEQANGKKIPFSLNLEMKEGMS-GSIRNEITYKSLENLGII------------  275
OXA-58        YVGFVEKADGQVVAFALNMQMKAGDD-IALRKQLSLDVLDKLGVFHYL---------  280
OXA-163       WVGWVELDD-NVWFFAMNMDMPTSDG-LGLRQAITKEVLKQEKIIP-----------  261
               .*:*:        *:::::          *  :    :     :
```

Figure S27 Sequence alignment of OXA-48 like carbapenemases to show R250 is strictly conserved. It predicts that the biphasic kinetics involving R250 and chloride ion are probably highly common. By comparison, R214 is not conserved.

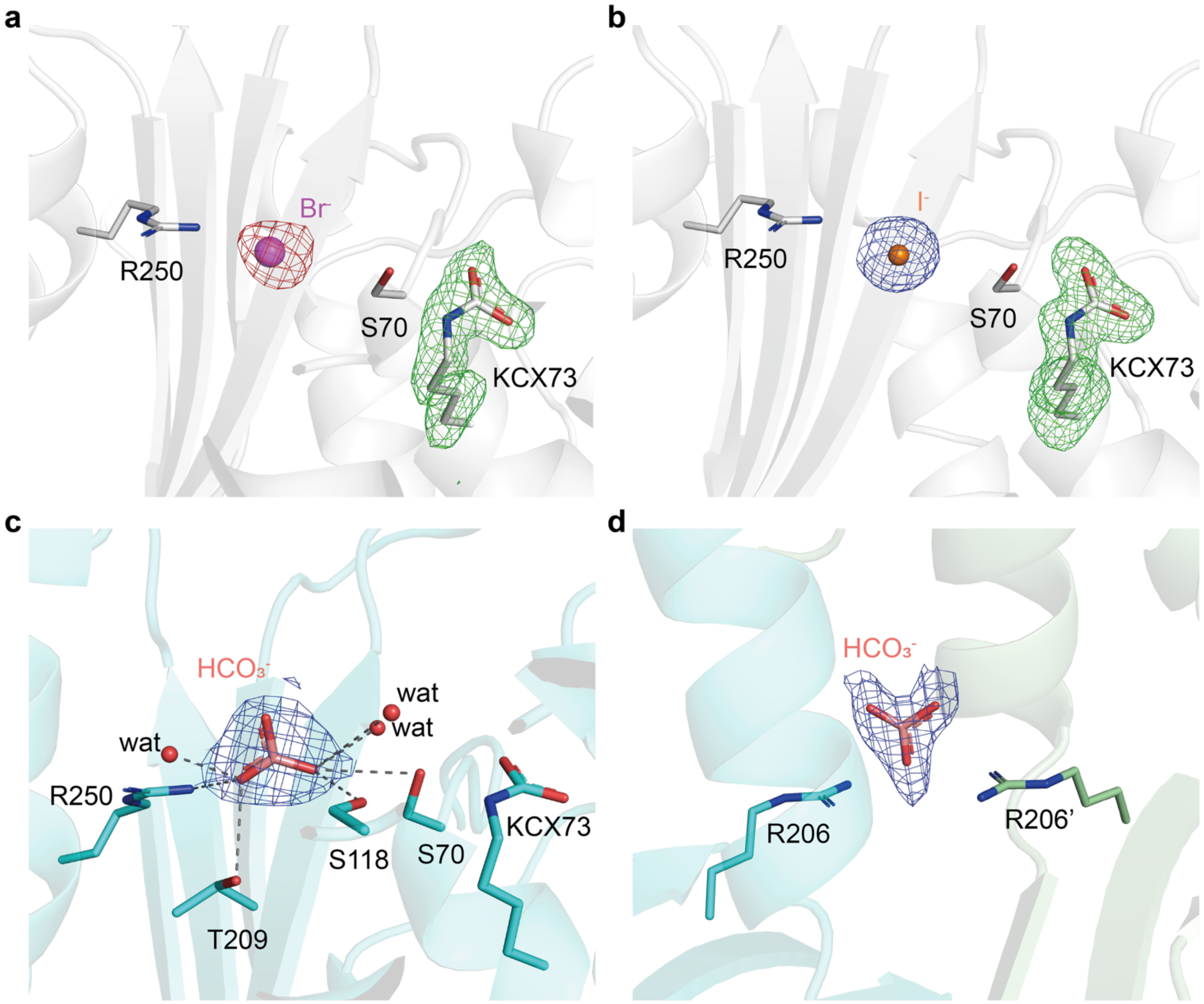

Figure S28 Crystal structures of OXA-48_{WT} soaked with different anions.
The intact electron density of KCX in OXA-48_{WT}-Br^- complex structure (**a**) and OXA-48_{WT}-I^- complex structure (**b**) both show halide itself does not cause decarbamylation. The initial F_o−F_c electron density map of KCX (green mesh) are contoured at 3σ (0.3648 and 0.2797 $e^-/Å^3$, respectively). The OXA-48_{WT}-HCO_3^- complex structure shows HCO_3^- ion bound to R250 (**c**) and at the dimer interface R206-R206' (**d**). The initial unbiased 2F_o−F_c electron density map of HCO_3^- is shown as blue mesh, contoured at 1σ (0.3625 $e^-/Å^3$).

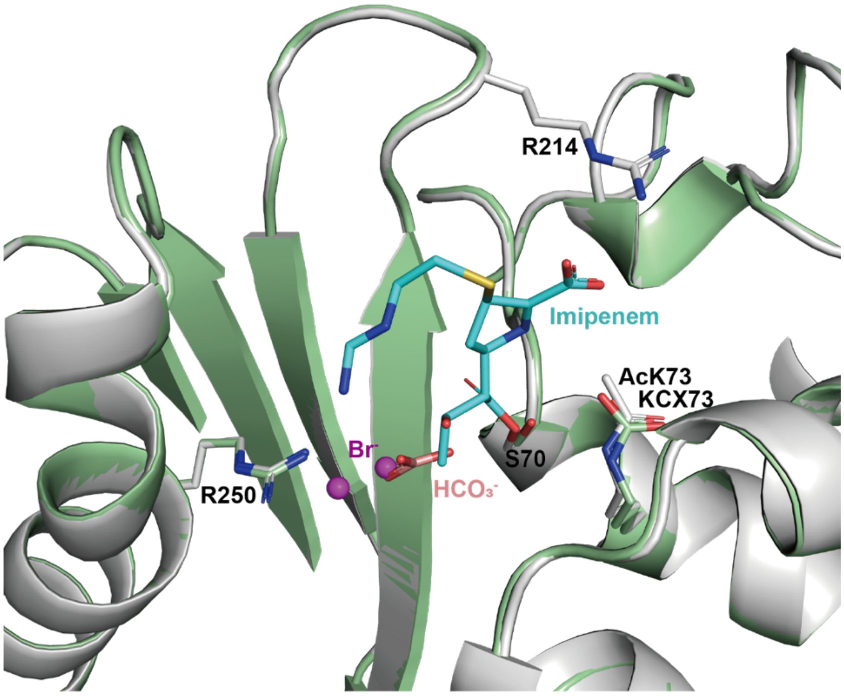

Figure S29 Superimposition of the OXA-48$_{AcK73}$-imipenem-Br^- acyl-intermediate complex in the inactive conformation with the OXA-48$_{WT}$-HCO_3^- complex.

OXA-48$_{AcK73}$-imipenem-Br^- acyl-intermediate: protein in grey, imipenem in cyan, and bromide in purple. OXA-48$_{WT}$-HCO_3^- complex: protein in green and HCO_3^- in salmon.

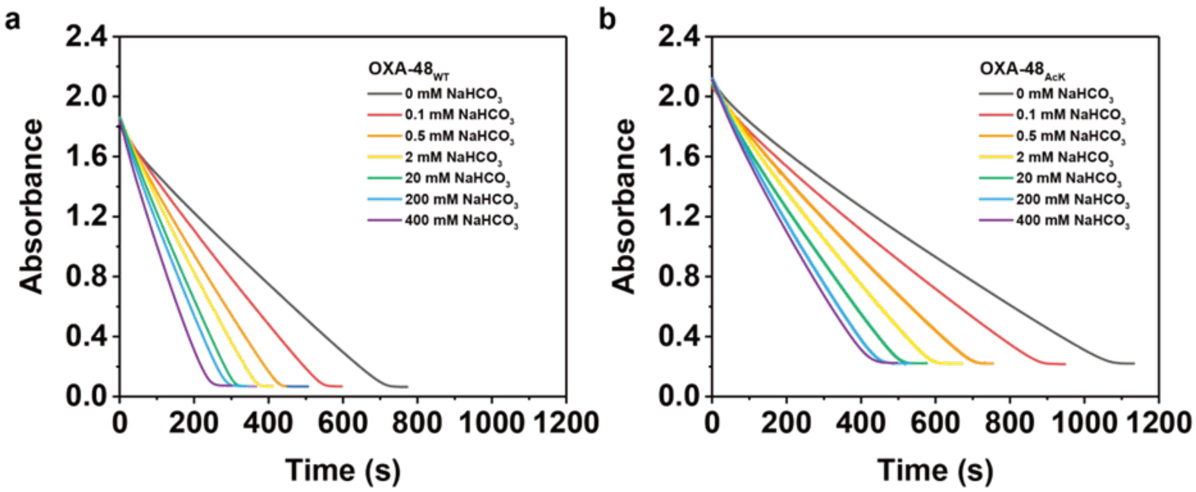

Figure S30 HCO_3^- is a weak allosteric effector of OXA-48.

UV-vis spectra of 200 μM imipenem hydrolysis by (**a**) 100 nM OXA-48$_{WT}$ and (**b**) 8 μM OXA-48$_{AcK73}$ in 50 mM NaPi buffer, pH 7.5 supplemented with 0-400 mM HCO_3^-.

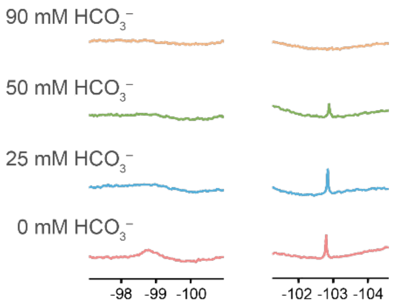

Figure S31 ^{19}F NMR spectra of a sample of 500 μM OXA-48$_{WT}$ in the buffer of 500 mM NH_4F, 100 mM NaPi, pH 7.5, 10% D_2O titrated with 0-90 mM $NaHCO_3$ to show HCO_3^- ion outcompetes fluoride at lower concentrations for at least two binding sites.

General information.

Imipenem was purchased from Chengdu Ai Keda Chemical Technology Co. Ltd. (China) and Merck (UK). All the other antibiotics were purchased from TCI (TCI Shanghai, China) and Merck (UK). All the non-antibiotic chemicals used in this study were analytical grade. The clinical isolates of carbapenemase-producing *Klebsiella pneumoniae* were kindly supplied by Dr Jin-E Lei from the First Affiliated Hospital of Xi'an Jiaotong University (Xi'an, China) and had been previously confirmed by DNA sequencing[2]. No unexpected or unusually high safety hazards were encountered.

Bacterial growth assay.

Bacterial growth assays were performed using clinical *Klebsiella pneumoniae* strains expressing carbapenemases OXA-48, KPC or NDM gene, respectively. The spectrophotometric method was used to detect bacterial growth through the measurement of the opacity of the cultures (at λ = 600 nm). The bacteria cultured overnight (same opacity in TB medium) were diluted 1000 times into TB medium containing antibiotics (20 μg/mL imipenem), chloride (400 mM) or both, respectively. These bacteria cultures were incubated at 37 °C, 180 rpm for 12 h. The optical density (λ = 600 nm) of the bacteria was monitored using a spectrophotometer (Varioskan Flash, Thermo Fisher Scientific) every 1 h during the incubation.

Calorimetric assays for living bacteria.

Cell-based calorimetric assays were performed using clinical *Klebsiella pneumoniae* strains expressing OXA-48, KPC or NDM gene. Bacterial suspensions were prepared as described previously[2]. Experiments were performed by injecting 10 μL imipenem stock solution (final concentration 400 μM), preloaded in the syringe, into a sample cell filled with 210 μL of bacterial suspensions (OD_{600} = 4.0 for OXA48-*Kp*, OD_{600} = 1.5 for KPC-*Kp*, OD_{600} = 0.1 for NDM-*Kp*), both prepared in the buffer (50 mM NaPi, pH = 7.5, 1 mM $NaHCO_3$, with 0-400 mM sodium chloride, 1 mM $ZnSO_4$ added for NDM-*Kp*). The control experiment was performed by injecting buffer into bacterial suspensions only. Real-time changes in heat-flow were recorded continuously until returning to baseline.

Molecular cloning.

The *bla*OXA-48 gene (GenBank: AAP70012.1) from residue K23 was codon-optimized for *E. coli*, synthesized (Sangon Biotech) and cloned into the pET9a expression vector using the *NdeI*/*BamHI* restriction sites. The resulting plasmid encodes an N-terminally His_6-tagged protein.

Site-directed mutagenesis.

The mutations in this work were introduced into OXA-48_{WT} by site-directed mutagenesis. 20 ng of pET9a-*bla*OXA-48 template DNA was mixed with 10 μL 5x Phusion^{TM} HF buffer, 0.2 μM forward and

0.2 μM reverse primer pairs (**Table S6**), 0.2 mM dNTP-mix, 1.5 μL DMSO, and 1 U Phusion™ High–Fidelity DNA Polymerase (Thermo Fisher Scientific; Waltham, USA) in a 50 μL reaction. The PCR reaction was performed with initial 3 min denaturation at 98 °C, followed by 30 cycles of 40 s denaturation at 98 °C, 15 s annealing at 63 °C, and 2.5 min extension at 72 °C. And a final extension was performed at 72 °C for 10 min before cooled to 4 °C. PCR products were immediately digested by *DpnI* (Thermo Fisher Scientific; Waltham, USA) for 2 h at 37 °C and transformed into *E. coli* TOP10 competent cells. The plasmids were extracted and purified, and subsequently verified by sequencing.

Table S6 Oligonucleotides used for sequencing and mutagenesis.

Primer	Sequence
T7	TAATACGACTCACTATAGGG
T7-term	GCTAGTTATTGCTCAGCGG
OXA48-R214A-f	GAAAACCGGTTACAGCACC**GCT**ATCGAACCGAAAATCGG
OXA48-R214A-r	CCGATTTTCGGTTCGAT**AGC**GGTGCTGTAACCGGTTTTC
OXA48-R250A-f	GATGGTCTGGGACTG**GCT**CAAGCGATCACCAAAGAAG
OXA48-R250A-r	CTTCTTTGGTGATCGCTTG**AGC**CAGTCCCAGACCATC
OXA48-E185A/R186A -f	CTGCACGTTAGC**GCGGCC**TCTCAG
OXA48-E185A/R186A-r	CTGAGA**GGCCGC**GCTAACGTGCAG
OXA48-R206A-f	GAACGGCGATTACATCATT**GCT**GCGAAAACC
OXA48-R206A-r	GGTTTTCGC**AGC**AATGATGTAATCGCCGTTC
OXA48-AcK73-f	GGCGTCTACTTTC**TAG**ATCCCGAACAGCCTG
OXA48-AcK73-r	CAGGCTGTTCGGGAT**CTA**GAAAGTAGACGCC

Gene expression and protein purification of OXA-48$_{WT}$ and its variants OXA-48$_{R250A}$, OXA-48$_{R214A}$, and OXA-48$_{E185A/R186A/R206A}$.

All OXA-48$_{WT}$ and its variants OXA-48$_{R250A}$, OXA-48$_{R214A}$, and OXA-48$_{E185A/R186A/R206A}$ were expressed in *E. coli* BL21 Star (DE3) strain (New England Biolabs; Ipswich, USA). The transformed cells were selected with 100 μg/mL Kanamycin (TCI) on Terrific Broth (TB) agar medium (11.8 g/L tryptone, 23.6 g/L yeast extract, 9.4 g/L K_2HPO_4, 2.2 g/L KH_2PO_4 and 4 mL/L glycerin) by overnight incubation at 37 °C. Transformed cells were grown in TB media containing 100 μg/mL Kanamycin at 37 °C until the OD_{600} reached 0.6. The culture was cooled to 18 °C before a final concentration of 0.1 mM isopropyl-β-D-thiogalactopyranoside (IPTG, Sigma-Aldrich) was added, and then further incubated for 24 h at 160 rpm. Cells were subsequently harvested by centrifugation at 8000 rpm for 10 min at 4 °C, resuspended in 20 mL buffer A (150 mM NaPi, 25 mM imidazole, pH 7.5) with lysozyme (Sangon Biotech, Shanghai, 0.5 mg/mL), and incubated at 4 °C for 30 min. The cells were lysed by sonication on ice (pulse interval, 2s/2s (on/off); duration, 30 min) and the lysate was centrifuged at 18000 rpm, 4 °C for 60 min. The supernatant was filtered through a 0.45 μm syringe filter before being loaded onto a 5 mL HisTrap™ column (GE Healthcare) which had been pre-equilibrated with 50 mL buffer A. Subsequently, the column was washed with 50 mL buffer A and the protein was eluted with 20 mL

buffer B (150 mM NaPi, 250 mM imidazole, pH 7.5). The fractions containing the eluted protein, confirmed by sodium dodecyl sulfate-polyacrylamide gel electrophoresis (SDS-PAGE), was concentrated and further purified by size exclusion chromatography (SEC) on a HiLoad 16/600 Superdex 200pg column (GE Healthcare) with 100 mM HEPES buffer, pH 7.5. Purity of the collected sample was checked by SDS-PAGE before buffer exchange into required buffers for further experiments.

Gene expression and protein purification of OXA-48$_{AcK73}$.

BL21(DE3) was co-transformed with pET9a-OXA48-K73TAG plasmid and the plasmid of the tRNA synthetase by heat shock at 42 °C for 30 seconds. The cells were recovered in 950 µL SOC for 2 h at 37 °C before 100 µL was spread on an agar plate containing kanamycin (50 µg/mL) and spectinomycin (100 µg/mL). LB containing kanamycin (50 µg/mL) and spectinomycin (100 µg/mL) was inoculated with a single colony of transformed cells and incubated at 37 °C for 16 h at 180 rpm. Although AcK73 in the active site is virtually inaccessible for the deacetylases from the *E. coli* strain used, an extra step of caution was taken by adding nicotinamide to fully inhibit potential deacetylase activity in the culture. Thus, the inoculated LB media contained 50 µg/mL kanamycin and 100 µg/mL spectinomycin and were incubated at 37 °C. At OD_{600} = 0.4, nicotinamide (20 mM) and L-acetyllysine (5 mM) were added to the media. After 30 mins, protein expression was induced with 0.5 mM IPTG and the cultures were incubated at 16 °C for 18 hours. The media was centrifuged at 6000 rpm for 20 mins at 4 °C. The cell paste was re-suspended in 30 mL of buffer (100 mM NaPi, 20 mM imidazole, pH 8.0) containing PMSF (0.5 mM). Cells were lysed using a sonication cycle of 10 s/50 s (on/off) for 6 minutes at 4 °C. Soluble material was separated from cell debris by centrifugation at 18000 rpm for 50 mins at 4 °C. The supernatant was applied to a pre-equilibrated Ni-NTA column and washed with five column volumes of the same buffer, and step gradients of increasing imidazole were used for washing and elution steps. Based on SDS-PAGE, elution fractions collected using 200 mM imidazole were concentrated with a centrifugal concentrator (10,000 MWCO). The mixture was diluted with buffer (100 mM NaPi, pH 7.5) to remove residual imidazole.

General procedure for ITC assays.

All the ITC calorimetric assays were carried out at 25 °C in the buffer, with a stirring speed of 750 rpm in an ITC-200 calorimeter (Malvern Instruments Ltd., UK). The reaction was initiated by injecting preloaded imipenem solution into the sample cell containing 210 µL enzyme solution; both are prepared in the same buffer. The reference cell of the calorimeter was loaded with the buffer only. Real-time thermal power changes were recorded continuously until the substrate was fully consumed. Data were plotted using Origin 2017 software.

Effect of chloride concentrations on the hydrolysis of imipenem by OXA-48$_{WT}$ and its variants.

To monitor the hydrolysis kinetic curves of imipenem by OXA-48_{WT} and its variants in the presence of various chloride ion concentrations, experiments were performed by injecting 10 µL imipenem solution (400 µM for OXA-48_{R214A}, 200 µM for other enzymes) into the sample cell filled with 210 µL of OXA-48_{WT} (100 nM), OXA-$48_{E185A/R186A/R206A}$ (100 nM), OXA-48_{AcK} (8 µM), OXA-48_{R250A} (2 µM) or OXA-48_{R214A} (3 µM), both are prepared in the same buffer (50 mM NaPi, pH 7.5, 1 mM $NaHCO_3$, with 0, 100, 400 mM NaCl). The concentrations in parentheses are the final concentrations.

UV-spectrometric assays.

The UV-spectrometric assays were performed in buffer 50 mM NaPi, pH 7.5, 1 mM $NaHCO_3$, using a Mapada UV-3100 spectrophotometer (Shanghai Mapada Instruments Co. Ltd., China) at 25 °C. The assay was carried out in a total volume of 1 mL with 100 nM OXA-48, 200 µM imipenem and a series dilution of chloride (0-800 mM). The decrease of imipenem absorbance was monitored continuously at 300 nm. Data were plotted using Origin 2017 Software.

Double injection assay.

To monitor the influence of additional substrate or enzyme on the hydrolysis kinetic curve during the steady state phase of the reaction, experiments were conducted in “multi-injection” mode. Both imipenem and OXA-48_{WT} stocks are in 50 mM NaPi, pH 7.5 buffer, containing 1 mM $NaHCO_3$ and 100 mM chloride. For the double injection assay of the imipenem substrate, 100 nM OXA-48_{WT} was preadded into the sample cell and 4.38 mM imipenem stock was loaded in the syringe. Two successive injections of imipenem (2 x 10 µL) into the sample cell were made at 740 s intervals. For the double injection assay of the enzyme, 200 µM imipenem was preadded in the sample cell and 1.1 µM OXA-48_{WT} stock was loaded in the syringe. Twice successive injections of enzyme (2 x 10 µL) into the sample cell were made at 510 s intervals.

Enzyme activity recovery assay.

A titration of different time intervals between two injections of imipenem was carried out to study the time required for the initial activity recovery before the 2nd injection of imipenem. Experiments were performed by injecting the first dose of imipenem solution into the sample cell filled with enzyme, both in the same buffer of 50 mM NaPi, pH 7.5, 1 mM $NaHCO_3$, with 100 or 400 mM NaCl. The same amount of imipenem is injected into the sample cell to initiate the second reaction after the first reaction at different intervals.

Size exclusion chromatography of OXA-48_{WT} and OXA-$48_{E185A/R186A/R206A}$.

Protein dimerization determinations were made by size exclusion chromatography on the GE AKTA Purifier 10 FPLC System (GE Healthcare) using a HiLoad 16/600 Superdex 200 pg column (GE Healthcare). The column was run in NaPi buffer 50 mM, pH 7.5 at a flow rate of 1.0 mL/min. A 1 mL

protein sample was injected and data analysis were performed using the UNICORN 5.20 software from GE. The retention time was converted to molecular weight according to the calibration curve.

Protein thermal shift assays.

Protein thermal shift assays were performed using the StepOne™ Real-Time PCR System (Applied Biosystems, Foster City, California, USA), with MicroAmp® Optical 8-Cap Strip (0.2 mL) from Applied Biosystems. Protein Thermal Shift™ Dye Kit (Thermo Fisher Scientific; Waltham, USA) was used. All pre-steps were setup on ice. A fresh Thermal Shift™ Dye (8x) was prepared by diluting the 1000x Thermal Shift™ with water. The reactions were prepared by pipetting all components into the wells in the following order: (1) 5.0 µL of Protein Thermal Shift™ buffer, (2) 2.5 µL protein stock (final concentration 12.5 µM), (3) 10 µL NaPi buffer (100 mM, pH 7.5, with 0, 80, 200 mM NaCl), (4) 2.5 µL fresh Protein Thermal Shift™ Dye (8x) to a total volume of 20.0 µL. The samples were assayed as triplicates in each measurement. The plate was centrifuged for 1 min at 1000 rpm to remove air bubbles and was kept on ice. The incubation was started at 25 °C for 2 min, followed by a temperature increase to 99 °C with a ramp-up rate of 1 °C/min. Melting curves were analyzed using the StepOne™ Software from Applied Biosystems. The melting temperature (T_m) was calculated from the inflexion point of the melting curve (derivative melting point).

IC50 and EC50 of all halide ions on OXA-48$_{WT}$.

The effect of halogen ions was examined with NaI, NaBr, NaCl, or NaF using 200 µM imipenem as substrate in a total volume of 1 mL buffer (50 mM NaPi, pH 7.5, with various concentrations of halide ions). Reactions were initiated by adding OXA-48$_{WT}$ to a final concentration of 100 nM and changes in absorbance of imipenem at 300 nm were recorded continuously by Mapada UV-3100 spectrophotometer (Shanghai Mapada Instruments Co. Ltd., China). The initial rate and the steady-state rate in the presence of a series of concentrations of halide ions were calculated. All experiments were done in triplicates. Half maximal inhibition concentration (IC_{50}) values in the steady-state reaction phase were calculated by plotting the percentage of inhibition against halide concentration; Half maximal effective concentration (EC50) values in the initial reaction phase were also determined by plotting the percentage of acceleration against halide concentration using GraphPad Prism 8 software[3].

Protein crystallization.

All OXA-48 proteins for crystallography were in 100 mM HEPES buffer, pH 7.5, and strictly no chloride present.

OXA-48$_{WT}$-I^- complex (PDB: 7O5T):

OXA-48$_{WT}$ apo protein crystals were grown at 18 °C using the hanging drop vapor diffusion method by mixing 1 µL of the protein stock (10 mg/mL) and an equal volume of precipitant containing 0.1 M

HEPES, pH 8.0, 10% 1-butanol,10% PEG8000. Well-diffracted crystals were soaked with 100 mM NaI for 1 min at 20 °C before crystals were cryoprotected by precipitant supplemented with 25% v/v glycerol and flash-frozen in liquid nitrogen.

OXA-48$_{E185A/R186A/R206A}$-Br^{-} apo crystal (PDB: 7PEI):

OXA-48$_{E185A/R186A/R206A}$ variant crystals were grown at 18 °C using the hanging drop vapor diffusion method by mixing 1 µL of the protein stock (10 mg/mL) and an equal volume of precipitant containing 0.2 M Li_2SO_4, 21% PEG3350. Well-diffracted crystals were soaked with 100 mM NaBr for 5 min at 20 °C. Precipitant containing 25% v/v glycerol was used as cryoprotectant before crystals were flash frozen in liquid nitrogen.

OXA-48$_{WT}$ apo (PDB: 7PEH), OXA-48$_{WT}$-Br^{-} (PDB: 7NRJ), and OXA-48$_{WT}$-HCO_3^{-} (PDB: 7O9N) complexes:

OXA-48$_{WT}$ apo crystals were grown at 18 °C using the sitting drop vapor diffusion method by mixing 1 µL of the protein stock (10 mg/mL) and an equal volume of precipitant containing 0.1 M HEPES, pH 7.5, 8% 1-butanol, and 11.6% w/v PEG8000. To obtain the OXA-48$_{WT}$-Br^{-} complex, well-diffracted crystals were soaked with 500 mM NaBr at 20 °C for 5 min. To obtain the OXA-48$_{WT}$-HCO_3^{-} complex, well-diffracted crystals were soaked with unbuffered 1 M $NaHCO_3$ at 20 °C for 30 min. Precipitant containing 20% v/v PEG400 was used as cryoprotectant before crystals were flash frozen in liquid nitrogen.

OXA-48$_{R250A}$-Br^{-} complex (PDB: 7PGO):

OXA-48$_{R250A}$ variant crystals were grown at 18 °C using the sitting drop vapor diffusion method by mixing 1 µL of the protein stock (10 mg/mL) and an equal volume of precipitant containing 0.1 M HEPES, pH 7.5, 8% 1-butanol, and 11.6% w/v PEG8000. Well-diffracted crystals were soaked with 250 mM NaBr at 20 °C. 20% v/v PEG400 was used as cryoprotectant before crystals were flash frozen in liquid nitrogen.

We deployed several strategies to capture an acyl-intermediate structure of OXA-48-imipenem with Br^{-} bound. We tried multiple times to add 500 mM NaBr first followed by 64 mM imipenem to the apo OXA-48$_{WT}$ crystal droplet, but only either hydrolyzed product (7PEP) or acyl-intermediate (7PSF) could be observed, with no detection of an anomalous signal from Br^{-} in the active site. But when we added 500 mM NaBr and 32 mM imipenem to the apo OXA-48$_{WT}$ crystal drop together, there was a clear Br^{-} anomalous signal overlapping with the density of imipenem to show Br^{-} and imipenem competing for binding to R250 (**Figure 1d**). The details are as follows:

OXA-48$_{WT}$-imipenem intermediate (PDB: 7PSF) and OXA-48$_{WT}$-imipenem product complexes with Br^{-} bound on the surface but not in the active site (PDB: 7PEP):

The OXA-48$_{WT}$-imipenem acyl-intermediate and product complexes were both obtained by soaking well-diffracted OXA-48$_{WT}$ apo crystals with 500 mM NaBr first, followed by 67.5 mM imipenem for 5-10 min. Please note, although no Br^- anomalous density was observed in the active site, Br^- density was consistently observed on the protein dimer interface near E185, R186, and R206, as well as on the protein surface near residues K137.

OXA-48 $_{WT}$-imipenem-Br^- product complexes with Br^- bound in the active site (PDB: 8QNZ):

The OXA-48$_{WT}$-imipenem-Br^- product complex was obtained by soaking well-diffracted crystals with 32 mM imipenem and 500 mM NaBr together as a premixed stock solution at 20 °C for 5-10 min. Precipitant containing 20% v/v PEG400 was used as cryoprotectant for crystals before crystals were flash frozen in liquid nitrogen. The anomalous density for Br^- was consistently observed in the active site near R250. The Br^- density was also observed on the protein dimer interface near E185, R186, and R206, as well as on the protein surface near residues R134, K137, and R174. As shown in **Figure 1d**, this product complex displays the imipenem 6-α-hydroxyethyl group in a different rotameric conformation from those in the OXA-48$_{AcK}$-imipenem-Br^- complex. This choice stems from the fact that the observed density for this group is not at full occupancy. In such cases, we fit the -OH group into the observed density due to its higher electronegativity compared to the methyl group, along with the proximity of a water molecule density.

OXA-48$_{AcK}$-imipenem (PDB: 7PFN), OXA-48$_{AcK}$-imipenem-Br^- (PDB: 7Q14) and OXA-48$_{AcK}$-oxacillin intermediate (PDB: 7PSE) complexes:

OXA-48$_{AcK}$ variant apo crystals were grown at 18 °C using the sitting drop vapor diffusion method by mixing 1 μL of the protein stock (10 mg/mL) and an equal volume of precipitant containing 0.1 M HEPES, pH 7.5, 8% 1-butanol, and 11.6% w/v PEG8000. To obtain the OXA-48$_{AcK}$-imipenem acyl-intermediate complex, well-diffracted crystals were soaked with 30 mM imipenem at 20 °C. The OXA-48$_{AcK}$-imipenem-Br^- intermediate complex was obtained by soaking OXA-48$_{AcK}$ variant apo crystals directly with imipenem powder and 250 mM NaBr at 20 °C for 5 to 10 min. To obtain the OXA-48$_{AcK}$-oxacillin acyl-intermediate complex, well-diffracted crystals were soaked with 30 mM oxacillin at 20 °C. Precipitant containing 20% v/v PEG400 was used as cryoprotectant before crystals were flash frozen in liquid nitrogen.

Crystallography data collection, processing and refinement.

Crystal diffraction data for structures of OXA-48$_{WT}$-I^- complex, OXA-48$_{E185A/R186A/R206A}$ apo, and OXA-48$_{WT}$-avibactam-Cl^- complex were collected at 100 K on beamline 17U1 of the Shanghai Synchrotron Radiation Facility (SSRF, Shanghai). Data were indexed and integrated using XDS[4] and HKL-3000[5], scaled with CCP4i2 suite[6] of programs. Crystal diffraction data for structures of apo OXA-48$_{WT}$, OXA-

48_{WT}-Br^- complex, OXA-48_{WT} -HCO_3^- complex, apo OXA-48_{R250A}, OXA-48_{AcK}-imipenem, OXA-48_{AcK}-imipenem-Br^- and OXA-48_{AcK}-oxacilin intermediate complexes, OXA-48_{WT}-imipenem intermediate and OXA-48_{WT}-imipenem product complexes, and OXA-48 $_{WT}$-imipenem-Br^- product complex was collected at 100 K on beamlines i03 and i04 of the Diamond Light Source UK. Reflections were automatically processed with the *xia2* pipeline[7] of the CCP4i2 software suite[6]. All structures were solved by molecular replacement using MOLREP[8] or Phaser[9] using OXA-48 PDB ID 4S2P as the search model. The structures were built and refined using iterative cycles with Crystallography Object-Oriented Toolkit (Coot[10]) and REFMAC5[11]. The NCS restraint parameters were used at later stages. Data collection and refinement statistics are provided in **Table S3**.

Michaelis-Menten kinetic for OXA-48_{WT} and OXA-48_{AcK73}.

Michaelis-Menten kinetics for OXA-48_{WT} and OXA-48_{AcK73} were measured for the enzyme-catalyzed hydrolysis of imipenem (Merck). The rates of hydrolysis of imipenem by 0.1 μM OXA-48_{WT} were monitored by following the UV absorption at 300 nm for the initial 30 seconds. All reactions were performed at 25 °C in the buffer of 100 mM NaPi, 50 mM $NaHCO_3$, pH 7.5, with strictly no chloride. 0-175 mM imipenem and the buffer solution were premixed in a 1 cm cuvette, and then the enzyme stock was added to a total volume of 1 mL. Kinetic parameters (k_{cat}, K_M, k_{cat}/K_M) were calculated using the Michaelis-Menten equation $y= E_t * k_{cat}*x/(K_M+x)$, from the GraphPad Prism 6.01 Software[12]. All experiments were performed in triplicate.

Molecular docking.

Covalent docking was performed using Molecular Operating Environment (MOE) [1]. Oxacillin (CID6196) coordinates were retrieved from the PubChem compound database[13]. The protein template is obtained from the H chain of the OXA-48_{AcK}-imipenem-Br^- structure. The imipenem ligand and water molecular were removed but the bromide ion was kept. Protein and ligand structures were prepared by using default parameters. The binding site was defined as S70 of the protein and the lactam ring-opening reaction was chosen as the reaction type. The figures of OXA-48_{AcK}-oxacillin complex in the inactive conformation were prepared using PyMOL[14].

Synthesis of N_α-(*tert*-butoxycarbonyl)-L-acetyllysine (**1**).

Commercially available N_α-(tert-butoxycarbonyl)-L-lysine (10 g, 40.6 mmol) was dissolved in NaOH (1 M, 100 mL) / THF (100 mL) and the mixture was cooled to 0 °C. Acetyl chloride (3.16 mL, 44.7 mmol) was added dropwise, and the reaction was stirred at room temperature. After 24 h, most of the THF was removed under reduced pressure. The resulting mixture was acidified to pH 4.0 with 1 M HCl and extracted with EtOAc (3 x 100 mL). The organic layer was dried over anhydrous $MgSO_4$, filtered and concentrated under reduced pressure to give the product N_α-(*tert*-butoxycarbonyl)-L-acetyllysine (**1**) as a white solid (5.5 g, 47%). **^{1}H NMR** (400 MHz, DMSO-d_6) δ 7.78 (t, J = 4.88 Hz, 1H), 6.99 (d, J =

6.4 Hz, 1H), 3.8 (m, 1H), 2.98 (m, 2H), 1.77 (s, 3H), 1.68–1.48 (m, 2H), 1.37 (s, 9H), 1.36–1.21 (m, 4H) ppm. **^{13}C NMR** (100 MHz, DMSO-d_6) δ = 174.2, 172, 155.6, 77.9, 53.4, 38.3, 30.5, 28.8, 28.2, 23.1, 22.6 ppm. **HRMS**: $C_{13}H_{24}N_2O_5$: [M - H]$^-$ = 287.1596.

Synthesis of L-acetyllysine (2).

N_α-(*tert*-butoxycarbonyl)-L-acetyllysine (4.8 g, 16.6 mmol) (**1**) was dissolved in DCM (50 mL). TFA (5.76 mL, 83 mmol) was added dropwise, and the reaction was stirred at room temperature. After 18 h, most of the TFA was removed under nitrogen and then completely removed under reduced pressure. The red oil was re-dissolved in deionized water (25 mL) and extracted with DCM (3 x 30 mL). The aqueous phase was concentrated under reduced pressure to give L-acetyllysine (**2**) as a yellow oil (7 g, 99 %). **^{1}H NMR** (400 MHz, D_2O) δ = 4.05 (t, *J* = 6Hz, 1H) 3.18 (t, *J* = 6.4 Hz, 2H), 2–1.85 (m, 2H), 1.97 (s, 3H), 1.56 (m, 2H), 1.44 (m, 2H) ppm. **^{13}C NMR** (100 MHz, D_2O) δ = 174, 172.2, 52.8, 38.8, 29.3, 27.7, 21.7, 21.5 ppm. **HRMS**: $C_8H_{16}N_2O_3$: [M + H]$^+$ = 189.1242.

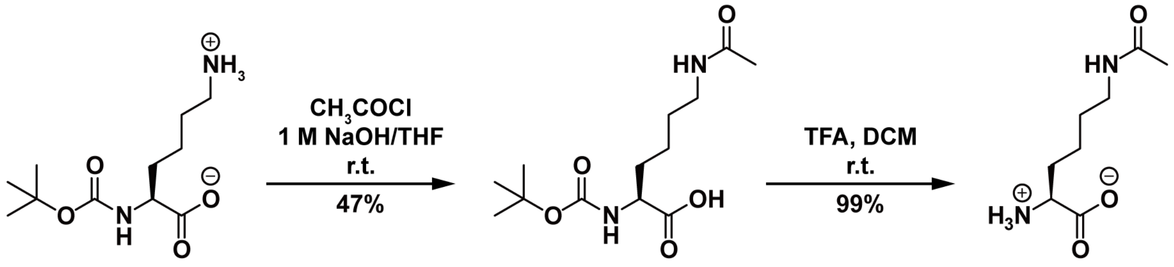

1 **2**

Scheme S1. Synthesis of L-acetyllysine (**2**).

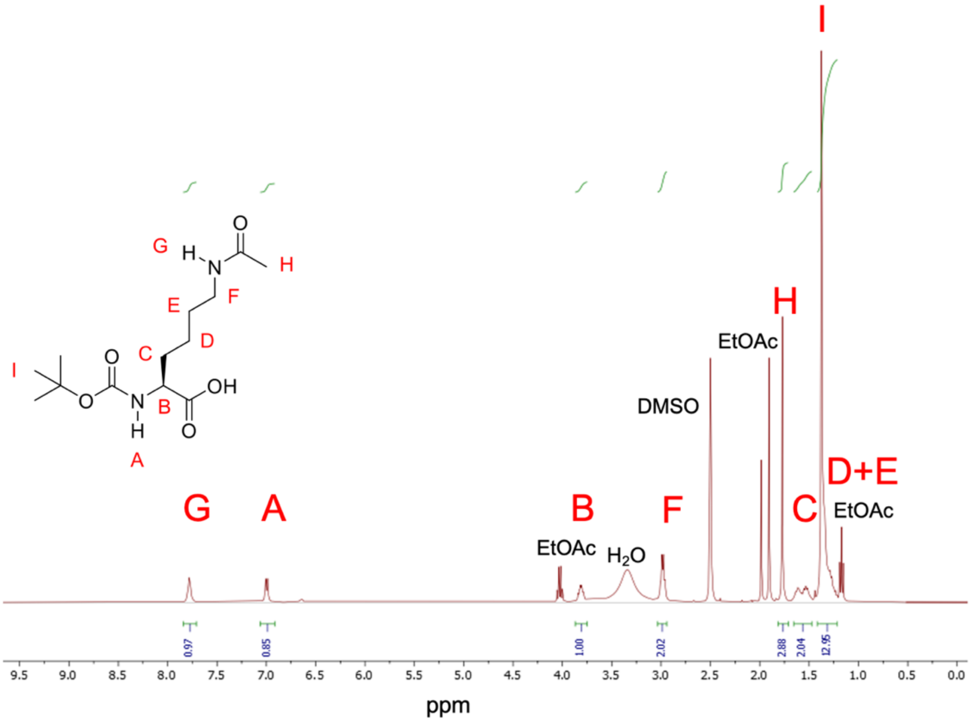

Figure S32 ^{1}H NMR spectrum of N_{α}-(*tert*-butoxycarbonyl)-L-acetyllysine (**1**) in DMSO-d_6 (400 MHz).

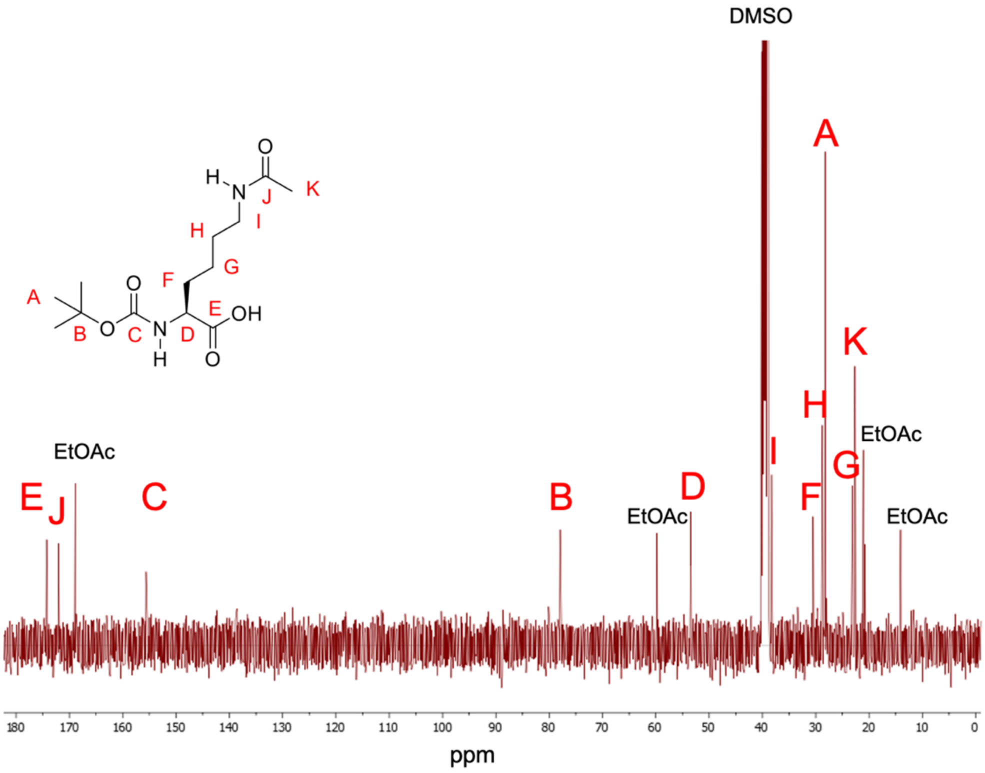

Figure S33 ^{13}C NMR spectrum of N_{α}-(*tert*-butoxycarbonyl)-L-acetyllysine (**1**) in DMSO-d_6 (100 MHz).

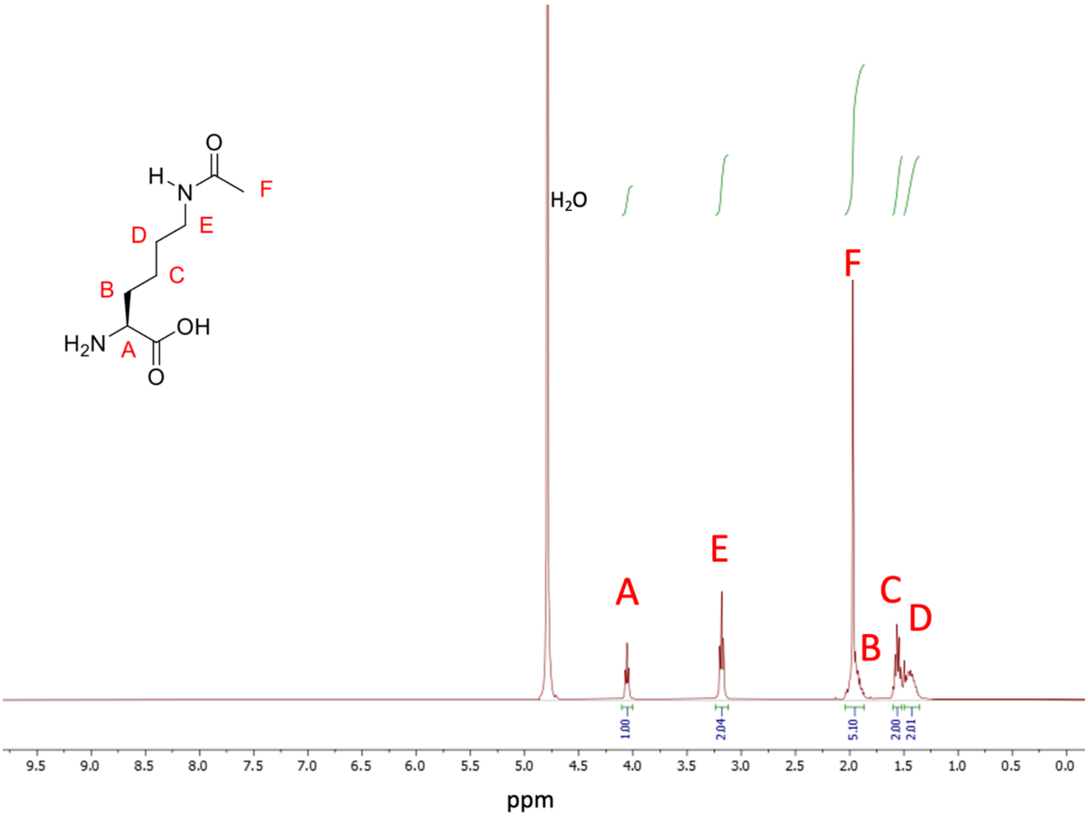

Figure S34 ^{1}H NMR spectrum of L-acetyllysine (**2**) in D_2O (400 MHz).

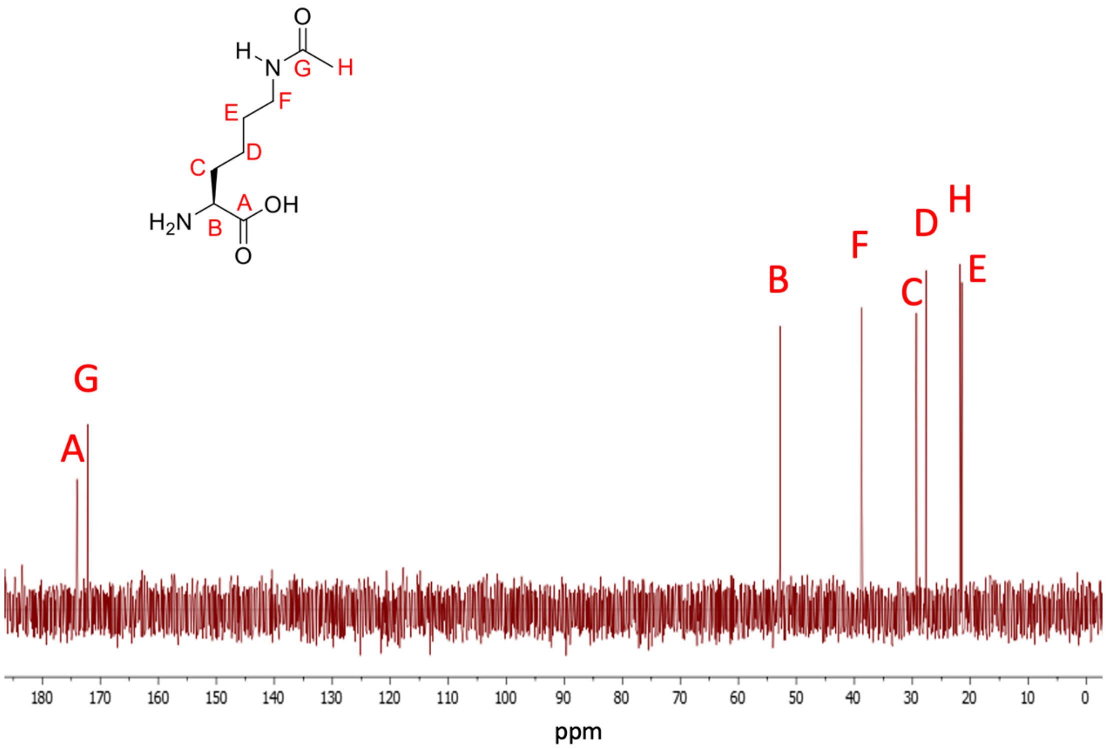

Figure S35 ^{13}C NMR spectrum of L-acetyllysine (**2**) in D_2O (100 MHz).

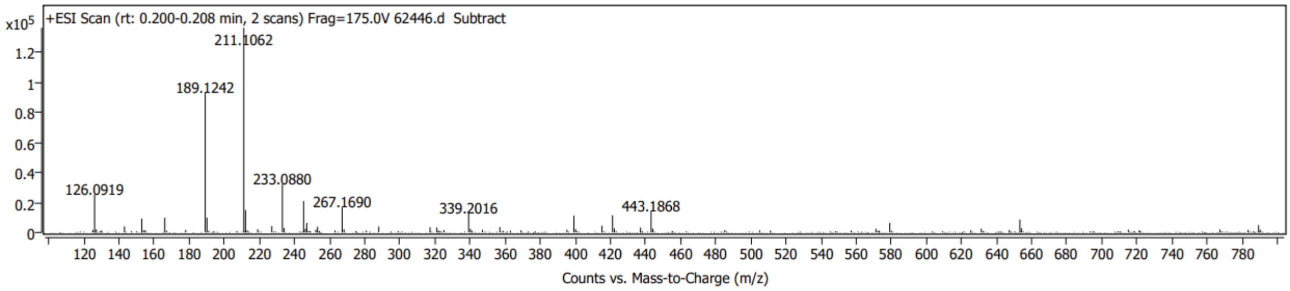

Figure S36 High-resolution MS of L-acetyllysine (**2**).

Mathematical modelling and numerical fitting

Note: This section and all the code needed to generate the figures and mathematical fits can be found at https://github.com/pablocatalan/oxa48.

We have shown that chloride binds OXA-48's dimer interface, which implies the reversible reaction $E + 2Cl^- \rightleftharpoons E^{Cl}$ where E^{Cl} is the enzyme with more than two chloride ions in the interface and on the surface. Since the concentration of chloride is much larger than that of enzyme in our experiments, it is reasonable to suppose that most of the enzyme is in E^{Cl} form, and we will consider only this form in the following.

This form of the enzyme undergoes the usual reactions, $E^{Cl} + S \xrightarrow{k_1'} E^{Cl}A \xrightarrow{k_2'} E^{Cl} + P + H,$ where k_1' and k_2' could in principle depend on chloride.

In addition, $E^{Cl}A$ undergoes two extra reactions $E^{Cl}A + Cl^- \underset{k_{-3}'}{\overset{k_3'}{\rightleftharpoons}} E^{Cl}A.Cl$, forming an inactive intermediate complex $E^{Cl}A.Cl$ as the forward reaction, and Cl⁻ dissociating from the inactive intermediate complex as the reverse reaction.

In the presence of chloride, the new mathematical model is then

$$
\begin{aligned}
\frac{d[E^{Cl}]}{dt} &= -k_1'[E^{Cl}][S] + k_2'[E^{Cl}A], \\
\frac{d[S]}{dt} &= -k_1'[E^{Cl}][S], \\
\frac{d[E^{Cl}A]}{dt} &= k_1'[E^{Cl}][S] - k_2'[E^{Cl}A] - k_3'[E^{Cl}A][Cl^-] + k_{-3}'[E^{Cl}A.Cl], \\
\frac{d[E^{Cl}A.Cl]}{dt} &= k_3'[E^{Cl}A][Cl^-] - k_{-3}'[E^{Cl}A.Cl], \\
\frac{dH}{dt} &= -ck_2'[E^{Cl}A].
\end{aligned}
$$

For simplicity of notation, we will drop the bracket notation, using E for both forms of the enzyme, C for the active intermediate $E^{Cl}A$ and C_2 for the inactive intermediate $E^{Cl}A.Cl$. The model then becomes

$$\frac{dE}{dt} = -k_1'ES + k_2'C,$$

$$\frac{dS}{dt} = -k_1' ES,$$
$$\frac{dC}{dt} = k_1' ES - k_2' C - k_3' \mathrm{Cl}^- C + k_{-3}' C_2,$$
$$\frac{dC_2}{dt} = k_3' \mathrm{Cl}^- C - k_{-3}' C_2,$$
$$\frac{dH}{dt} = -c k_2' C,$$

Where k_1', k_2', k_3', and k_{-3}' are apparent reaction rate constants that would be taken as functions of chloride. In the following, we will use mathematical arguments to discuss the effect of chloride ion concentration on them. We have also performed 50 independent numerical fits of these four parameters to each chloride treatment (300 parameter sets in total), using a custom simulated annealing optimization algorithm[15]. Simulated annealing is a Monte Carlo method that tries to find the best fit to a dataset starting from an initial guess and iteratively choosing one parameter to change at random. If changing a parameter improves the fit, the new parameter set is accepted. If it doesn't improve the fit, the algorithm accepts the new set with a probability lower than one: this is done so that the algorithm is not stuck at suboptimal parameter sets and allows it to find better fits. The particularity of simulated annealing when compared with other Monte Carlo approaches is that here the probability of accepting a new parameter set decreases as the number of iterations increases, mimicking a decrease in the "temperature" of the system[16]. Note that simulated annealing tries to fit all parameters at the same time, thus obtaining a coherent parameter set that is consistent with the data. The resulting curves are shown in the main text (**Fig. 4b**).

Working with the "real" heat curve

The shape of titration curves is affected by the calorimeter response time[17]. The signal measured by the calorimeter decreases exponentially with a characteristic time t_{ITC}, and so the measured heat release $P_m(t)$ is a convolution between the real signal $P_s(t) = dH/dt$ and an exponential kernel $R(t) = (t_{ITC})^{-1} \exp(-t/t_{ITC})$. Thus,

$$P_m(t) = \frac{1}{t_{ITC}} \int_0^t \exp(-(t-\tau)/t_{ITC}) P_s(\tau)\, d\tau, \qquad (1)$$

which can be solved either by differentiating once or using the Laplace transform, yielding

$$t_{ITC}\frac{dP_m}{dt} + P_m = P_s(t). \quad (2)$$

Given a measured signal $P_m(s)$, we can numerically obtain from this equation the "real" signal that corresponds to our dynamic equations. In our experiments, $t_{ITC} = 8\,s$, given by the manufacturer of ITC-200 calorimeter (Malvern Instruments Ltd., UK). The "real" and "measured" signals are quite similar, but there are relevant differences especially when there are spikes of heat release, such as at the beginning of the titration experiment.

In the following, we will use the "real" signal $P_s(t)$ to obtain kinetic rates and then use these to simulate our model. In order to compare with the experimental datasets, we will then transform our mathematical variable dH/dt to a measured P_m, using the differential equation above. As seen in **Fig. 2**, the ITC data shows a baseline drift, but we will not correct it, as it will not significantly alter our main conclusions.

Dealing with the finite injection time of the substrate

A common problem when modelling ITC curves comes from the fact that the injection of the substrate into the sample cell is not instantaneous, but rather requires a finite amount of time. Typically, this injection is also combined with fast mixing, so that a homogeneous concentration of substrate is quickly attained in the sample cell. Writing this out mathematically is a challenge: ideally, we could derive a partial differential equation that would incorporate the diffusion into the sample cell but deriving a mathematical expression for the mixing term seems rather hard.

Since our main purpose is to extract the reaction rates of imipenem by OXA-48, we have found a simple, alternative way to model the diffusion of the substrate throughout the ITC sample cell by phenomenologically modelling a linear injection into the system. The equation for E then becomes

$$\frac{dE}{dt} = \frac{E_T}{k_0}(1 - \Theta(t - k_0)) - k_1 ES + k_2 C,$$

where $\Theta(t)$ is the Heaviside function that has value 0 if $t < 0$, and 1 if $t > 0$. k_0 is the time needed for the substrate to diffuse, and we will also fit its value using our numerical algorithm, obtaining values between 23 and 33 seconds depending on the experiment.

Finding out c

Integrating the area under any of our experimental ITC curves, we obtained the total heat released when all the substrate has been hydrolysed. We would get the same value by multiplying the initial substrate concentration ($S_0 = 200\mu M$) times c, the conversion rate. Thus, we can estimate c by dividing the total heat released by S_0. Using this method, in our ITC experiments with different chloride concentrations, we got values of c between 4.5 and 5.5 calories per mole, increasing linearly with chloride concentration (**Fig. S17a**).

As there is no binding heat from chloride, the variability could then come from an intrinsic drift of the baseline inherent from ITC as a technique. This drift worsens with the length of the experiment: if the increase in *c* values was due to the baseline drift, we would expect *c* to increase with the length of the ITC curve (measured in seconds), and this is what we observe (**Fig. S17b**). Therefore, we will use the estimated value of c for each chloride concentration in the following sections. We also allow our simulated annealing algorithm to fit the values of c, to account for errors induced by the drift in the ITC baseline, finding values similar to our estimates.

<u>Relationship between k_2' and chloride</u>

When repeating the experiment with nonzero concentrations of chloride in solution, we find that the maximum heat release increases as chloride increases. As both c and E_T are the same throughout all experimental conditions, it must be the case that k_2’ is increasing with chloride.

At the point of maximal heat release, we assume that all the enzyme is in C form, and so

$$(dH/dt)_{max} = ck_2' E_T \implies k_2' = \frac{(dH/dt)_{max}}{cE_T}.$$

From this equation we can infer the values of k_2' from the data and using these values as seeds for our simulated annealing algorithm, we find a concave, monotonous relationship between *k*2’ and chloride, described by the equation $k_2' = 2.9 + 5.0\frac{\left(\frac{\mathrm{Cl}^-}{120.8}\right)^2}{1+\left(\frac{\mathrm{Cl}^-}{120.8}\right)^2}$, R^2=0.96 (**Fig. 4d**).

<u>Finding out k_3' and k_{-3}'</u>

When all of the enzyme is in C state, the first two terms in the equation for dC/dt are approximately zero, and so we can limit the dynamics to

$$dC/dt = -k_3'\text{Cl}^-C + k_{-3}'C_2,$$
$$dC_2/dt = k_3'\text{Cl}^-C - k_{-3}'C_2.$$

This is a linear system of two ODEs. Taking initial conditions $C(0) = E_T, C_2(0) = 0$, the solution is

$$C(t) = E_T\frac{k_{-3}'}{k_3'\text{Cl}^- + k_{-3}'} + E_T\frac{k_3'}{k_3'\text{Cl}^- + k_{-3}'}e^{-(k_3'\text{Cl}^- + k_{-3}')t}.$$

Now, since $dH/dt = -ck_2C$, in order to find k_3' and k_{-3}' we just need to fit the previous equation to our heat data, between the maximum and the stationary value (**Fig. S18**). Using these as initial seeds for our simulated annealing algorithm, we found an inverse relationship between both k_3' and k_{-3}' and chloride, given by k_3' = 10.14/Cl^- μM^{-1} s^{-1}, R^2=0.87 (**Fig. 4e**) and k_{-3}' = 0.57/Cl^- s^{-1}, R^2 = 0.96 (**Fig. 4f**).

In the literature[18], the terms v_0 and v_s are used for the rates of the product creation when there is no inhibition and when the stationary phase has been reached, respectively. The correspondence with our derivations is $v_0 = k_2'E_T$ and $v_s = k_2'E_T\frac{k_{-3}'}{k_3'\text{Cl}^- + k_{-3}'}$.

All combined, our simulated annealing algorithm tries to find a combination of six parameters that can fit the data simultaneously: *c*, k_0, k_1', k_2', k_3' and k_{-3}'. The fact that our algorithm yields similar values for each of these parameters in every iteration (of fifty, see **Fig. 4c-f**) suggests that we are not overfitting the model.

<u>The relationship between k_1' and chloride</u>

As pointed out before, there is no easy way to obtain information about k_1' from the equations. However, we can find relevant information from our numerical fits. Using different initial seeds for this parameter (as we had no mathematical arguments to constrain its value) we consistently obtain an inverse relationship between k_1' and chloride, given by $k_1' = \frac{258.1}{\text{Cl}^- + 184.2}$, R^2=0.94 (**Fig. 4c**).

<u>Fitting the ITC curves</u>

For each ITC curve in each of the experiments, we applied the previously described methods to obtain the apparent rates (**Fig. S20**). For the exact values of the parameters used in these simulations, see github.com/pablocatalan/oxa48.

Simulating a non-zero hydrolysis rate from the inactive intermediate

We asked whether the inactive intermediate is also able to hydrolyse imipenem. This new model would include a new reaction $C_2 \xrightarrow{k_5} E + P + H$ with a rate k_5. The model then becomes

$$
\begin{aligned}
\frac{dE}{dt} &= \frac{E_T}{k_0}(1 - \Theta(t - k_0)) - k_1' ES + k_2' C + k_5 C_2, \\
\frac{dS}{dt} &= -k_1' ES, \\
\frac{dC}{dt} &= k_1' ES - k_2' C - k_3' \mathrm{Cl}^- C + k_{-3}' C_2, \\
\frac{dC_2}{dt} &= k_3' \mathrm{Cl}^- C - k_{-3}' C_2 - k_5 C_2, \\
\frac{dH}{dt} &= -c(k_2' C + k_5 C_2),
\end{aligned}
$$

We then simulated this model for different values of k_5 and the values of the other parameters. We see that this new model fails to capture the ITC data even for very low values of k_5 from 0 to 0.5 s^{-1}, which suggests that there is no hydrolysis from the inactive intermediate (**Fig. S21**).

Simulating the dynamics for the interface mutant

With the data in **Fig. S10c, d**, we use our mathematical analysis to study the apparent reaction rates and their relationship to chloride concentration in the interface mutant OXA-$48_{E185A/R186A/R206A}$ as well (**Fig. S22**). Simulation plots from fifty parameter sets per chloride condition obtained with our numerical algorithm (dashed lines) agree quite well with the data (solid lines). Parameter values are shown in **Table 1**.

References:

1 Molecular Operating Environment (MOE), 2022.02 Chemical Computing Group ULC, 1010 Sherbooke St. West, Suite #910, Montreal, QC, Canada, H3A 2R7, 2022.

2 Zhang, Y. *et al.* Label-free visualization of carbapenemase activity in living bacteria. *Angewandte Chemie, International Edition* **57**, 17120-17124 (2018).

3 GraphPad Prism version 8.0.1 for Windows, GraphPad Software, San Diego, California USA, .

4 Kabsch, W. XDS. *Acta Crystallographica Section D* **66**, 125-132 (2010).

5 Minor, W., Cymborowski, M., Otwinowski, Z. & Chruszcz, M. HKL-3000: the integration of data reduction and structure solution-from diffraction images to an initial model in minutes. *Acta Crystallographica Section D* **62**, 859-866 (2006).

6 Potterton, L. *et al.* CCP4i2: the new graphical user interface to the CCP4 program suite. *Acta Crystallographica Section D* **74**, 68-84 (2018).

7 Winter, G. xia2: an expert system for macromolecular crystallography data reduction. *Journal of Applied Crystallography* **43**, 186-190 (2010).

8 Vagin, A. & Teplyakov, A. Molecular replacement with MOLREP. *Acta Crystallographica Section D* **66**, 22-25 (2010).

9 Mccoy, A. J. *et al.* Phaser crystallographic software. *Journal of Applied Crystallography* **40,** 658-674 (2007).

10 Emsley, Paul, Cowtan & Kevin. Coot: model-building tools for molecular graphics. *Acta Crystallographica: Section D* **60**, 2126-2132 (2004).

11 Murshudov, G. N. *et al.* REFMAC5 for the refinement of macromolecular crystal structures. *Acta Crystallographica Section D* **67**, 355-367 (2011).

12 GraphPad Prism version 6.0.1 for Windows, GraphPad Software, San Diego, California USA, .

13 Bolton, E. E., Wang, Y., Thiessen, P. A. & Bryant, S. H. Chapter 12 - PubChem: integrated platform of small molecules and biological Activities. *Annual Reports in Computational Chemistry* **4**, 217-241 (2008).

14 The PyMOL Molecular Graphics System, Version 2.5, Schrodinger, LLC. (2021).

15 Nieto, C. et al, COP1 dynamics integrate conflicting seasonal light and thermal cues in the control of Arabidopsis elongation. *Science Advances,* **8**, eabp8412 (2022).

16 Kirkpatrick et al. Optimization by simulated annealing. *Science* **220**: 671-680 (1983).

17 Burnouf, D. *et al.* kinITC: A new method for obtaining joint thermodynamic and kinetic data by isothermal titration calorimetry. *Journal of the American Chemical Society* **134**, 559-565 (2012).

18 Ji, Z. *et al.* Protein Electric Fields Enable Faster and Longer-Lasting Covalent Inhibition of β-Lactamases. *Journal of the American Chemical Society*. **144**, 20947-20954 (2022).
